# Locating, tracing and sequencing multiple expanded genetic letters in complex DNA context via a bridge-base approach

**DOI:** 10.1093/nar/gkad218

**Published:** 2023-03-27

**Authors:** Honglei Wang, Wuyuan Zhu, Chao Wang, Xiaohuan Li, Luying Wang, Bianbian Huo, Hui Mei, Anlian Zhu, Guisheng Zhang, Lingjun Li

**Affiliations:** Henan Key Laboratory of Organic Functional Molecule and Drug Innovation, Collaborative Innovation Center of Henan Province for Green Manufacturing of Fine Chemicals, School of Chemistry and Chemical Engineering, Key Laboratory of Green Chemical Media and Reactions, Ministry of Education, Henan Normal University, Xinxiang, Henan 453007, China; State Key Laboratory of Cell Differentiation Regulation and Target Drug, Henan Normal University, Xinxiang 453007, China; Henan Key Laboratory of Organic Functional Molecule and Drug Innovation, Collaborative Innovation Center of Henan Province for Green Manufacturing of Fine Chemicals, School of Chemistry and Chemical Engineering, Key Laboratory of Green Chemical Media and Reactions, Ministry of Education, Henan Normal University, Xinxiang, Henan 453007, China; Henan Key Laboratory of Organic Functional Molecule and Drug Innovation, Collaborative Innovation Center of Henan Province for Green Manufacturing of Fine Chemicals, School of Chemistry and Chemical Engineering, Key Laboratory of Green Chemical Media and Reactions, Ministry of Education, Henan Normal University, Xinxiang, Henan 453007, China; Henan Key Laboratory of Organic Functional Molecule and Drug Innovation, Collaborative Innovation Center of Henan Province for Green Manufacturing of Fine Chemicals, School of Chemistry and Chemical Engineering, Key Laboratory of Green Chemical Media and Reactions, Ministry of Education, Henan Normal University, Xinxiang, Henan 453007, China; Henan Key Laboratory of Organic Functional Molecule and Drug Innovation, Collaborative Innovation Center of Henan Province for Green Manufacturing of Fine Chemicals, School of Chemistry and Chemical Engineering, Key Laboratory of Green Chemical Media and Reactions, Ministry of Education, Henan Normal University, Xinxiang, Henan 453007, China; Henan Key Laboratory of Organic Functional Molecule and Drug Innovation, Collaborative Innovation Center of Henan Province for Green Manufacturing of Fine Chemicals, School of Chemistry and Chemical Engineering, Key Laboratory of Green Chemical Media and Reactions, Ministry of Education, Henan Normal University, Xinxiang, Henan 453007, China; State Key Laboratory of Cell Differentiation Regulation and Target Drug, Henan Normal University, Xinxiang 453007, China; Shenzhen Key Laboratory of Synthetic Genomics, Guangdong Provincial Key Laboratory of Synthetic Genomics, CAS Key Laboratory of Quantitative Engineering Biology, Shenzhen Institute of Synthetic Biology, Shenzhen Institutes of Advanced Technology, Chinese Academy of Sciences, Shenzhen 518055, China; Henan Key Laboratory of Organic Functional Molecule and Drug Innovation, Collaborative Innovation Center of Henan Province for Green Manufacturing of Fine Chemicals, School of Chemistry and Chemical Engineering, Key Laboratory of Green Chemical Media and Reactions, Ministry of Education, Henan Normal University, Xinxiang, Henan 453007, China; Henan Key Laboratory of Organic Functional Molecule and Drug Innovation, Collaborative Innovation Center of Henan Province for Green Manufacturing of Fine Chemicals, School of Chemistry and Chemical Engineering, Key Laboratory of Green Chemical Media and Reactions, Ministry of Education, Henan Normal University, Xinxiang, Henan 453007, China; Henan Key Laboratory of Organic Functional Molecule and Drug Innovation, Collaborative Innovation Center of Henan Province for Green Manufacturing of Fine Chemicals, School of Chemistry and Chemical Engineering, Key Laboratory of Green Chemical Media and Reactions, Ministry of Education, Henan Normal University, Xinxiang, Henan 453007, China; State Key Laboratory of Cell Differentiation Regulation and Target Drug, Henan Normal University, Xinxiang 453007, China

## Abstract

A panel of unnatural base pairs is developed to expand genetic alphabets. One or more unnatural base pairs (UBPs) can be inserted to enlarge the capacity, diversity, and functionality of canonical DNA, so monitoring the multiple-UBPs-containing DNA by simple and convenient approaches is essential. Herein, we report a bridge-base approach to repurpose the capability of determining TPT3-NaM UBPs. The success of this approach depends on the design of isoTAT that can simultaneously pair with NaM and G as a bridge base, as well as the discovering of the transformation of NaM to A in absence of its complementary base. TPT3-NaM can be transferred to C–G or A–T by simple PCR assays with high read-through ratios and low sequence-dependent properties, permitting for the first time to dually locate the multiple sites of TPT3-NaM pairs. Then we show the unprecedented capacity of this approach to trace accurate changes and retention ratios of multiple TPT3-NaM UPBs during *in vivo* replications. In addition, the method can also be applied to identify multiple-site DNA lesions, transferring TPT3-NaM makers to different natural bases. Taken together, our work presents the first general and convenient approach capable of locating, tracing, and sequencing site- and number-unlimited TPT3-NaM pairs.

## Graphical Abstract

**Graphical Abstract:**
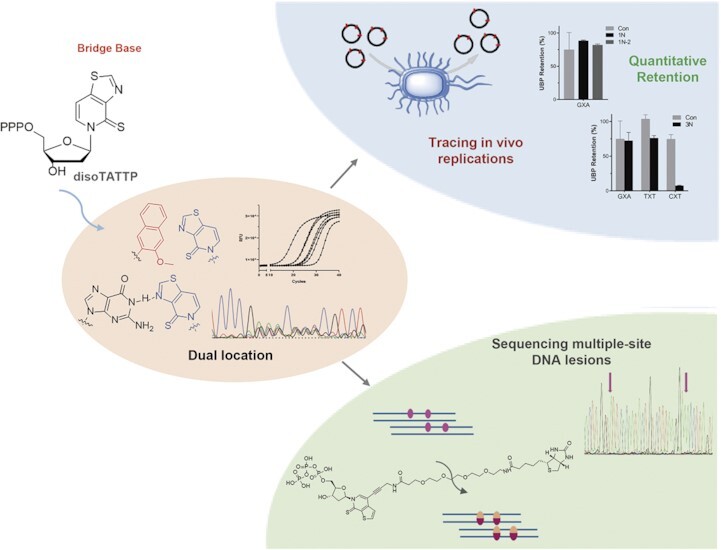
We developed a bridge-base approach with high read-through ratios and low sequence-dependent properties for the first time to dually locate the multiple sites of TPT3-NaM pairs. This approach is also capable of tracing in vivo replications of TPT3-NaM pairs and sequencing multiple site DNA lesions marked with the unnatural base pairs.

## INTRODUCTION

Naturally, genetic information is stored in a four-letter A/T/G/C alphabet. Strenuous efforts have been made toward the design and synthesis of unnatural base pairs (UBPs) to expand the genetic letters (1–3), by now generating a panel of promising pairs including P-Z, Ds-Px and TPT3-NaM (4–8). Unique to the family of unnatural base pairs is their ability to act as bioorthogonal genetic letters for the replication and storage of genetic information (9,10). For example, the systematic evolution of ligands by exponential enrichment (SELEX) with DNA library bearing multiple UBPs is proven to effectively gain affinity-enhanced aptamers at pmol levels (11–13). Encoding three noncanonical amino acids by UBP-bearing codon and anticodon system (A-NaM-C as condon, G-TPT3-T as anticodon) has been succeeded in the expression of sfGFP proteins (14). More recently, an in-depth combination of UBPs and natural genetic letters has also been implemented for finding three new unnatural genetic codons, by which AGX, GXT and AXC (X = NaM) could be simultaneously and orthogonally decoded for protein expressions in semi-synthetic organisms (15). Insertion of more unnatural base pairs can enlarge the capacity, diversity, as well as functionality of canonical DNA (16–18), however, monitoring the multiple-UBPs-containing DNA by simple and convenient approaches is challenging. In addition, the *in vivo* efficiency of more than one UBP in DNA sequences shows more contingent during their replications in cells (14,15), which raises a new requirement to monitor the retention of multiple UBPs.

TPT3-NaM is one of the advanced UPBs in the expanded genetic letters (2,19,20). Up to now, biotin shift and sequencing assays are developed to monitor DNA containing TPT3-NaM (2,21,22). However, the biotin shift assay is unable to detect multiple coding UBPs arranged tightly or UBPs with non-default loci and is also incompetent to monitor the site mutation near the UBPs (14,15), which may play an important role in precise protein expression. Sequencing assays can be divided into Sanger and nanopore sequencing. Sanger sequencing can detect a single UBP where the signal terminal can give the correct positions of unnatural bases (8,21). But the main obstacle to sequencing multiple UBPs in DNA is the disappearance of the Sanger sequencing signals after the first UBP site which makes the subsequent information hard to be acquired. Alternatively, the nanopore sequencing method has been developed to differentiate each unnatural base based on their different shapes (22,23), but the method involved tedious modifications on UBPs, specialized protein preparation procedures, and expansive apparatus, which possibly limits the general applications of this method. So the way for evaluation of complex DNA products with two or more TPT3-NaM UBPs at adjacent coding regions or with non-default positions, including calculation of their retentions, has remained undemonstrated thus far.

**Figure 1.**
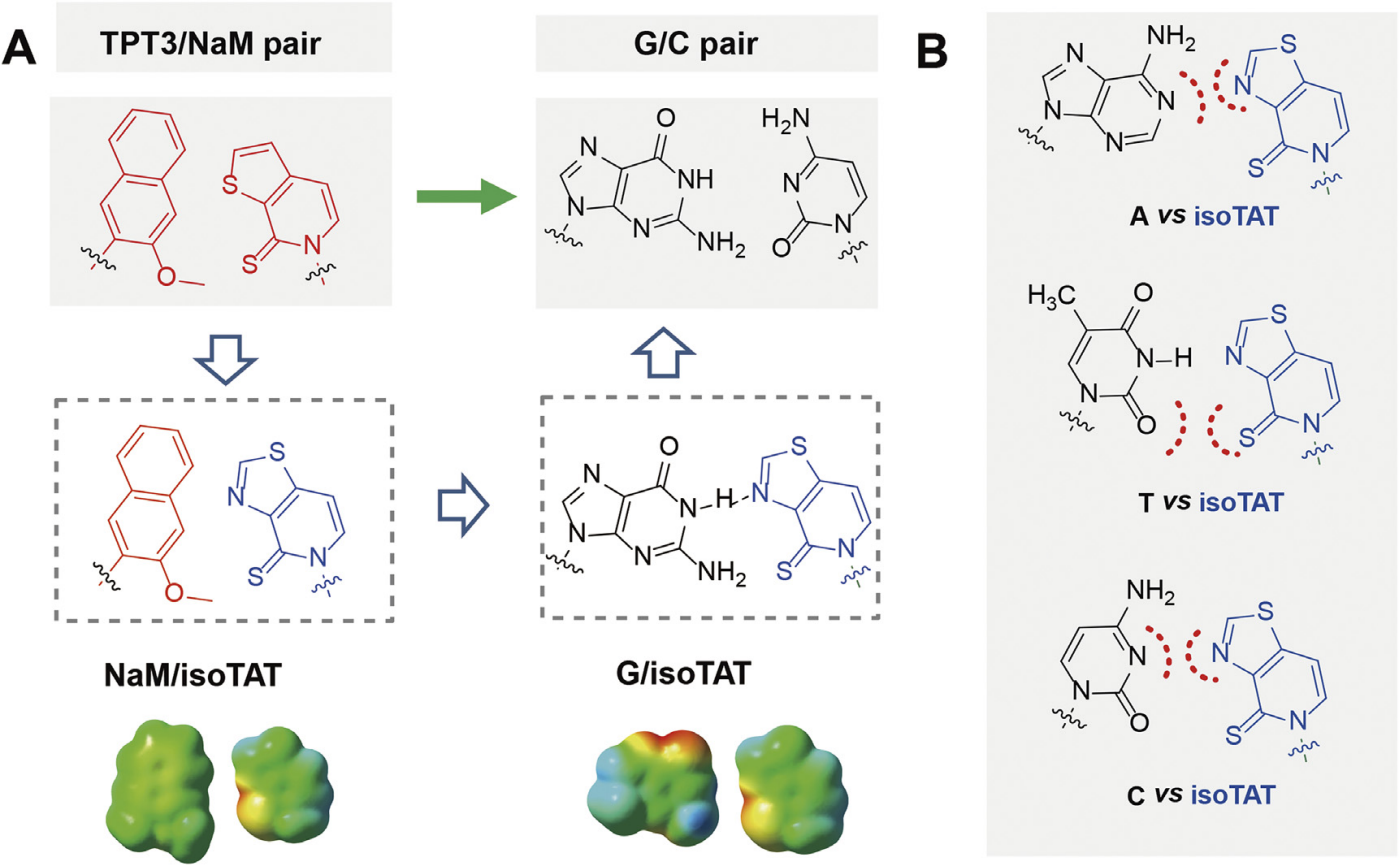
Design of isoTAT (blue) for the specific transformation of NaM-TPT3 pair to G–C pair. (**A**) The chemical structures of unnatural NaM-TPT3, NaM-isoTAT, and hybrid G-isoTAT base pairs. Space-filling models with electrostatic potential maps are shown (DFT calculations at the B3LYP/6–31 + G(d) level, Gaussian 09), with methyl in place of deoxyribose. The electron-rich and electron-deficient areas are marked in red and green, respectively. (**B**) The chemical structures of unpaired C-isoTAT, A-isoTAT and T-isoTAT base pairs with the potential repulsion of like electric charges (red dotted line).

Here, we design a bridge base (isoTAT) to transfer the TPT3-NaM pair to C–G natural pair exclusively (Figure 1A), which can be used to read through all the UBPs in DNA sequences. And we also demonstrate the transformation of NaM to A in the absence of its complementary base. Our results show that the efficient transformation of TPT3-NaM to C–G or A–T can be implemented by two PCR assays with high read-through ratios and low sequence-dependent properties, thus allowing for the first time to dually locate the multiple sites of TPT3-NaM pairs. Importantly, we further indicate the read-through capacity and transferring ability of the bridge base can be used to evaluate the retention rates of multiple TPT3-NaM UBPs at adjacent sites, and identify the accurate retention ratios of the adjacent unnatural bases in SSO replications. In addition, the approach is also practical to determine multiple DNA lesions marked by TPT3-NaM and then transform TPT3-NaM to different natural bases via PCR.

## MATERIALS AND METHODS

### Materials and analytic methods

Oligonucleotides with a length > 100 bp were purchased from GenScript, and other oligonucleotides were purchased from Sangon Biotech (see sequences used in this study). dNTPs were purchased from Solarbio. Klenow fragment DNA polymerase I was purchased from ABclonal Biotech Co., Ltd. OneTaq DNA polymerase, Deep vent DNA polymerase, glycosylase (UDG), apurinic/apyrimidinic Endonuclease 1 (APE1), as well as T4-DNA ligase, were purchased from New England Biolabs. 2 × UItraSYBR Mixture was purchased from CWBIO Biotech co., Ltd. pBLUE-T Fast Cloning Kits and BL 21 (DE3) Electrocompetent cells were purchased from Zoman Biotechnology Co., Ltd. dNaMTP, dTPT3TP, and dTPT3^PA^ were synthesized as reported (8,24). NMR spectra were performed on AVANCE NanoBay (400 MHz). HRMS or MS were performed on Bruker compact Ultra-high-resolution electro-spray time-of-flight mass spectrometry and Bruker Autoflex speed MALDLTOF/TOF spectrometry, respectively.

### Pre-steady state kinetic assays

This assay was performed according to our previous report with some modifications (25). Briefly, after the annealing of primers (labeled with HEX) and templates, the incorporation reactions were initiated by adding 3 × dYTP (1.5 μM, 10 μl) to a 20 μl solution containing 113 nM primer/template and 4.5 U Klenow fragment DNA polymerase I. Reactions were set up at 37°C for 15 s, the reactions were quenched with 10 μl of 50 mM EDTA. For the extension assays, dCTP was added to the reaction system immediately to a concentration of 2 μM after the incorporation reaction, then incubated for another 15, 30 or 60 s, and finally quenched with 10 μl of 50 mM EDTA. Only isoTAT was also extended with NaMG and GG templates with different concentrations and time-coursing. All solutions were in Kf buffer (10 mM Tris–HCl, 50 mM NaCl, 10 mM MgCl_2_, 1 mM DTT, pH 7.9 at 25°C). The reaction solution was concentrated, mixed with enough loading dye (90% formamide, and sufficient amounts of bromophenol blue and xylene cyanol), and analyzed by 8 M urea 15% denaturing polyacrylamide gel electrophoresis. The primer strips were imaged and quantified by AI600 software (Amersham Imager 680). The data are averages and standard deviations of three independent determinations. GraphPad Prism 8 was used to plot the data.

### Steady-state kinetic assays

The steady-state kinetic parameters were monitored according to the previous report with some changes (24,26). The reaction system and operation were the same as the incorporation assays above with changes in the total amount of Kf enzyme (0.225 U), the concentrations of dYTP or dNTP (the final concentrations ranging from 0.0039–100 μM), and the incubation time changed to 10 s. The steady-state kinetic constants for each base pair were calculated from eight concentrations vs velocities using Michaelis − Menten equation by GraphPad Prism 8. Velocities were calculated by the fractions of extended primer/min (%incor/min) with all the fractions of extended primer <20% at each concentration. The data are the averages and standard deviations of three independent experiments.

### Transformed PCR and retention assays

Quantitative real time PCR (qPCR) analysis with 2 × UItraSYBR was based on the Manufacturer's method with the addition of isoTAT and NaM or only NaM to a concentration of 0.1 mM following the thermal cycling conditions: 95°C, 10 min; (95°C, 15 s; 60°C, 1.5 min) ×40. OneTaq DNA polymerase was used to perform all other PCR assays. First, PCR was performed to obtain doubled strand templates under the following conditions (in a volume of 25 μl): 1× reaction buffer with OneTaq DNA Polymerases, 0.2 mM dNTP, 0.1 mM dNaMTP and dTPT3TP, 0.4 ng of the single strand 134-mer templates (nature, 1N, 2N, 3N, or UN), and 400 nM primers under the following thermal cycling conditions: 16× (96°C, 10 s; 60°C, 15 s; 68°C, 2 min for 3N template or 68°C, 1 min for other templates), and then a final extension (68°C, 5 min). The PCR products were purified with a QIAquick Gel Extraction Kit (QIAGEN) and quantified by NanoDrop™ One^C^. Second, bridge base PCR with isoTAT and NaM as well as inherent base's preference PCR with only NaM were performed with 1 ng of the double strand templates (1N, 2N, 3N or UN) and amplified for 36 cycles with all other conditions same to above, besides, the inherent base's preference PCR with only TPT3 were only performed with UN template. Cultured *Escherichia coil* cells with replicated plasmids containing one or three NaM-TPT3 pairs (2 μl) were also used as templates for the above transferred PCR or unnatural base PCR with NaM and TPT3 with changes in the addition of pre-denaturation at 96°C for 3 min but all other conditions same to transferred PCR. The products of the unnatural base, bridge base, and inherent bases PCR were sequenced by Sangon Biotech.

The percent retention of an unnatural base pair (F) was calculated using the raw sequencing data as reported (8,21), it was to use the lack of fluorescent channels for matching UBPs resulting in the signal terminal at the accurate position of UBPs, while the mutations to natural nucleotides resulted in readthrough. The average signal intensities of each channel (A, C, G and T) for defined points (35th–45th positions in the sequence) before (L) and after (R) the unnatural base were determined. The normalized R/l ratio was considered as the percentage of the natural sequences, and retention was calculated as 1 - normalized R/l ratio. So we used a natural template that contained nucleotides that were different from the bridge transformation in the corresponding positions and with the nucleotides behind the unnatural base were all the same, except template 1N with several nucleotides surrounding different from the natural template but did not influence, to determine the R/l′ ratio. R was the average signal intensity of C behind the unnatural base, L′ was the single transferred signal C of bridge base PCR product mixed with natural template PCR products in different fractions. PCR procedure with a natural template was the same as that of bridge base PCR without the addition of unnatural bases. The products of 1N or 3N template by transferred PCR and of natural template were purified with a QIAquick Gel Extraction Kit (QIAGEN) and quantified by NanoDrop™ One^C^. The mixtures containing 100%, 90%, 80%, 70%, 60%, 50%, 40%, 30%, 20% and 0 of bridge base transferred products were prepared and sequenced. The R/l′ ratio over the percentage of bridge base transferred products was plotted and fitted by linear regression (Supplementary Figure S19). Representative sequencing data for 1N and 3N mixtures were shown in Supplementary Figures S17 and S18. The data are averages and standard deviations of three independent determinations.

### Deep sequencing assay

Deep sequencing assays were performed by Sangon Biotech., Briefly, the products obtained from bridge base PCR with isoTAT and NaM or inherent base's preference PCR with only NaM were purified with a QIAquick Gel Extraction Kit (QIAGEN) according to the manufacturer's methods. Then compatible primers for Illumina bridge PCR were ligated to our DNAs using 2× Hieff® Robust PCR Master Mix (Yeasen, 10105ES03), and the samples were enriched by Hieff NGS™ DNA Selection Beads (Yeasen, 12601ES56) and sequenced with a Miseq Reagent Kit 3. The raw data were also supplied in supporting material.

### Growth and *in vivo* replication assay

This was performed according to previous reports (2,25). Briefly, the plasmid containing *Pt*NTT2 reported in the literature was synthesized by GenScript (27). Sequences containing one or three NaM-TPT3 pairs were shown in sequences used in this study. Then they were amplified and assembled into pBLUE-T plasmids. Finally, the two plasmids were transformed into BL 21 (DE3) electrocompetent cells. And cells were cultured with 2 × YT media (200 μl, casein peptone 16 g/l, yeast extract 10 g/l, NaCl 5 g/l), 5 μg/ml chloramphenicol, 100 μg/ml ampicillin, 50 mM KPi) with unnatural bases dNaMTP and dTPT3TP at the concentration of 125 μM. The growth of bacteria (OD_600_) was monitored by NanoDrop™ One^C^ with a cuvette of 2 mm optical path to reach an OD_600_ range at 0.6–2 and then used for PCR assay. The data are averages and standard deviations of three independent determinations.

### Labeling dU lesion in DNA with unnatural nucleotide

KRAS-1U-F or 2U-F and KRAS-R or 134–2U-F and 134–2U-R (0.2 μM in 50 μl) were annealed to form double-stranded DNA in ABclonal buffer B. UDG (1 U) were added to the reaction mixture and incubated at 37°C for 30 min. Then APE1 (10 U) was added to the reaction mixture, incubated at 37°C for 1 h, and heated at 95°C for 10 min. Next, dTPT3TP^biotin^ (30 μM) and Kf (exo^−^) DNA polymerase (7 U) were added to the reaction mixture to react for 1 h at 37°C, and heated to 95°C to terminate the reaction. Finally, T4-DNA ligase (200 U), dimethylsulphoxide (10% (v/v)), and ATP (0.2 mM) were added to the reaction system and incubated at 25°C for 1 h. The reaction steps above were monitored by 20% denaturing PAGE gel.

### Isolation of labeled DNA and bridge PCR

The final reaction solution above (KRAS-1U or 134–2U) was used as the template for further PCR assays. First, the labeled DNA was further labeled and amplified via PCR according to the method reported(2,27), with some modifications: templates (the final reaction solution, 1 μl), dNTPs (400 μM), dTPT3TP^biotin^ (20 μM), dNaMTP (50 μM), MgSO_4_ (2.2 mM), primers (400 nM each) OneTaq DNA Polymerases (0.018 U/μl), and DeepVent DNA polymerase (0.007 U/μl) in 1 × reaction buffer (a total of 25 μl), under the thermocycling conditions: 20 × (96°C, 30 s; 50°C, 10 s; 68°C, 4 min) with a final extension for 68°C, 5 min. The products (5 μl) were incubated with streptavidin (1 μg, Solarbio) for 30 min at 37°C. Then samples were mixed with loading dye and separated by a 6% (134-2U) or 10% (KRAS-1U) non-denaturant polyacrylamide gel electrophoresis. The shift strips were eluted by shaking and soaking at 37°C for 2 h, quantified by NanoDrop™ One^C^, and used as templates for bridge base PCR (0.5–2 ng per sample). Bridge PCR was performed as described above, and inherent dU’s preference PCR as control was also performed with the same procedure as that of bridge PCR.

### Cell growth and preparation of plasmid DNA

Damages of Apurinic and apyrimidinic (AP) sites were induced according to the previous report with some changes (28). Briefly, pUC-19 plasmids were transformed into *E. coli* DH5α cells, a single colony was grown in 2 ml LB medium overnight at 37°C, and then 10 μl culture was diluted with fresh LB medium and incubated at 37°C, 230 rpm until OD_600_ = 1 for H_2_O_2_ treatment. H_2_O_2_ was added to the culture at a final concentration of 1 mM to generate AP sites. After sitting at ambient temperature for 30 min, cells were harvested by centrifuge at ambient temperature and plasmid DNA was extracted by the plasmid isolation kit following the manufacturer's protocol (OMEGA). Plasmids from cells without H_2_O_2_ treatment were used as a control.

### Data analysis

Deep sequencing data for AP sites were analyzed by mutation levels of the nucleotides in each site. As each of the four natural bases might be damaged to AP sites, and then transformed into different natural bases after NaM-TPT3 incorporation by replacement PCR. If one nucleotide was damaged and an AP site formed, then TPT3 would be incorporated into the sites, and finally, C or A at the site would be detected by the replacement PCR with bridge base or only NaM respectively. For G and T, the mutation levels of A and C could be calculated. For A and C, only the mutation levels of C or A could be calculated, as they were one of the nucleotides being transformed. The mutation ratios of controlled PCR were used as input to eliminate the background.

### Binding analyses

The binding affinities of aptamers with TPT3 or natural bases to IFN-γ were measured by SPR (Reichert2, Reichert). Briefly, 5’-biotin labeled aptamers were diluted to 50 nM in 1× PBS, denatured at 90°C for 1 min, cooled down slowly to 25°C, placed on ice for more than 5 min, supplemented with Nonidet *P*-40 at a 0.005% (wt/vol) final concentration, and immobilized on a Sensor chip SA by injecting the DNA solution at a flow rate of 10 μl min^−1^ in 1× binding buffer (immobilization level: ∼200 response units). Concentration series of IFN-γ were injected at a flow rate of 25 μl min^−1^ for 180 s, and the dissociation was monitored for 300 s in 1 × running buffer (1 × PBS with 0.05% wt/vol Nonidet *P*-40 and 50 mM NaCl). After each injection, the sensor surface was regenerated with 25-μl injections of 5 mM NaOH for 60 s, and the following refolding of DNA was accomplished by 1 × running buffer for 10 min. The control sensorgrams of a reference cell lacking immobilized DNA fragments on the sensor surface with the buffer injection were subtracted from each sensorgram of the aptamers from a measuring cell, and the data were fitted with a 1:1 binding model.

## RESULTS

### Design of the bridge base isoTAT for pairing with both NaM and G.

To develop a bridge base for TPT3-NaM UBP, we need to design a structural skeleton that can efficiently pair with one of TPT3/NaM bases, and simultaneously effectively pair with one of the natural A/T/G/C bases. TPT3 represents an optimal base that has been proven to pair well with a panel of NaM-type unnatural bases and shows tolerance for the structural changes to keep its pairing capacity (8,20,25). Based on the properties, we reason that a moderate modification of TPT3 base may generate a new base that will keep efficiently pairing with the NaM base in the successful recognition model of TPT3-NaM pair, and simultaneously can enhance its pairing capacity with one of the natural bases. Furthermore, we expect that the incorporation of a hydrogen-bonding donor/acceptor will be a choice to alter the pairing capacity of TPT3 with natural bases, especially the addition of a hydrogen-bonding acceptor on the inner side of thienyl motif on TPT3, which is expected to form an effective hydrogen bond with N-1 of G base (G/isoTAT in Figure 1A). Finally, we suspect that the aza-modification analogue of TPT3, isoTAT, will not pair with the other three natural bases of A/T/C due to either steric hindrance or hydrogen bond repelling (Figure 1B). Thus isoTAT can be used as a bridge-base candidate.

**Figure 2.**
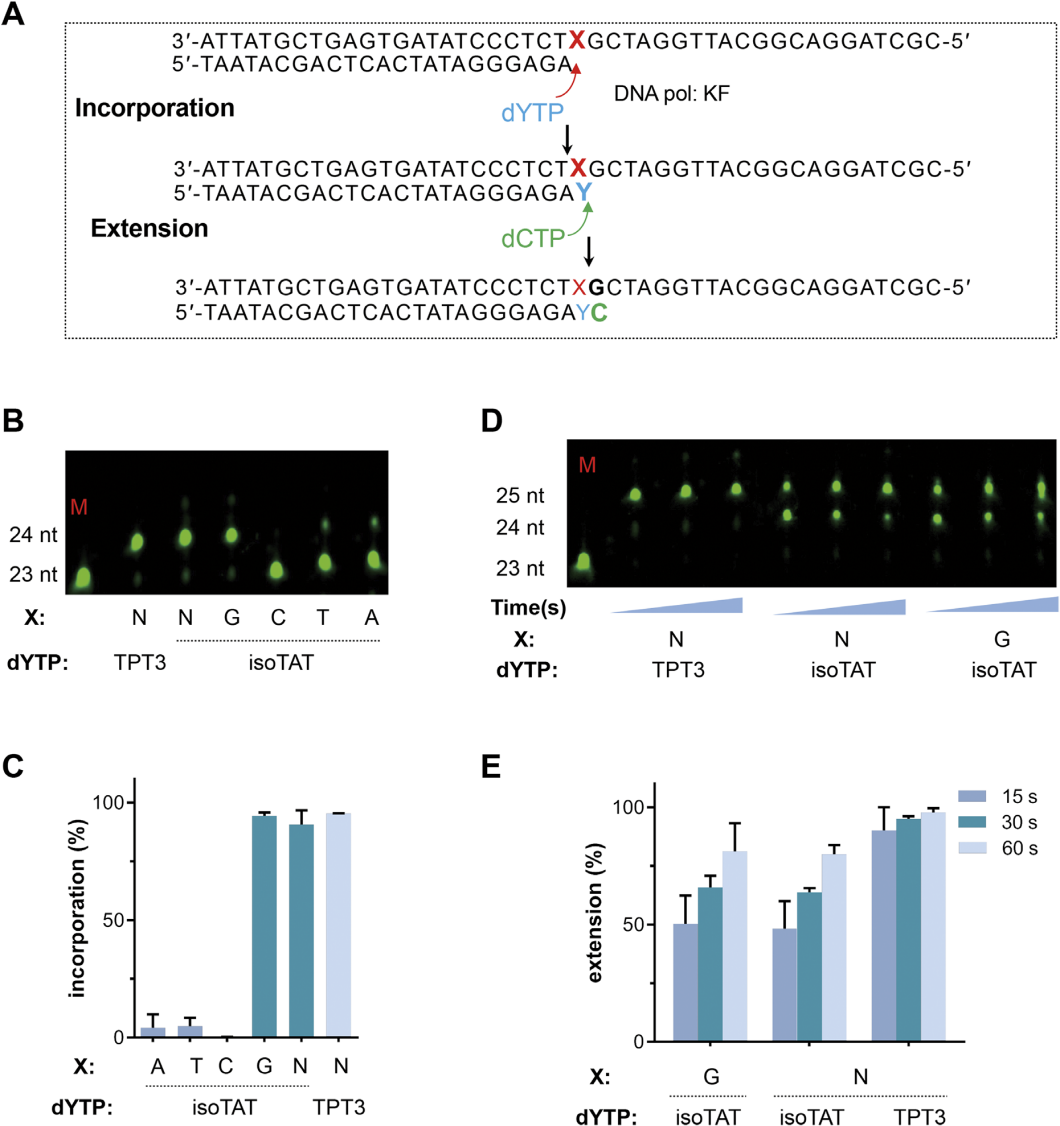
Kinetics analysis for isoTAT. (**A**) Scheme of Kf-mediated incorporation and extension assays for isoTAT; (**B**) representative gel for isoTAT incorporation using a pre-steady-state assay; (**C**) incorporation level; (**D**) representative gel for isoTAT extension by a pre-steady-state assay; (**E**) time-coursing extension level; X represents the corresponding nucleotides used in templates. Y represents the corresponding nucleotides used for kinetics analysis. M: 23 nt primer. N: NaM. Incorporation assay condition: 0.5 μM dYTP, 15 s. Extension assay condition: 0.5 μM dYTP, 15 s and then 2 μM dCTP, another 15, 30 or 60 s.

To investigate the pairing capacity of isoTAT, we used presteady-state assays with 45-mer template and 23-mer primer (Figure 2A). Gratifyingly, co-incubation of the templates, primers, and disoTATs with Klenow fragment of *E. coli* DNA polymerase I (Kf (exo-)) for 15 s, resulted in efficient incorporations of isoTAT opposite NaM in the template with a ∼90% yield and opposite G in the template with a ∼94% yield (Figure 2B and C). Furthermore, we measured the pairing ability of isoTAT with A, T and C bases with the same assays, and less than 10% incorporation yields could be obtained (Figure 2B and C). These data indicate a high selectivity of isoTAT base to recognize both NaM and G, while much less effective to recognize A, T and C.

Steady-state kinetic assays were further performed to characterize the efficiencies ((second-order rate constant, *K*_m_/*V*_max_) (Figure 2A). The insertion efficiencies of disoTAT opposite dNaM or dG were 7.68 × 10^7^ and 2.14 × 10^8^ respectively, which was comparable to that of NaM-TPT3 at 3.36 × 10^9^. Moreover, no detectable reactions could be measured for A-isoTAT, T-isoTAT and C-isoTAT under the same conditions (Table 1 and Supplementary Figure S1). Usually, over two orders of magnitude for *K*_m_/*V*_max_ values would lead to the exclusive pairing capacity for UBPs (29). The remarkable insertion efficiencies of disoTAT opposite dNaM and dG provided another evidence that isoTAT could pair both certain unnatural base NaM and natural base G. Because a high strand extension rate of UBPs was key for their replications to give full-length products, the extension abilities of the primer after isoTAT incorporated were also investigated. The incorporated products of NaM-isoTAT and G-isoTAT respectively with the next complementary dCTP were incubated, time-coursing extension reactions were detected within 60 s to give ∼80% yields of extended products, which were just slightly lower than the yield of NaM-TPT3 UBP itself (Figure 2D and E). In addition, we also found that isoTAT could extend to the next G with concentration-response and time-coursing after incorporating opposite NaM or the first G (Supplementary Figure S2). With the NaMG template, the extension yield of isoTAT: G (25 nt) arrived at 92.5% with 8 μM disoTATTP after 5 min; alternatively, the extension yield of isoTAT: G (25 nt) could also arrive at 82.6% with 2 μM disoTAT-TP after 10 min. With the GG template, the extension yield of isoTAT: G (25 nt) arrived at 95.3% with 8 μM disoTATTP after 5 min. These suggest that isoTATs can be consecutively inserted in the DNA. Taken together, these kinetic data indicate isoTAT may be used as a bridge base.

**Table 1.** Steady-state kinetic assays

Template	dXTP	*V*_max_ (%·min^−1^)	*K*_m_ (μM)	*V*_max_/*K*_m_ (%·min^−1^·M^−1^)
NaM	isoTAT	20.97±1.61	0.273±0.073	7.68×10^7^
G	isoTAT	17.93±0.64	0.084±0.014	2.14×10^8^
T	isoTAT	n.d^a^	n.d^a^	n.d^a^
C	isoTAT	n.d^a^	n.d^a^	n.d^a^
A	isoTAT	nd^a^	nd^a^	nd^a^
NaM	A			7.10×10^5b^
NaM	TPT3	23.38±1.47	0.007±0.002	3.36×10^9^
G	CTP	23.33±1.73	0.011±0.004	2.1×10^9^
T	ATP	8.03±0.78	0.006±0.002	1.43×10^9^

nd^a^ represents the kinetic constants that are lower than can be detected. b represents the kinetic constant that is from the literature (24).

### Dual location of multiple NaM-TPT3 pairs.

**Figure 3.**
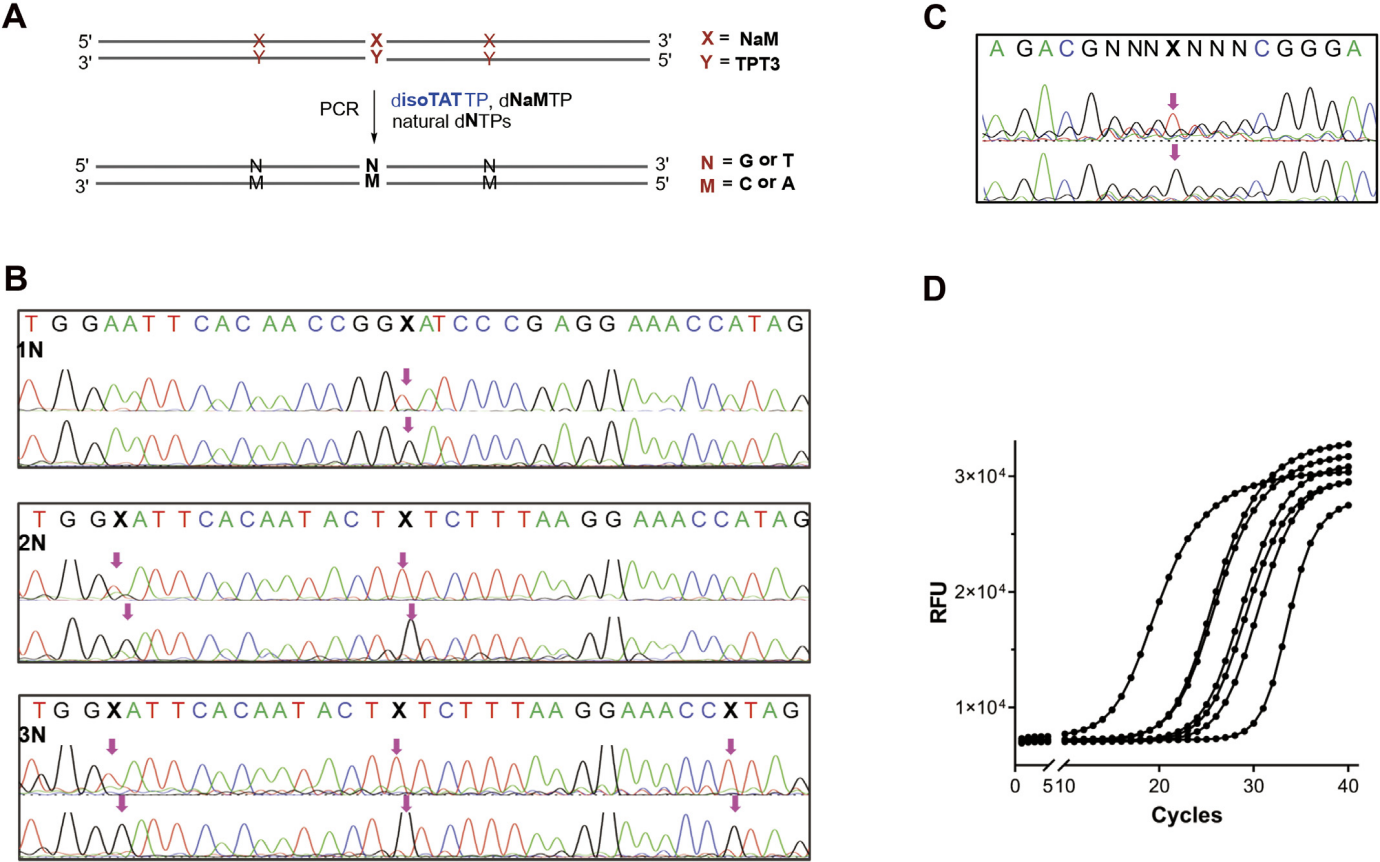
Conversion validation of TPT3-NaM to natural base pairs using Sanger sequencing. (**A**) Workflow of transferred PCR. X and Y represent templates with or without TPT3-NaM. (**B**) Sanger sequencing of templates containing 1, 2 or 3 NaM-TPT3 base pairs after transferred PCR. 1N, 2N and 3N represent that PCR is performed with templates containing 1, 2 or 3 NaM-TPT3 base pairs. (**C**) Sanger sequencing of UN templates. PCR results with isoTAT and NaM showed the transformation of NaM-TPT3 to G–C (below), and PCR results with only NaM showed the transformation of NaM-TPT3 to T–A (above). Only the sense stands with NaM are shown. A: green; T: red; G: black; C: blue; the red arrowheads indicate the corresponding positions of NaM in the templates. (**D**) 40 cycle qPCR amplification of (from left to right) 400, 40, 4, 0.4, 0.04 and 0.004 pg of template 1N. Amplification without a template as a negative control was the rightmost curve signal resulting from primer-dimers.

Twice replacement PCR strategy has been used by Benner's team and Hirao's team for sequencing their P-Z and Ds-Px UBPs (7,30). This means that unnatural base pairs can be converted into different natural base pairs with two varied PCR conditions. To this end, we expected the presence of disoTATTP in the PCR buffer would be the optimal choice to induce the transformation of TPT3-NaM to natural C–G pairs during the amplification. On the other hand, the kinetic data have suggested an A-base's preference of NaM in some sequence contexts (24,26), and our kinetic data also showed that NaM could pair with A but not the other natural nucleotides (Supplementary Figure S3 and Table 1). We therefore designed PCR assays using disoTATTP/dNaMTP or only dNaMTP with natural dNTPs and expected the potential of using disoTATTP and the NaM own bases’ preferences to monitor multiple TPT3-NaM pairs (Figure 3A). Double-stranded DNA templates 1N, 2N, 3N and UN containing one, two, three, or one NaM-TPT3 (with three random nucleotides upstream and downstream), were chosen respectively. As shown in Figure 3B and C and Supplementary Figures S4 and S5, the results indicated that the NaM-TPT3 pairs surrounded by specific sequences or random nucleotides were all mainly converted to G–C with disoTATTP/dNaMTP and natural dNTPs. And it was also confirmed that the NaM-TPT3 pairs were all mainly converted to T–A with only dNaMTP and natural dNTPs. The results demonstrated that the combination of our bridge base and the inherent base's preference of NaM, through twice PCR assays and the followed Sanger-sequencing signals’ comparison, could offer an effective and concise method for determinations of multiple TPT3-NaM UBPs with known or unknown loci. Moreover, the effects of quantity of templates were also investigated via qPCR amplification using the 1N template under the two PCR conditions. After 40 cycles, we found that the amplification of 1N remained efficient and accurate even with only 0.004 pg, and PCR with only NaM showed higher amplification efficiency (Figures 3D and S6).

To further evaluate the practicality of this method, PCR amplification efficiency with 1N template was analyzed using a gel-based assay by band brightness ratio (gray ratio). The result showed their considerable replication efficiency of dTPT3TP/dNaMTP, only dNaM or disoTATTP/dNaMTP with a gray ratio of 1.0:1.2:0.8 (Supplementary Figure S7). Moreover, raw sequencing signals of PCR products were also analyzed. They showed no cut-down in the presence of disoTATTP/dNaMTP or only NaM (Supplementary Figure S8), which also proved that the unnatural bases were converted to natural bases. Furthermore, we analyzed the signals of all other natural bases before and behind the UBP, aiming to find possible disadvantageous effects of the bridge base isoTAT on the replications of natural base pairs. Gratifyingly, no detectable mutations of natural bases in all sequencing signals except the UBP site (Supplementary Figure S8). These might be probably due to the higher pairing efficiency of natural base pairs, NaM-isoTAT and isoTAT-G (*K*_m_/*V*_max_ values of both A–T and C–G are over 10^9^) as well as the undetectable pairing efficiency between isoTAT and other natural bases. Finally, dTPT3TP and natural dNTPs were used for PCR assays to explore whether TPT3 had its inherent preference in the absence of NaM, only messy signals were obtained at the predetermined position of TPT3-NaM UBP in template UN (Supplementary Figure S9), suggesting that TPT3 could not be used to improve the dual location of TPT3-NaM pairs. These experiments identified two transferred PCR that can efficiently and specifically transfer NaM-TPT3 to different natural pairs, thus offering a promising TPT3-NaM location method.

### Sequence-dependence analysis.

**Figure 4.**
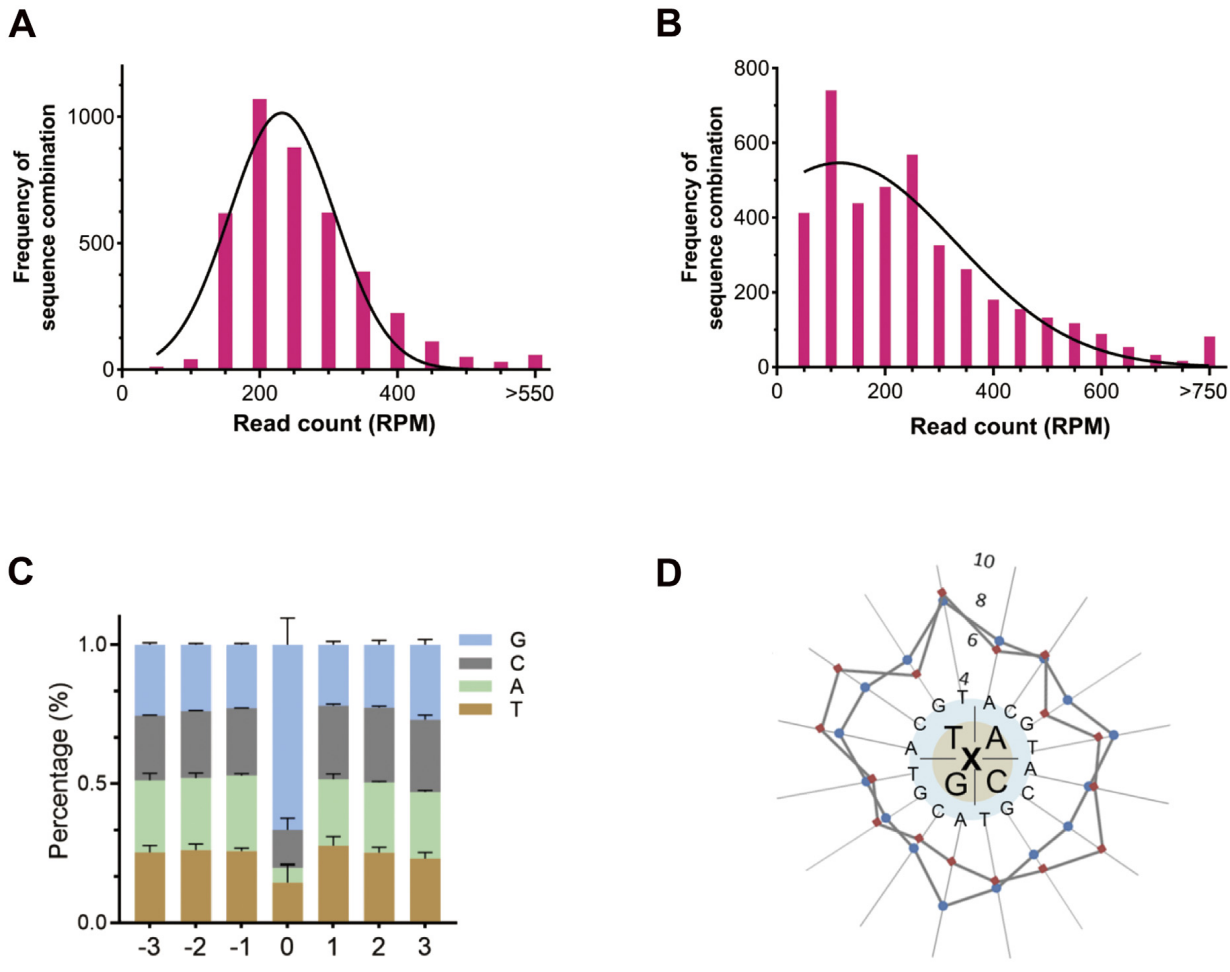
Conversion validation of TPT3-NaM to natural base pairs using Deep sequencing medicated by isoTAT and NaM or only NaM. (**A**) The frequency of sequence combinations mediated by isoTAT and NaM. (**B**) The frequency of sequence combinations mediated by only NaM. (**C**) The distribution frequency of natural bases in each random locus and NaM locus medicated by isoTAT and NaM. (**D**) The distribution frequency of dinucleotides in the bridge-base transformation. Red squares represent dinucleotides downstream, blue circulars represent dinucleotides upstream. The coordinate unit is 10 000.

Because the sequence dependence has shown a detrimental effect on both *in vitro* and *in vivo* replication of hydrophobic UBPs (27,31), we considered that a detailed investigation of the sequence dependence of the current transferred PCRs should be necessary for promoting its general applications. So we ran the deep sequencing on the PCR products of UN template with the nucleotide conditions of Group-I (disoTATTP, dNaM, and natural dNTPs) and Group-II (dNaM and natural dNTPs) respectively. Samples with triplicate were normalized to 1 × 10^6^ read counts and further analyzed. For the bridge-base transformation samples, a standard bell-shape frequency distribution around the median number (244) indicated that there were no significant deviation differences for the detected sequence combinations (Figure 4A). In contrast, the frequency distribution of NaM-mediated transformation samples demonstrated a less regular bell shape with some sequence combinations bearing less than 100 reads or more than 750 reads, suggesting a few special sequence contexts bearing abnormalized preferences (Figure 4B). However, for both two transformations, we could obtain 4096 kinds of readthrough sequences, which covered all the possible sequences of the random arrangements. The total 100% read-through ratios guaranteed the generality of the methods for applications in various DNA technologies.

The detailed effects of nearby bases on the UBP’s transformations could also be analyzed by the deep-sequencing data. The single base distribution from the before and behind three positions for the bridge-base transformation was calculated first. As shown in Figure 4C, no base preferences could be found for the detected six natural bases’ positions (each of them was about 25%), and all over 66% G signals indicated the effective transformation of NaM to G. With the same analysis method, all over 60% T signals indicated the effective transformation to T under the NaM-mediated transformation and base's preferences could be found for the three position downstream (Supplementary Figure S10). Secondly, we evaluated the effects of dinucleotides in the bridge-base transformation. As shown in Figure 4D, for all the 32 combinations upstream or downstream, no special dinucleotide combinations showed dramatic preferences, with the number of reads ranging from about 40 000 to 80 000. For the NaM-mediated transformation, the read counts of combinations upstream showed no dramatic preferences and were similar to those of bridge-base transformation, while the read counts of combinations downstream showed dramatic preferences, TT and TA combinations showed high frequency with a number over 100000, and AA, AG, GA, and GT showed low frequency with the number <40 000 (Supplementary Figure S11). Such preferences could give a reasonable explanation for the less regular bell-shape distribution shown in Figure 4B and S10.

**Figure 5.**
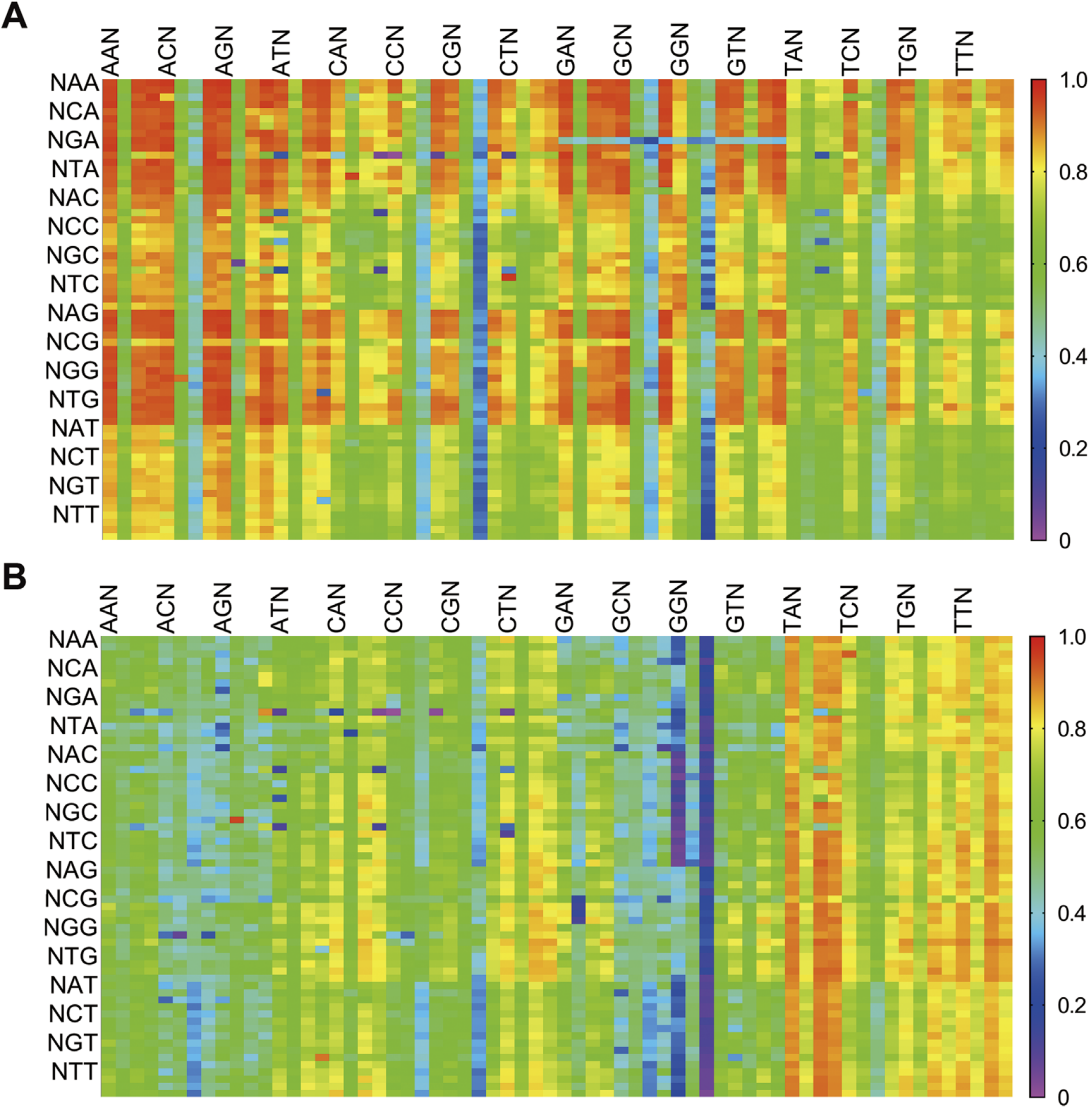
Percentage of NaM transferred to specific natural bases in each sequence context. (**A**) Proportions of NaM to G transformations medicated by PCR with isoTAT and NaM in each sequence context. (B) Proportions of NaM to T transformations medicated by PCR with only NaM in each sequence context. The third bases upstream (left) and downstream (up) of the unnatural base are simplified as ‘N’ and arranged in the order of A, C, G and T in each dinucleotide combination.

Finally, we analyzed the effects of triple bases on the UBP’s transformations, the results were shown in Figures 5A, B, and S12, 13. For all the 4096 combinations of bridge-base transformation, TPT3-NaM were mainly transferred to C–G in about 95% of the combinations, except sequence contents with CGG and GGG downstream and a few specific sequence combinations were transformed to G–C, A–T and T–A (Figure 5A and S12). For the 4096 combinations of NaM-mediated, TPT3-NaM were mainly transferred to A–T in about 91% of the combinations, except part of sequence contexts with GGA, all sequence contexts with GGG downstream, and a few specific sequence combinations were transformed to G–C, A–T and T–A (Figures 5B and S13). Although variable transformation in some sequence contexts mediated by bridge-base or NaM were also different, about 96% of the total sequence combinations could complete the dual location of NaM-TPT3 pair. There were 4% triple-base sequence contexts that did not support the dual location of NaM-TPT3 pair possibly because of their unique combinations (Supplementary Table 1). Taken together, these results indicate our dual location method possesses the characteristic of high read-through ratios and low sequence-dependent properties.

### Tracing the *in vivo* replication of multiple NaM-TPT3 pairs in the plasmids.

**Figure 6.**
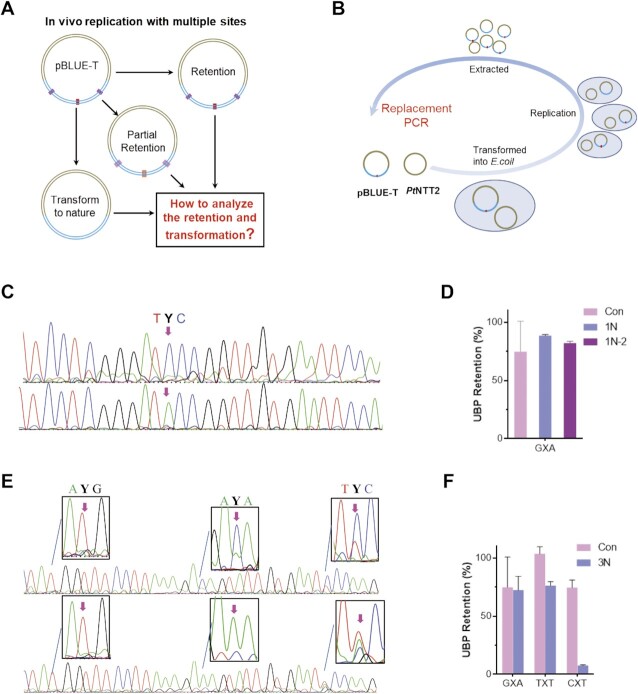
Retention testing by bridge base with standard curves. (**A**) Outline for multiple unnatural bases retention. (**B**) Workflow for the *in vivo* replication and identification. (**C**) Representative Sanger sequencing data medicated by isoTAT and NaM or only NaM with plasmids containing one UBPs. The antisense strand with TPT3 is shown here. The red arrowheads indicate the corresponding positions of TPT3 in the templates. (**D**) Retention analysis of 1N template. Con (D and F): retention data from the previous report in the same sequence context(27); 1N: retention data from the standard curve; 1N-2: retention data from signal attenuation. (**E**) Representative Sanger sequencing data medicated by isoTAT and NaM or only NaM in plasmids containing three UBPs. The antisense strand with TPT3 is shown here. (F) Retention analysis of 3N template. 3N: Retention data from our standard curves.

There were increasing requirements for the incorporation of multiple TPT3-NaM UBPs or UBPs with non-default loci, for example *in vivo* replications in SSO to insert more ncAAs and SELEX. So the tool for tracing multiple UBPs or UBPs with non-default loci is urgently needed, which is limited by existing methods such as the Sanger-terminal method and biotin-shift assays (Figure 6A). And the emerging data have shown that some sequence contexts are less acceptable for *in vivo* replications (more harsh sequence requirements than that of *in vitro* replications (27), thus needing to examine thoroughly the mutation products of UBPs-bearing plasmid after their doublings. Here we chose the plasmid that contained one or three UBPs with different neighboring bases for the proof-of-concept retention/mutation analysis (Figure 6B).

The plasmids were prepared, transfected based on the literature methods^2^, and extracted until OD_600_ reach a range of 0.6–2 (1N: 1.7; 3N: 0.7). The extracted plasmids were amplified via replacement PCR of 36 cycles with bridge base and NaM or only NaM. The dual location method allows us to analyze the detailed changes of the UBPs’ mutations *in vivo*. The results showed that NaM was almost all transformed into G or T by bridge base or only NaM PCR in the plasmid containing one UBP with the three base combination GXA (X = NaM), respectively (Figures 6C and S14). This might be due to the high retention of the UBP as seen in Supplementary Figure S15. Intriguingly, NaM bases in the plasmid containing three UBPs were converted into a mixture of A and G or A and T in the triple base combination of GXA, and into a mixture of T and G or T in the triple base combination of TXT, indicating a certain degree of retention as well as mutations of NaM to A in GXA and T in TXT combination. While in CXT base combination, a nearly complete mutation of NaM was to A (Figure 6E, Supplementary Figure S16). Our method directly gave the information of UBPs’ mutations of DNA bearing multiple UBPs replicated in SSO, we expected it would be applied to other applications to identify multiple UBPs at unknown sites.

Beyond the qualitative analysis of base distribution, we attempted the possibility to employ the bridge base for quantitative analysis of contents of retained bases on the positions of UBPs after *in vivo* replications by the standard curve methods. 1N and 3N templates were amplified by bridge base to transform NaM-TPT3 to G–C, then the products were mixed with natural sequences in different proportions and sequenced (Supplementary Figures S17 and S18). The signal ratio of C on the antisense strand was used for generating the standard curves (Supplementary Figure S19). First, we tested the plasmid-A sample bearing one TPT3-NaM UBP with the three-base combination GXA. With the standard curve built for this sequence, the UPB retention was calculated as 88%, which was comparable to 82% that was measured by signal attenuation assays with NaM-TPT3 as some unnatural bases would be lost during PCR (Figure 6D and Supplementary Figures S15 and S20). Then the plasmid-B samples bearing three TPT3-NaM UBPs were analyzed. Interestingly, the retention or mutation ratio for the UBPs with different neighboring bases in the same strand changes obviously. Taking the antisense strand for calculation, for the GXA base combination, 72% retention could be obtained. In contrast, 76% retention of UBP was obtained for the TXT base combination, and only 7.5% retention of UBP for the CXT base combination (Figure 6F and S21, 22). Finally, sequences were completely read-through with no detectable mutations of natural bases found by our method, indicating the desirable bioorthogonality of NaM-TPT3 for applications *in vivo* replications (Figure 6C, E, and Supplementary Figure S23). In all, we show that our dual location method is a powerful tool for tracing multiple UBPs or UBPs with non-default loci.

Additionally, we also compared our data with the reported retention ratios for which each triple base combination was evaluated individually (27). The triple base combinations measured by our bridge base method showed that TXT had good retention efficiency, GXA showed moderate tolerance, and CXT was the most affected (Figure 6D and F). This was in agreement with the previous report, although the report also showed that the retention efficiencies of UBPs were influenced by plasmid types, triple base combinations, and strains of *E. coil* (27). Apart from the copies of plasmid bearing UBPs that were different (OD_600_, 1N: 1.7; 3N: 0.7), taking the retention efficiency of GXA in two plasmids into consideration (Figure 6D and F), we speculated that more sequence contexts beyond the neighboring bases and the close arrangement of multiple UBPs might also play a role in controlling the retention of UBPs during their *in vivo* replication. To test the hypothesis, kf-mediated kinetic assays have been applied to reflect the possible sequence dependence of TPT3-NaM UBPs that frequently occurs in *in vivo* replications (26). We synthesized nine flanking sequences with NaM and three flanking sequences with TPT3 in templates to explore the influence of the flanking sequence, including templates with three near-flanking nucleotides the same as that replicated *in vivo*. The mispairing abilities are thought to be an important factor that leads to UBP loss (27). We found that the sequences farther from the adjacent three bases could slightly affect the mispairing capacity of UBPs (Supplementary Figure S24); the adjacent three bases on the upstream and downstream of UBPs had more powerful effects on their mispairing tendency (Supplementary Figure S25). To further explore whether the adjacent three bases on the upstream and downstream of UBP affected the mispairing level, kinetic analysis using templates with three near-flanking nucleotides the same as that replicated *in vivo* was also performed. Mispairing levels of NaM and TPT3 in one site were added together as total mispairing rates. We found that the TXT, GXA, and CXT on the same sequence context also showed different mispairing capacities, in the order CXT > GXA > TXT (Supplementary Figure S26), which was consistent with the fidelity of *in vivo* replication of the three combinations (TXT > GXA > CXT). These results suggest that more flanking sequences should be taken into account for high-fidelity replication *in vivo*.

### Sequencing multiple-site DNA lesions and aptamer containing multiple TPT3.

**Figure 7.**
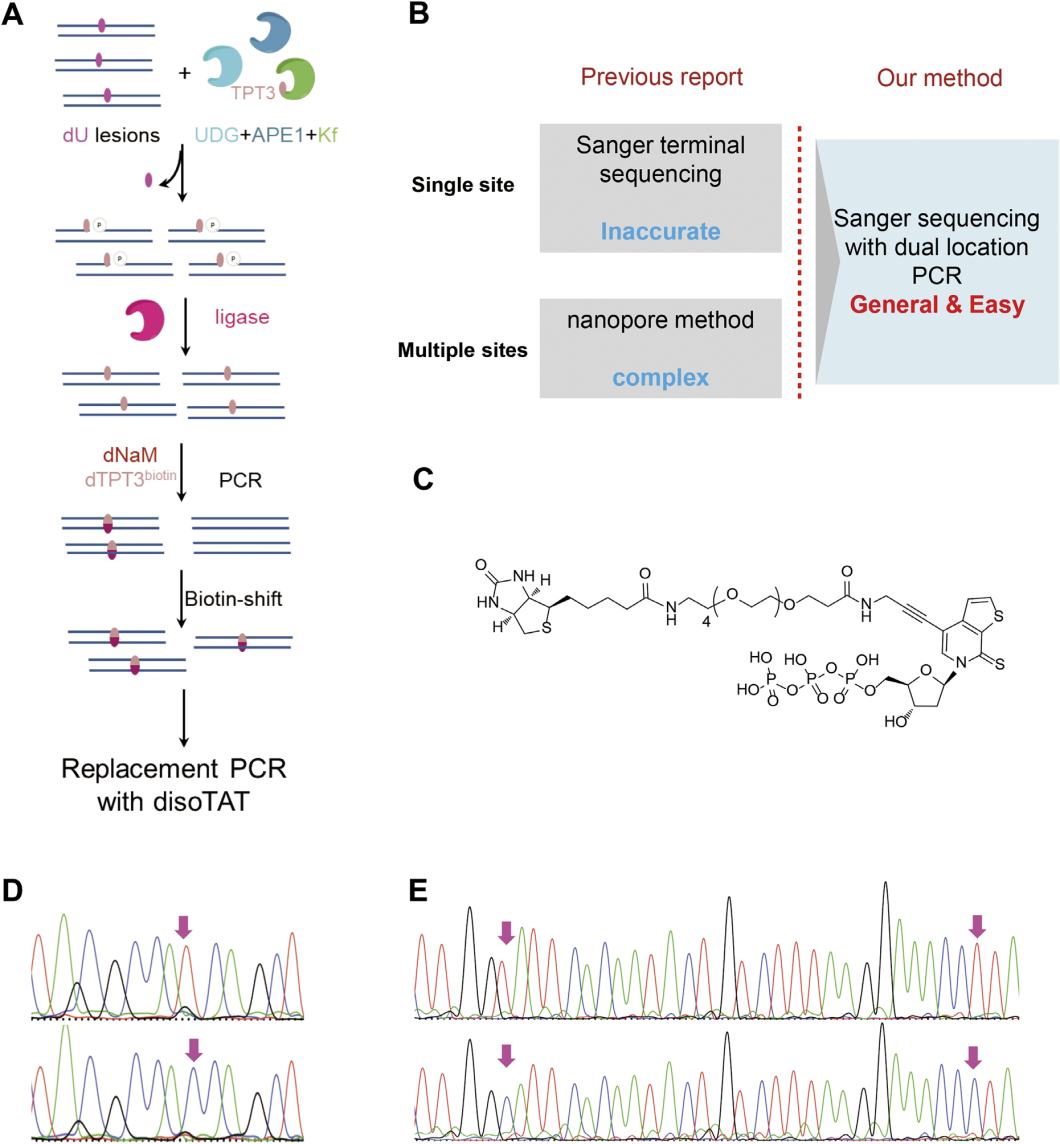
A new tool for accurately identifying multiple-site DNA lesions with bridge-base method. (**A**) The brief scheme for identifying two dU lesions by bridge-base method. (**B**) Overview of the outstanding features of our method. The method previously reported was from the literature(22). (**C**) The chemical structure of TPT3TP^biotin^. (**D**) Sanger sequencing results after PCR amplification of a single strand DNA template containing a dU locus with natural dNTPs or a double strand DNA template containing a NaM-TPT3^biotin^ pair with isoTAT and NaM. (**E**) Sanger sequencing results after PCR amplification of a single strand DNA template containing two dU loci with natural dNTPs or a double strand DNA template containing two NaM-TPT3^biotin^ pairs with isoTAT and NaM. The double strand DNA templates containing one or two NaM-TPT3^biotin^ pairs were transferred and purified from dU as seen in (A). Only the sense stands with dU are shown. Red arrowheads indicate the dU loci or transferred dU loci.

The exclusive pairing capacity of UBPs enables their successful applications in a variety of biotechnologies, among which Burrow and coworkers have used 5SICS-NaM as the third pair for the identification of DNA lesions such as abasic site (AP site), dU and dOG (22). Lesion sites were replaced by 5SICS-NaM pair via the base excision repair process and PCR amplification. Then Sanger sequencing was performed to determine the position of a single lesion site depending on the terminal signal. And α-hemolysin nanopore method was used to detect more than one lesion site via identifying the substituted NaM with post modified reporting group. Here we provided a proof-of-concept for improving the identification of such multiple lesions by our bridge bases (Figure 7A). As lesion excision and unnatural nucleotide insertion were similar via the base excision repair process, dU was used to represent varied lesions. More than one dU site in the strand that was marked by TPT3-NaM could be read out after running the twice PCR assays without the involvement of specialized instruments (Figure 7B).

We prepared the template DNA (KRAS-1U, 2U and 134–2U) bearing dU lesions according to the literature (22), and then validated the performance of our method. Briefly, uracil-DNA glycosylase (UDG) was employed to yield AP sites, and apurinic/apyrimidinic Endonuclease 1 (APE1) was used to yield gap sites without the sugar fragment. Then Kf (exo^−^) enzyme was used to insert the dTPT3TP^biotin^ (Figure 7C). In order to seal the marker nucleotide at the lesion site, T4-DNA ligase was added to the reaction mixture. The procedures for gap-formation reaction and ligation reaction were analyzed by denaturing PAGE gel (Supplementary Figure S27). Two shorter DNA strands were found after treating the template DNA with APE1, and full-length products could be detected after T4-DNA ligase was added. Then the reaction products were used for PCR amplification with dTPT3TP^biotin^ and NaM, and the PCR products were conveniently enriched by biotin-streptavidin-based strand shift (Supplementary Figure S25).

The enriched strips, expected to contain the TPT3^biotin^-NaM marked DNA lesion, were used as the templates for bridge-base PCR. The single strand DNAs containing one or two dU were also used as templates for PCR amplification to make up the dual location method through the pairing of dU with A, distinguishing signal conversion of bridge-base PCR. With the one-dU-site-containing DNA sample, the dU site replaced with TPT3^biotin^ was transferred to C, while the original dU site was transferred to T (Figure 7D). With the two-dU-site-containing DNA sample, dU sites replaced with TPT3^biotin^ were both transferred to C, while original dU sites were both transferred to T (Figure 7E). The results indicate that our TPT3^biotin^ can be inserted into dU lesion sites and transferred to C by bridge-base PCR, and dU can be identified in this model system.

**Figure 8.**
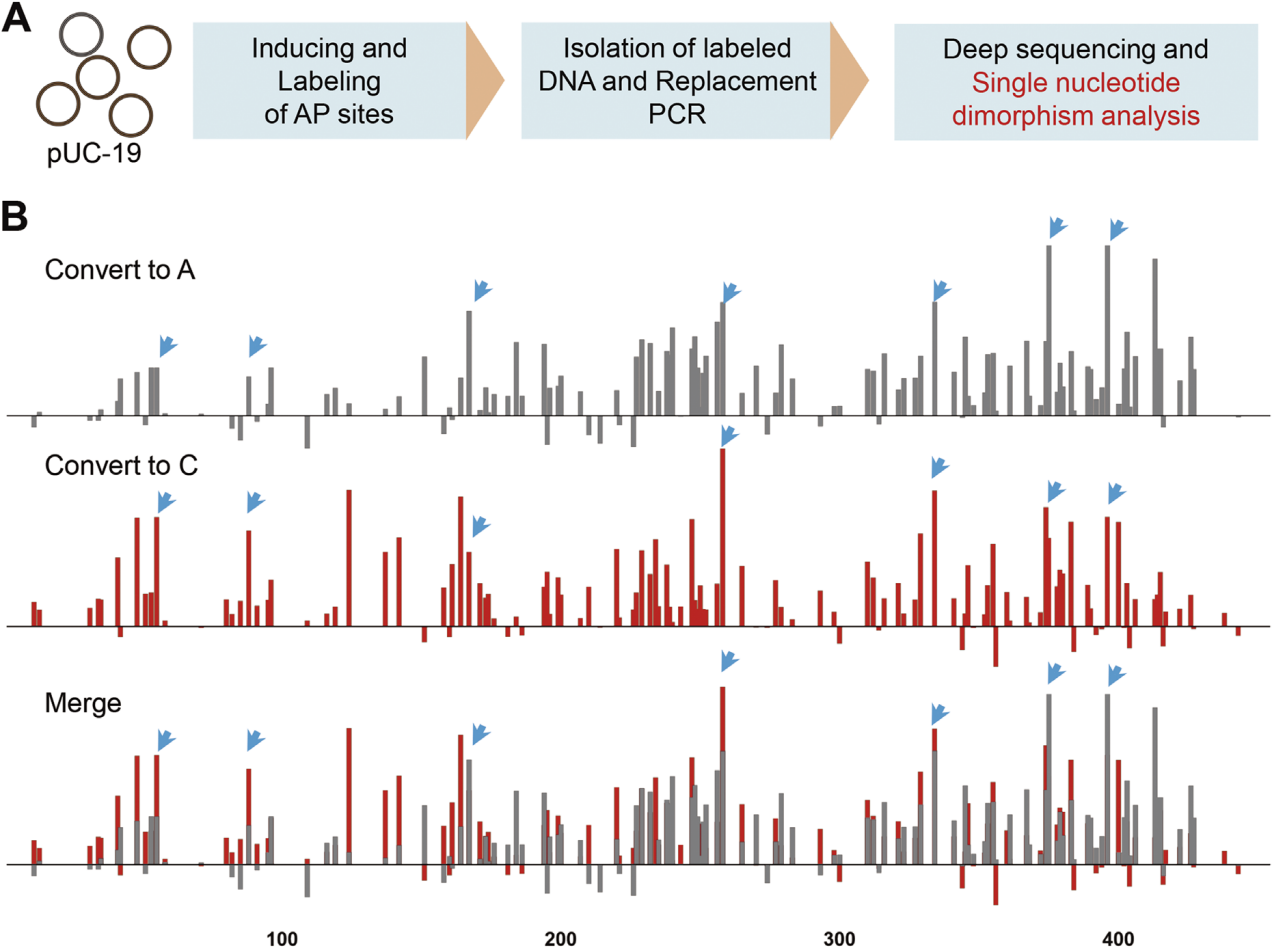
Mutation ratios of G on sense strand from Deep sequencing data of DNA fragments in the plasmid after replacement PCR. (**A**) The workflow of detecting AP sites. (**B**) Mutation ratios of G on the sense strand of DNA fragments in the plasmid after replacement PCR. Blue arrowheads indicate sites increased most with overlap. The distribution of G is also shown.

Furthermore, we applied our method to detect apurinic and apyrimidinic (AP) sites in simple biological samples. It has been reported that AP sites induced by H_2_O_2_ displayed a preference for regions undergoing replication or transcription (28), we attempted to evaluate the corresponding mutation levels of the nucleotides on DNA fragments in replication regions of pUC-19 plasmids. pUC-19 plasmids were transformed into *E. coli* DH5α cells, and damages of AP sites were induced by H_2_O_2_. Labeling AP sites in plasmid DNA with unnatural nucleotide dTPT3TP^biotin^, isolation of labeled DNA, replacement PCR, and Deep sequencing were performed as described above. If one nucleotide was damaged to AP site, it will be transformed to A or C by replacement PCR. For A and C, only the mutation levels of C or A could be calculated respectively, as they were one of the nucleotides being transformed. We found that the mutation ratios of A to C and C to A were both selectively increased in some sites (Supplementary Figure S28). Moreover, we also found that mutation ratios of some G and T sites increased significantly and most sites with increased A or C signals overlapped well especially these increased most, while the mutation ratios of other sites were near the baseline (Figure 8 and Supplementary Figure S29). These indicate that AP sites may be accumulated in some preference regions, and our bridge method can be used to determine DNA lesions such as AP site.

**Figure 9.**
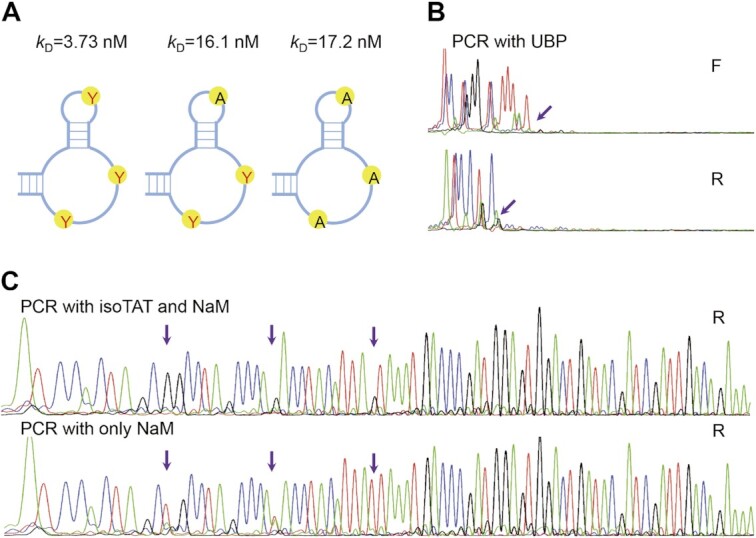
Affinities of the aptamers with TPT3 to IFN-γ measured by SPR and Sanger sequencing of aptamers after replacement PCR. (**A**) Affinities of the aptamers with TPT3 to IFN-γ measured by SPR, Potential structures of these aptamers according to report (11). Y: TPT3. (**B**) PCR results with NaM and TPT3 showed terminated signals after the first UBP. (**C**) PCR results with isoTAT and NaM showed the transformation of NaM-TPT3 to G–C(above), and PCR results with only NaM showed the transformation of NaM-TPT3 to T–A (below). Only the antisense stands with NaM are shown for replacement PCR. A: green; T: red; G: black; C: blue. The purple arrowheads indicate the corresponding positions of NaM in the templates.

Finally, for the SELEX application, one or more UBPs will be needed to arrange in short DNA fragments (11–13). Ichiro Hirao's team has generated aptamers containing multiple Ds-Px pairs with high affinities. Aptamers generated with hydrophobic Ds-Px pair were synthesized (11). The Ds sites were replaced by TPT3 or natural bases, and affinity analysis was performed by SPR. We found that aptamer with two TPT3 showed a similar affinity with that of natural bases aptamer, and aptamer with three TPT3 showed improved affinity than that with natural bases (Supplementary Figure S30 and 9A), which indicated that NaM-TPT3 pair or at least TPT3 could be used in SELEX. The aptamer with three TPT3 was also amplified via PCR with NaMTP/TPT3TP or replacement PCR. We also showed that aptamers with three TPT3 will be terminated after the first UBP without transformation and could be sequenced by our bridge base method without sequence variations (Figure 9B and C). In a word, these results indicate that multiple NaM-TPT3 UBPs that exist in aptamers can be easily detected by our bridge base method, shedding new light on the application of NaM-TPT3 pair in SELEX screening.

## DISCUSSION

In this manuscript, we have designed and synthesized an isoTAT base to facilitate the tracing of TPT3-NaM UBPs for variant research purposes. In particular, we found that the isoTAT bearing similar shapes with TPT3 but possessing additional hydrogen bond acceptor has an intriguing pairing property to pair with NaM and G with almost equal efficiency. For example, in the single-nucleotide insertion experiments, the % incorporations of disoTATTP opposite NaM or G in the templates are 90% and 94% respectively, and the Km/Vmax constants of disoTATTP opposite NaM or G in templates are 0.77 × 10 ^8^ and 2.14 × 10 ^8^ respectively (Figure 2 and Table 1). More interestingly, the dual pairing capacity of isoTAT with NaM and G is exclusive. The % incorporations of disoTATTP opposite A/T/C in templates are all less than 10%, and the Km/Vmax constants of disoTATTP opposite A/T/C in templates are lower than the detectable level. Although nature has such kind of dual/multiple-pairing bases such as hypoxanthine (I) that can acts as a ‘wobble base’ in the third position of codons in tRNA enhancing its reading of anticodon in mRNA, which shows a promising perspective for various applications. There are seldom reports about the design of such kinds of artificial bases that can pair well with a certain expanded genetic letter and natural genetic letters. The finding of isoTAT indicates that the recognitions of UBPs themselves and natural base pairs are not insulated, which can be connected directionally by a proper structure called a bridge base. Moreover, the design of bridge bases can be dependent on the hydrophobic interaction forces, shape complementary, and hydrogen-bond rearrangements that have been shown invaluable tools for current UBP designing. In this regard, the strategy for the combination of parent skeletons and additional hydrogen-bonding acceptor/doner that are used for isoTAT will be also applicable to other unnatural base pairs for different research purposes.

TPT3-NaM is one of the advanced UPBs in the expanded genetic letters (2,8,19,20). There were increasing requirements for incorporations of multiple TPT3-NaM UBPs with or without defaulted loci. More UBPs are used as genetic letters which increases the complexity of DNA, but monitoring the DNA containing multiple UBPs with or without defaulted loci by simple and convenient approaches is challenging by existing methods. We develop a dual location method based on the use of bridge base and the inherent bases’ preference of NaM, and our method can be applied to almost all the sequence contexts, allowing to monitor almost all changes in the sequence. So it is a useful approach for detecting more than one UBP on the unknown or default positions. Besides, the applications of bridge base to analyze the *in vivo* replicated DNA samples containing TPT3-NaM enable researchers to evaluate the fidelity as well as changes of multiple UBPs at the adjacent sites at the same time, which is also hard to be conducted by the biotin-shift assays that are mainly used currently. Considering the unexpected complexity that may be encountered when using TPT3-NaM to recode various proteins and the emerging sequence-dependence properties that may be worse than that of *in vitro* applications (15,27,32), assessments and optimizations of sequence contexts around one and more TPT3-NaM UBPs may be necessary steps before protein expressions. Therefore, our bridge base method offers a simple and convenient determining approach.

5SICS-NaM was used as a marker to identify DNA lesions (22). The single lesion site was identified using Sanger sequencing depending on the terminal signal with no strand location information, and more than one lesion site was identified using α-hemolysin nanopore depending on distinguishing the substituted NaM with post modified reporting group, which possessed a complex process and required expansive apparatus. We provided proof-of-concept and simple biological samples for improving the identification of such multiple lesions marked with UBPs by using our bridge bases. The TPT3 unnatural base can be accurately installed at one or more lesion sites of DNA, and the marked samples can be enriched by TPT3^biotin^. The results indicate that our TPT3^biotin^ can be transferred to C by bridge-base PCR and different DNA lesions can be identified in this model system and biological samples. We also believe that other lesions can also be detected through NaM’ preferences and a bridge base regardless of their pairing abilities. While lesions in biological samples may be in one strand of double-strand DNA with low abundance, the effect of the other strand and normal sequences without lesions should be taken into account. So our enriched step seems to be necessary. It is also noteworthy that the marked samples bearing dTPT3^biotin^ can be directly used as the template for PCR with the bridge base disoTATTP with no requirements for removing the linker and attached streptavidin, simplifying the tedious streptavidin-trapping-then-cleavage process. The method will be more easily operated and generally applied in ordinary biological labs, owing to its total compatibility with the commercialized sequencing technology and no requirements for specialized instruments. In all, our method can provide a simple and convenient dual location of NaM-TPT3 pair for multiple lesions detection. Such a simple dual location platform with the capacity to determine/sequence multiple TPT3-NaM UBPs can therefore be used in various sequence contexts of nucleic acid for different research purposes.

## DATA AVAILABILITY

Raw sequencing data for experiments from this study are available at Bioproject/SRA (BioProject ID PRJNA942583).

## Supplementary Material

gkad218_Supplemental_Files
