# Supplementary material for: Locating, tracing and sequencing multiple expanded genetic letters in complex DNA context via a bridge-base approach
Source: Nucleic Acids Res. 2023 Mar 27;51(9):e52. doi: 10.1093/nar/gkad218 (PMC10201413; doi:10.1093/nar/gkad218)
Supplement: gkad218_Supplemental_Files [file gkad218_supplemental_files.zip › TAT-Supporting information-final(1)(1)(1)-1.pdf]

Supporting Information:

Locating, Tracing, and Sequencing Multiple Expanded Genetic Letters in Complex DNA Context via a Bridge-Base Approach

Honglei Wang,[1,2†] Wuyuan Zhu,[1†] Chao Wang,[1] Xiaohuan Li,[1] Luying Wang,[1] Bianbian Huo,[1,2] Hui Mei,[3] Anlian Zhu,[1] Guisheng Zhang,[1] Lingjun Li [1,2 *]

[1] Henan Key Laboratory of Organic Functional Molecule and Drug Innovation, Collaborative Innovation Center of Henan Province for Green Manufacturing of Fine Chemicals, School of Chemistry and Chemical Engineering, Key Laboratory of Green Chemical Media and Reactions, Ministry of Education, Henan Normal University, Xinxiang, Henan 453007, China. [2] State Key Laboratory of Cell Differentiation Regulation and Target Drug, Henan Normal University, Xinxiang 453007, China. [3] Shenzhen Key Laboratory of Synthetic Genomics, Guangdong Provincial Key Laboratory of Synthetic Genomics, CAS Key Laboratory of Quantitative Engineering Biology, Shenzhen Institute of Synthetic Biology, Shenzhen Institutes of Advanced Technology, Chinese Academy of Sciences, Shenzhen 518055, China.

†Contributed equally to this work

*To whom correspondence should be addressed. Tel: +863733326335; Fax: +863733326335; Email: lingjunlee@htu.edu.cn

Table of Contents

Scheme 1 and detailed synthesis procedure of disoTATTP...2-4
Scheme 2 and detailed synthesis procedure of TPT3^{biotin}...5
NMR Spectra...6-12
MALDI-TOF mass spectral data..13
Sequences used in this study...14-23
Supplementary Figures...23-44
References...44

Detailed Synthesis Procedures for disoTATTP.

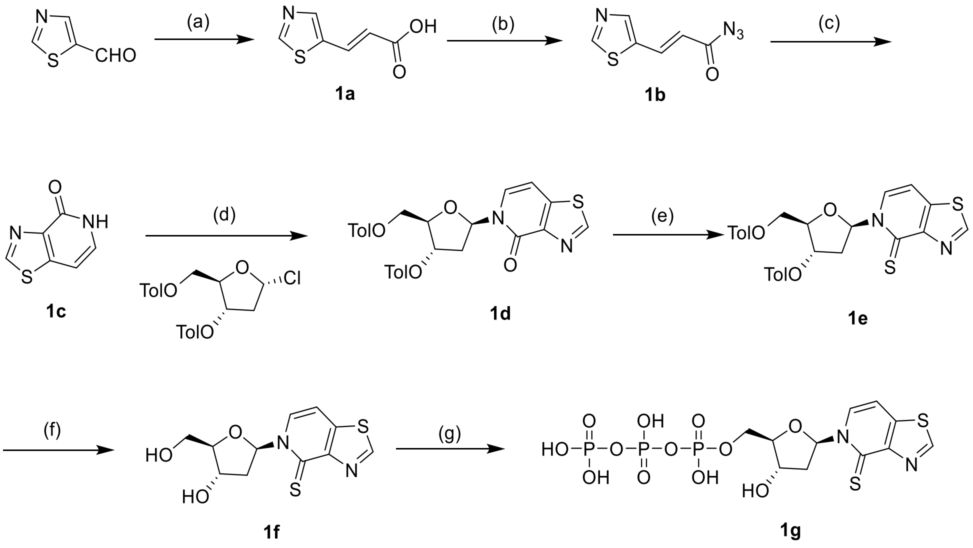

Scheme 1. (a) Pyridine, 100°C, 12 h, 87.5%; (b) THF, Et_3N, DPPA, 0°C-rt, 4 h, 78.4%; (c) diphenyl ether, 250°C, 1 h, 45.2%; (d) N,O-bis(TMS)acetamide, $SnCl_4$, CH_2Cl_2, 3 h, 42.9%; (e) Lawesson's reagent, THF, reflux, 80°C, 12 h, 45.6%; (f) CH_3ONa, CH_3OH, rt, 1 h, 70.7%; (g) Proton sponge, $POCl_3$, $(Bu_4N)_3HP_2O_7$, Bu_3N, $(MeO)_3PO$, DMF, -20°C, 11.2%.

The nucleobase analog **1c** was synthesized as shown in Scheme 1 based on literature(1,2). Briefly, the condensation of thiazole-5-carboxaldehyde (10.0 g, 88.4 mmol) with malonic acid (13.8 g, 132.7 mmol) was in 50 mL pyridine at 100 ℃ for 12 h and was catalyzed by piperidine (1.1 g, 13.3 mmol). Then the product **1a** was precipitated by adding 3 M HCl dropwise. The solution was filtered and the residue was washed with cold water and dried in a vacuum to afford **1a** as a white solid (12 g, 87.5%). Compound **1a** (12 g, 77.3 mmol), 50 mL tetrahydrofuran, and triethylamine (12.9 mL, 92.8 mmol) were mixed by turn, and then diphenyl azide phosphate (18.3 mL, 85.1 mmol) was added dropwise in an ice bath. After reaction at room temperature for 4 h, compound **1b** (10.9 g, 78.4%) was obtained by column chromatography as a white solid. The **1b** (5 g, 27.7 mmol) dissolved in CH_2Cl_2 was added dropwise to 200 mL diphenyl ether at 250 ℃, then proceeded for 1 h. Compound **1c** (1.9 g, 45.2%) was obtained by column chromatography as a

yellowish-brown solid. **1a** ^{1}H NMR (400 MHz, Methanol-*d$_4$*) δ 9.04 (s, 1H), 8.11 (s, 1H), 7.89-7.85 (d, *J* = 16 Hz, 1H), 6.33-6.29 (d, *J* = 16 Hz, 1H). ^{13}C NMR (101 MHz, Methanol-*d$_4$*) δ 168.04, 155.98, 145.47, 135.06, 133.66, 121.07. **1b** ^{1}H NMR (400 MHz, $CDCl_3$) δ 8.82 (s, 1H), 8.03 (s, 1H), 7.88-7.84 (d, *J* = 16 Hz, 1H), 6.21-6.17 (d, *J* = 16 Hz, 1H). ^{13}C NMR (101 MHz, $CDCl_3$) δ 171.14, 155.59, 147.42, 135.35, 134.44, 121.53. **1c** ^{1}H NMR (400 MHz, DMSO-*d$_6$*) δ 11.70 (s, 1H), 9.14 (s, 1H), 7.37-7.35 (d, *J* = 8 Hz, 1H), 6.96-6.95 (d, *J* = 4 Hz, 1H). ^{13}C NMR (101 MHz, DMSO-*d$_6$*) δ 158.00, 153.54, 144.78, 144.51, 131.46, 100.06.

Compound **1d. 1C** (170 mg, 1.12 mmol) dissolved in 5 mL CH_2Cl_2 was mixed with bis(trimethylsilyl)acetamide (250 mg, 1.23 mmol) under nitrogen atmosphere. After stirring for 1 h at room temperature, 3,5-bis(toluoyl)-2-deoxyribosyl chloride (478.4 mg, 1.23 mmol) was added, and then the reaction mixture was cooled to 0 ℃ and $SnCl_4$ (145.4 mg, 0.56 mmol) was added. The reaction proceeded with stirring for 3 h at room temperature, then the reaction mixture was extracted with CH_2Cl_2 and saturated salt water, dried by Na_2SO_4, and separated out solvent by negative pressure. **1d** was afforded from the crude product by column chromatography as white solid (242 mg, 42.9%). ^{1}H NMR (400 MHz, $CDCl_3$) δ 8.83 (s, 1H), 7.98-7.96 (d, *J* = 8 Hz, 2H), 7.89-7.87 (d, *J* = 8 Hz, 2H), 7.70-7.68 (d, *J* = 8 Hz, 1H), 7.29-7.27 (d, *J* = 8 Hz, 2H), 7.21-7.19 (d, *J* = 8 Hz, 2H), 6.85-6.82 (q, *J* = 4 Hz, 1H), 6.65-6.63 (d, *J* = 4 Hz, 1H), 5.66-5.63 (m, 1H), 4.79-4.68 (m, 2H), 4.64-4.62 (q, *J* = 4 Hz, 1H), 3.07-3.02 (m, 1H), 2.43 (s, 3H), 2.39 (s, 3H), 2.36-2.29 (m, 1H). ^{13}C NMR (101 MHz, $CDCl_3$) δ 166.20, 166.16, 157.15, 151.82, 144.51, 144.31, 144.15, 143.09, 129.91, 129.57, 129.33, 129.30, 127.99, 126.64, 126.39, 100.06, 86.08, 83.28, 75.10, 67.79, 64.34, 46.85, 39.43, 33.55, 21.76, 21.71, 20.62. HRMS (ESI$^+$) m/z calcd for $C_{27}H_{25}N_2O_6S$ (M+H$^+$) 505.5645, found 505.1428.

Compound **1e. 1d** (56 mg, 0.11 mmol), Lawesson's reagent (67.4 mg, 0.17 mmol), and 8 mL tetrahydrofuran were mixed by turn under nitrogen atmosphere. The reaction was carried out at 80 ℃ for 12 h, and column chromatography was employed

to afford **1e** as yellowish solid (29 mg, 45.6%). ^{1}H NMR (400 MHz, $CDCl_3$) δ 8.99 (s, 1H), 8.17-8.16 (d, *J* = 4 Hz, 1H), 7.99-7.97 (m, 2H), 7.89-7.87 (m, 2H), 7.45-7.42 (q, *J* = 4 Hz, 1H), 7.29-7.26 (d, *J* = 12 Hz, 2H), 7.22-7.20 (d, *J* = 8 Hz, 2H), 7.04-7.02 (d, *J* = 8 Hz, H), 5.65-5.64 (m, 1H), 4.87-4.74 (m, 2H), 4.71-4.68 (m, 1H), 3.48-3.42 (m, 1H), 2.44 (s, 3H), 2.40 (s, 3H), 2.30-2.22 (m, 1H). ^{13}C NMR (101 MHz, $CDCl_3$) δ 166.16, 158.08, 154.71, 154.44, 154.07, 150.09, 144.58, 144.47, 140.83, 138.36, 131.38, 130.06, 129.93, 129.59, 129.39, 129.33, 127.50, 126.53, 126.31, 109.76, 105.61, 97.58, 91.40, 84.42, 83.81, 74.55, 64.08, 38.79, 29.71, 21.78, 21.72. HRMS (ESI^+) m/z calcd for $C_{27}H_{25}N_2O_2S_2$ ($M+H^+$) 521.6255, found 521.1199.

Compound **1f. 1e** (52 mg, 0.1 mmol) dissolved in 2 mL methanol was mixed with NaOMe (21.6 mg, 0.4 mmol). The reaction was carried out with stirring for 1 h at room temperature, the mixture was subjected to column chromatography to afford compound **1f** as yellowish solid (20 mg, 70.7%). ^{1}H NMR (400 MHz, Methanol-d_4) δ 9.19 (s, 1H), 8.58-8.56 (d, *J* = 8 Hz, 1H), 7.44-7.42 (d, *J* = 8 Hz, 1H), 7.31-7.28 (t, *J* = 4, 1H), 4.45-4.41 (m, 1H), 4.09-4.06 (q, *J* = 4 Hz, 1H), 3.96-3.82 (m, 1H), 2.83-2.77 (m, 1H), 2.17-2.11 (m, 1H). ^{13}C NMR (101 MHz, Methanol-d_4) δ 174.35, 155.92, 153.70, 138.96, 131.42, 105.74, 91.13, 88.25, 69.84, 60.79, 41.13. HRMS (ESI^+) m/z calcd for $C_{11}H_{13}N_2O_3S_2$ ($M+H^+$), 285.3555, found, 285.0362.

Compound **1g.** Under nitrogen atmosphere, **1f** (10 mg, 0.035 mmol) and proton sponge (10.1 mg, 0.046 mmol) were added to 160 μL trimethyl phosphate and cooled to -15 ℃, then added $POCl_3$ (7.1 mg, 0.046 mmol) and kept the reaction system at -15 ℃ for 3 h. Tributylammonium pyrophosphate (162.4 mg, 0.18 mmol) dissolved in 360 μL DMF and tributylamine (38.9 mg, 0.21 mmol) were added to the reaction mixture, and the reaction system was slowly warmed to 0 ℃ with 30 min and quenched subsequently by the addition of 0.5 M TEAB (aqueous $Et_3NH_2CO_3$, pH 7.5). The isolation of the product was by a DEAE Sephadex column (GE Healthcare) and then reverse-phase (C18) HPLC (1.8 mg, 11.2%). ^{31}P NMR (162 MHz, D_2O) δ -9.10, -11.27 (d, *J* = 19.4 Hz, α-P), -22.85 (t, *J* = 21.1 Hz, β-P). MS (MALDI-TOF$^-$, matrix: 2,5-Dihydroxybenzoic acid) (*m/z*): $[M-H]^-$ calcd for $C_{11}H_{14}N_2O_{12}P_3S_2^-$, 523.28, found, 522.90.

Detailed Synthesis Procedures for TPT3^{biotin}.

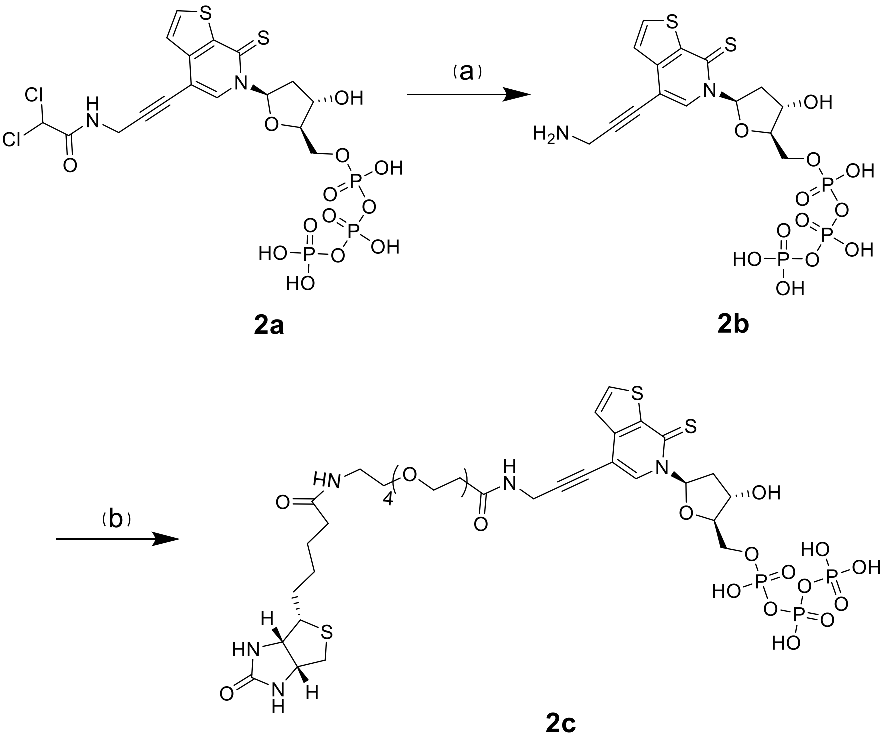

Scheme 2. (a) 30% NH_4OH, rt, 4 h, 100%; (b) NHS-PEG$_4$-biotin, phosphate buffer pH 8, rt, 68.7%.

Compound 2c. Compound **2a** (1.52 mg, 0.0022 mmol) was dissolved in conc. aqueous ammonia (30%). After stirring for 4 h, compound **2b** was afforded by reverse-phase (C18) HPLC (1.26 mg, 100%). Then NHS-PEG$_4$-biotin (2 mg) and **2b** (1.26 mg) were dissolved in phosphate buffer (pH = 8) and kept for 4 h, and compound **2c** was afforded by reverse-phase (C18) HPLC (1.6 mg, 68.7%). MS (MALDI-TOF$^-$, matrix: 2,5-Dihydroxybenzoic acid) (*m/z*): $[M-H]^-$ calcd for $C_{36}H_{54}N_5O_{19}P_3S_3^-$, 1049.95, found, 1049.66.

NMR Spectra

400 MHz ^{1}H NMR spectrum of **Compound 1a** (Methanol-d_4)

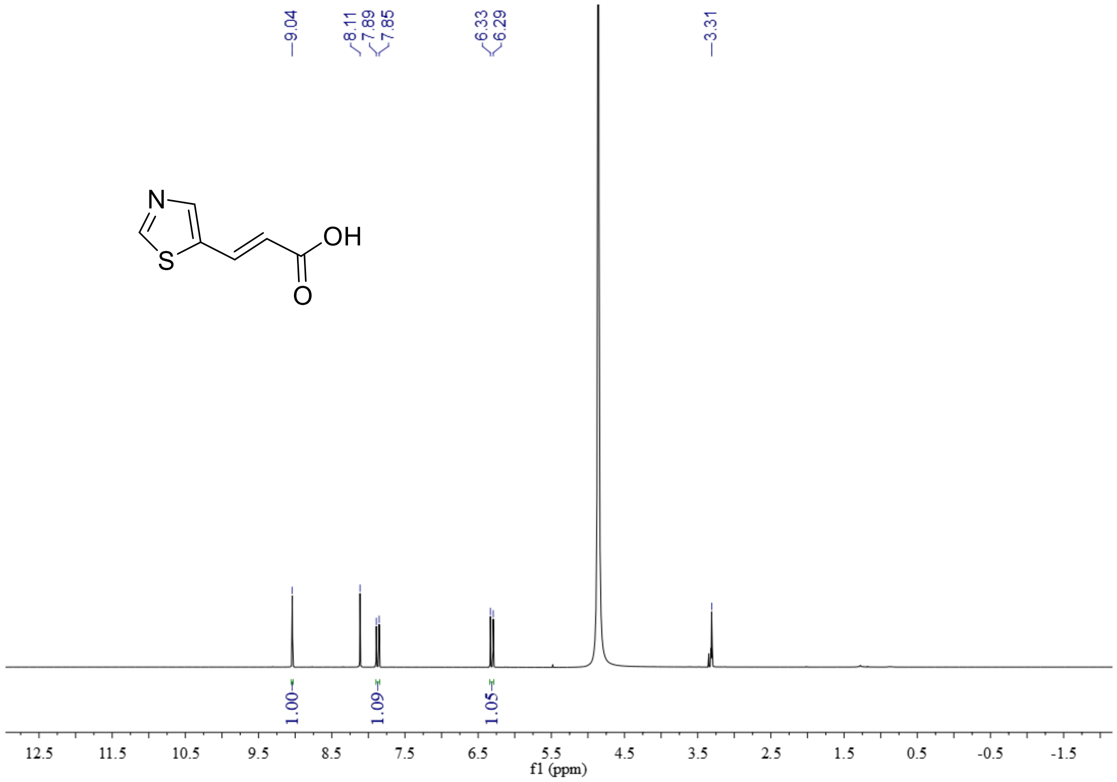

101 MHz ^{13}C NMR spectrum of **Compound 1a** (Methanol-d_4)

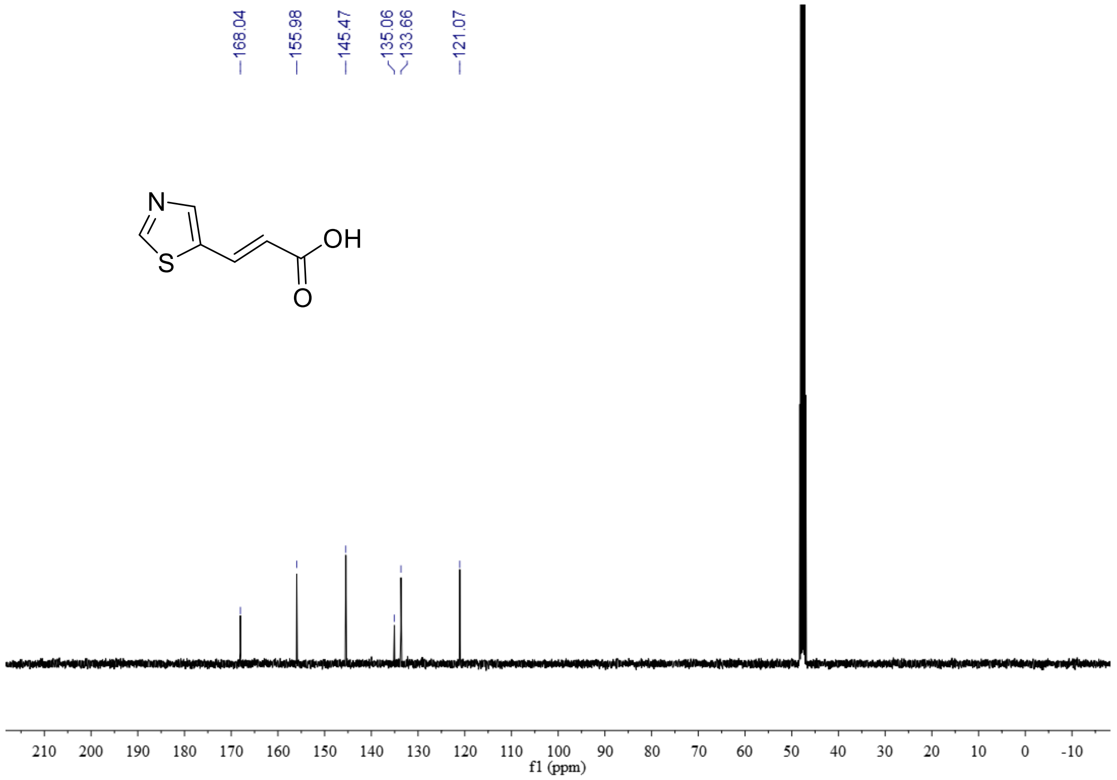

400 MHz ^{1}H NMR spectrum of **Compound 1b** ($CDCl_3$)

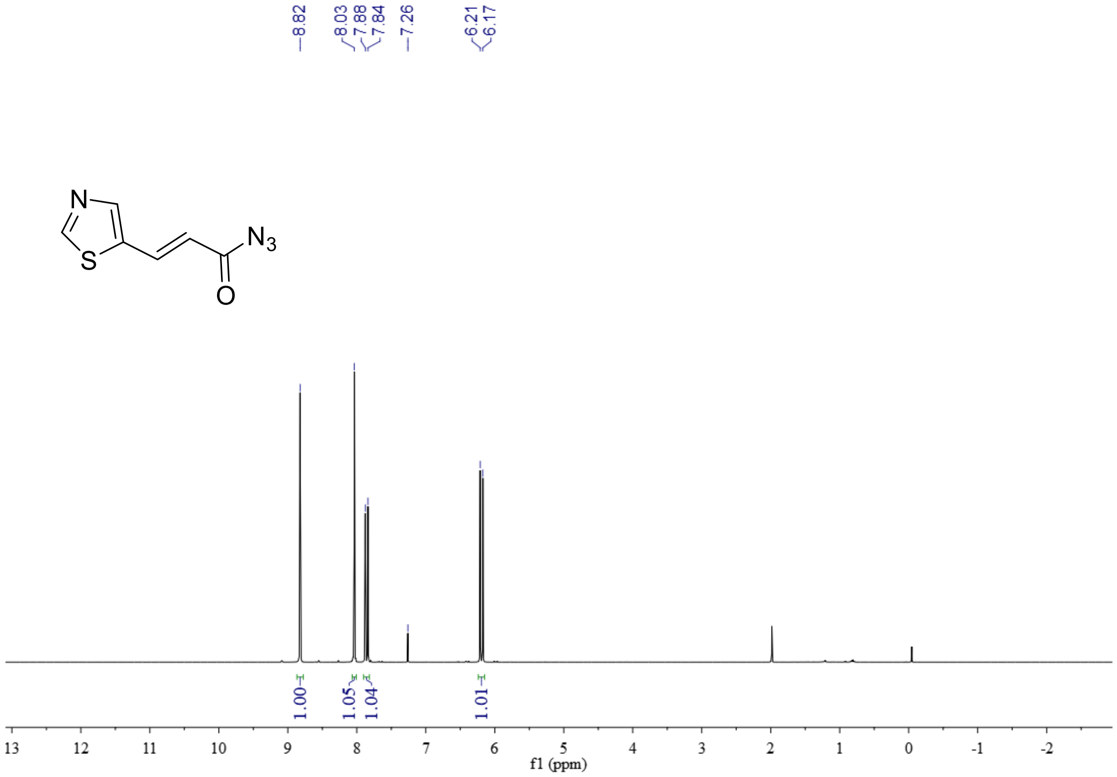

101 MHz ^{13}C NMR spectrum of **Compound 1b** ($CDCl_3$)

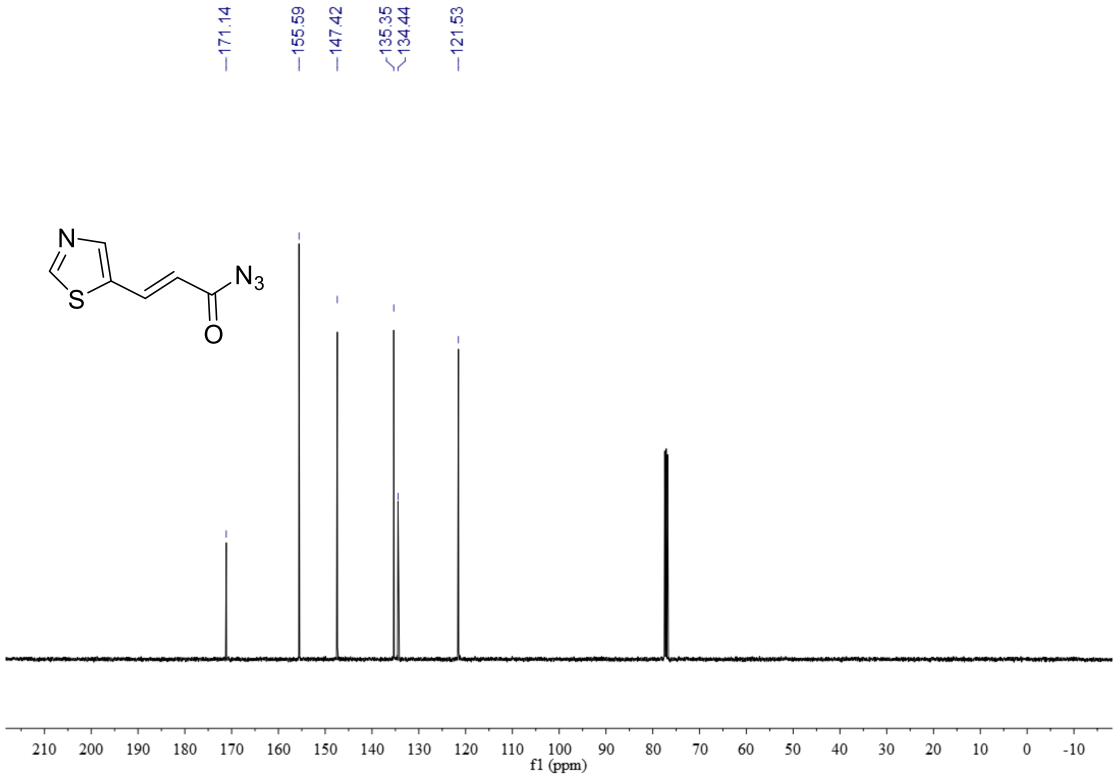

400 MHz ^{1}H NMR spectrum of **Compound 1c** (Methanol-d_4)

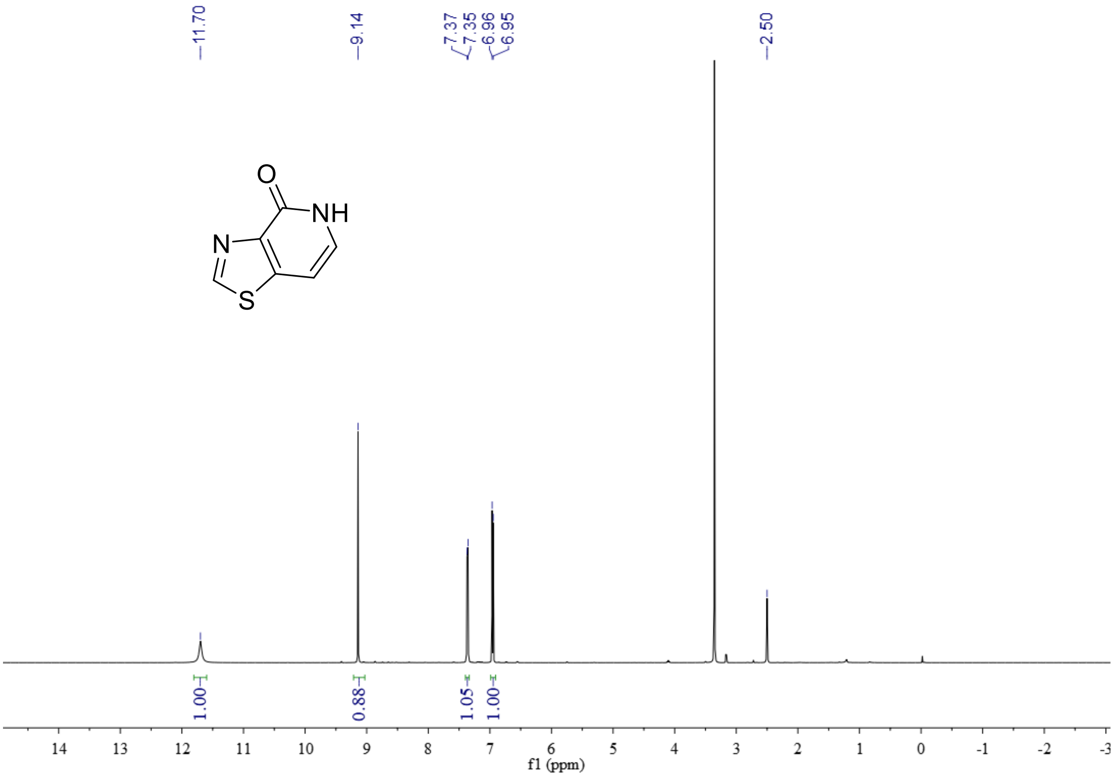

101 MHz ^{13}C NMR spectrum of **Compound 1c** (Methanol-d_4)

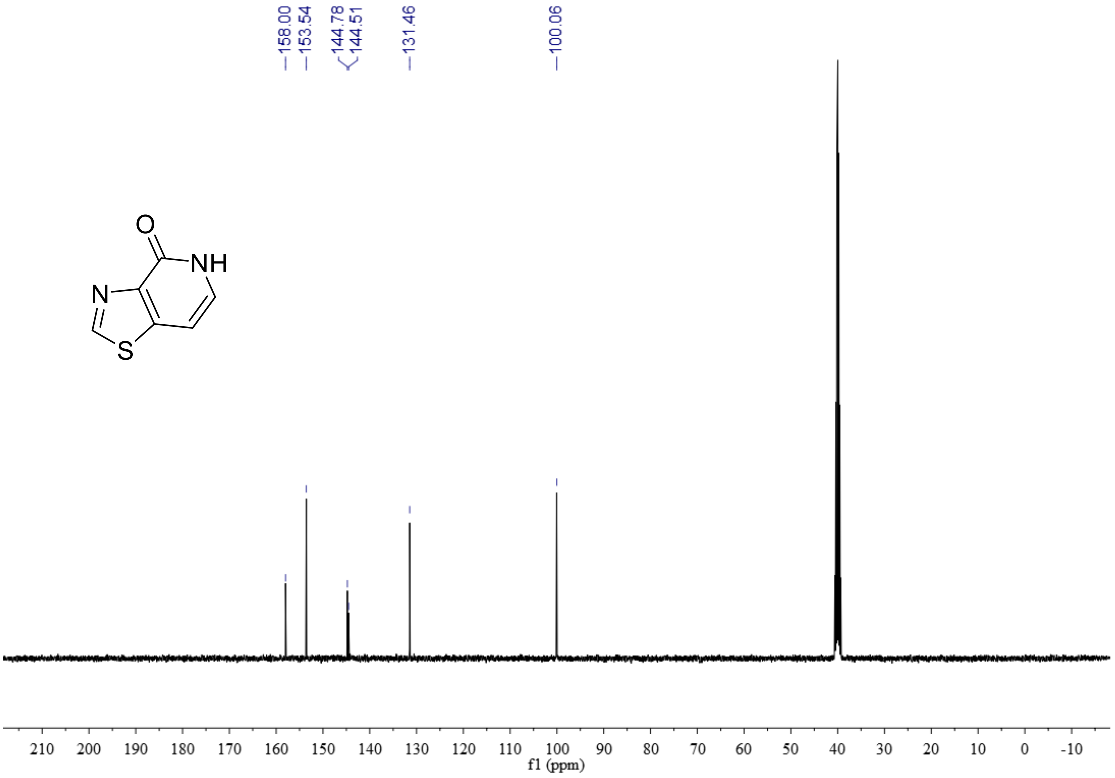

400 MHz ^{1}H NMR spectrum of **Compound 1d** (CDCl$_3$) (β)

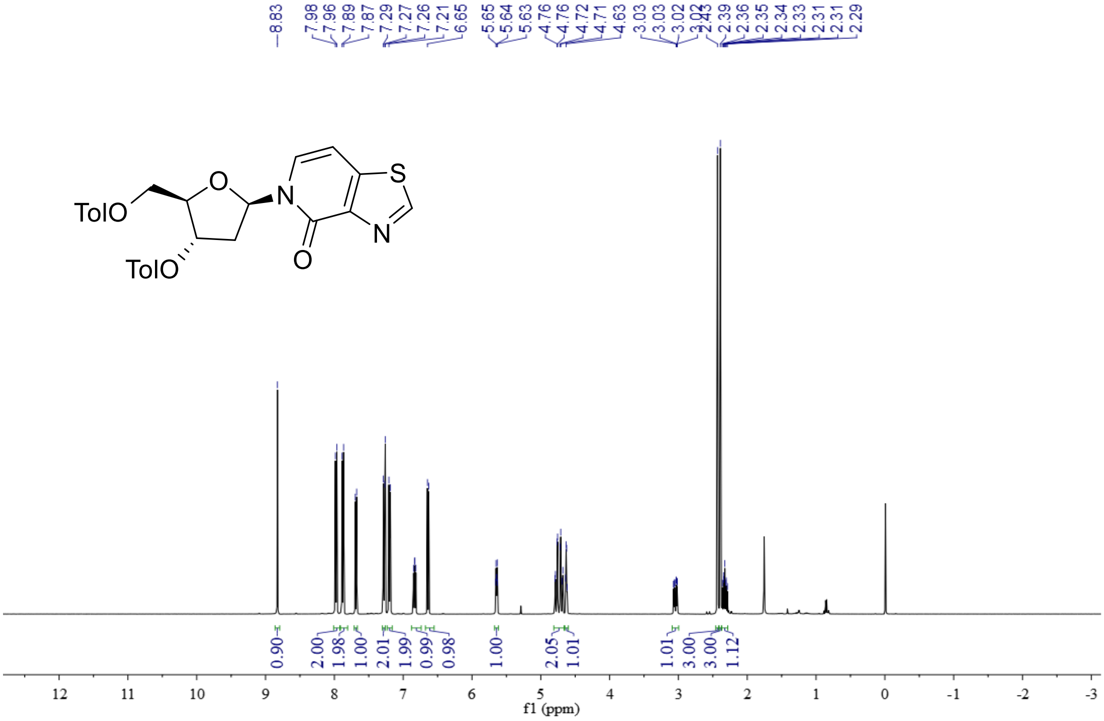

101 MHz ^{13}C NMR spectrum of **Compound 1d** (CDCl$_3$) (β)

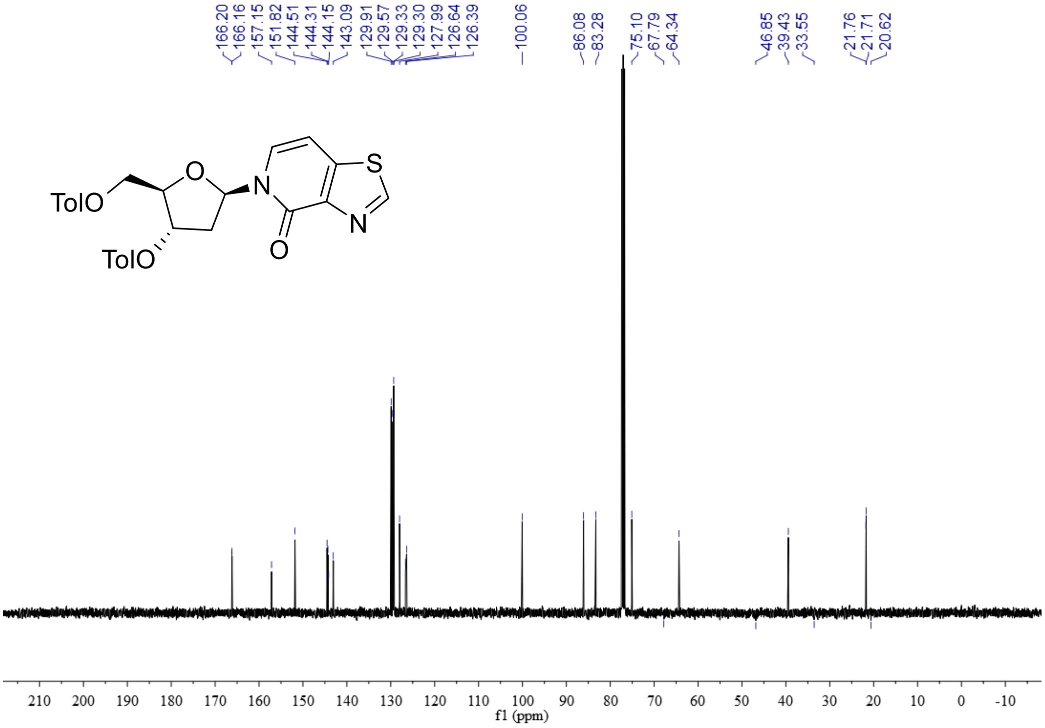

400 MHz ^{1}H NMR spectrum of **Compound 1e** ($CDCl_3$) (β)

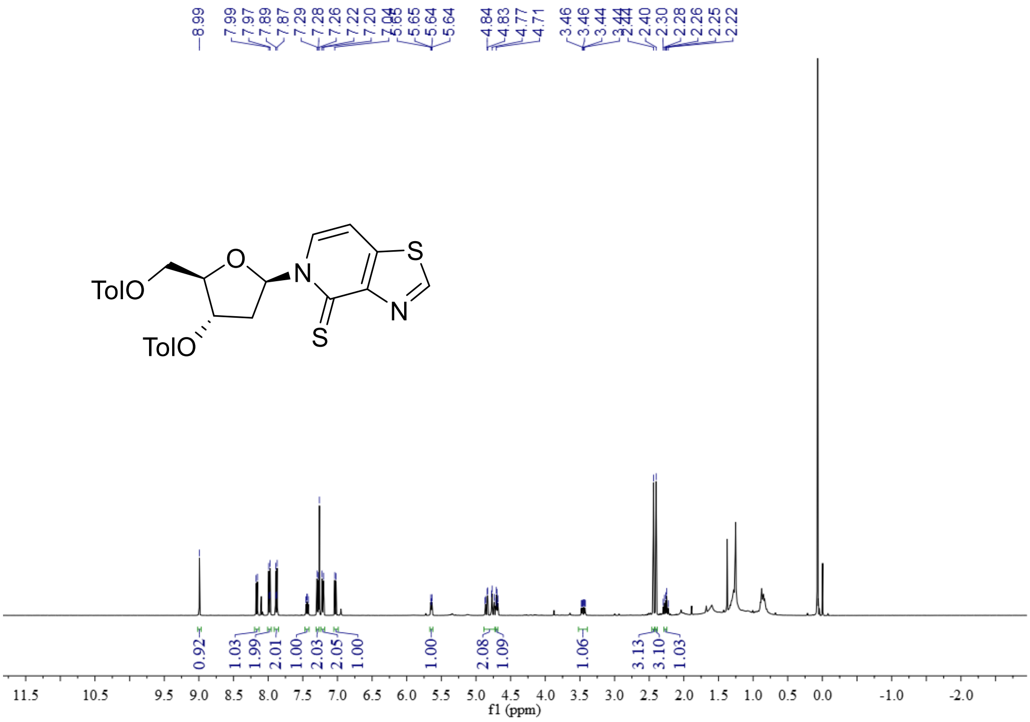

101 MHz ^{13}C NMR spectrum of **Compound 1e** ($CDCl_3$) (β)

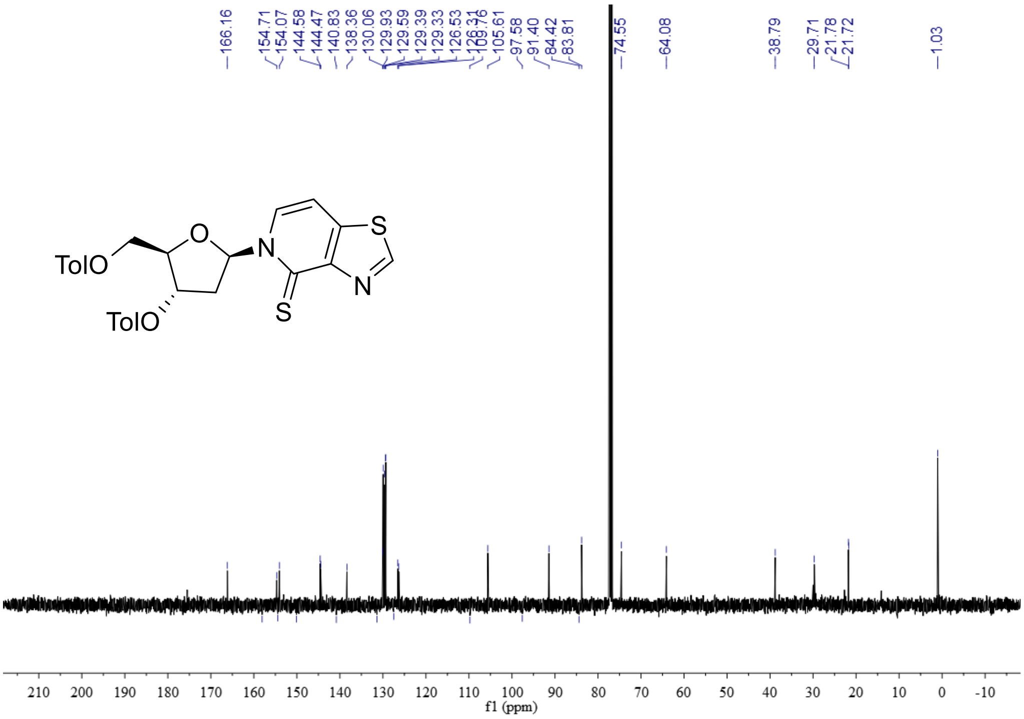

400 MHz ^{1}H NMR spectrum of **Compound 1f** (Methanol-d_4) (β)

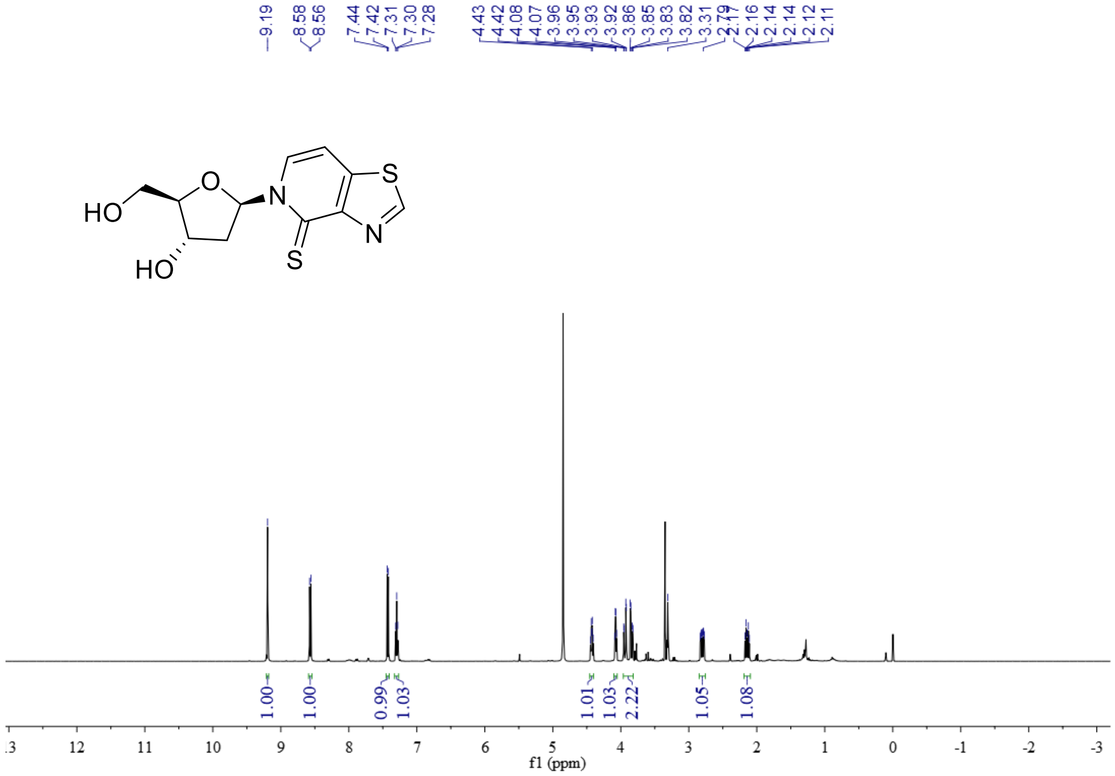

101 MHz ^{13}C NMR spectrum of **Compound 1f** (Methanol-d_4) (β)

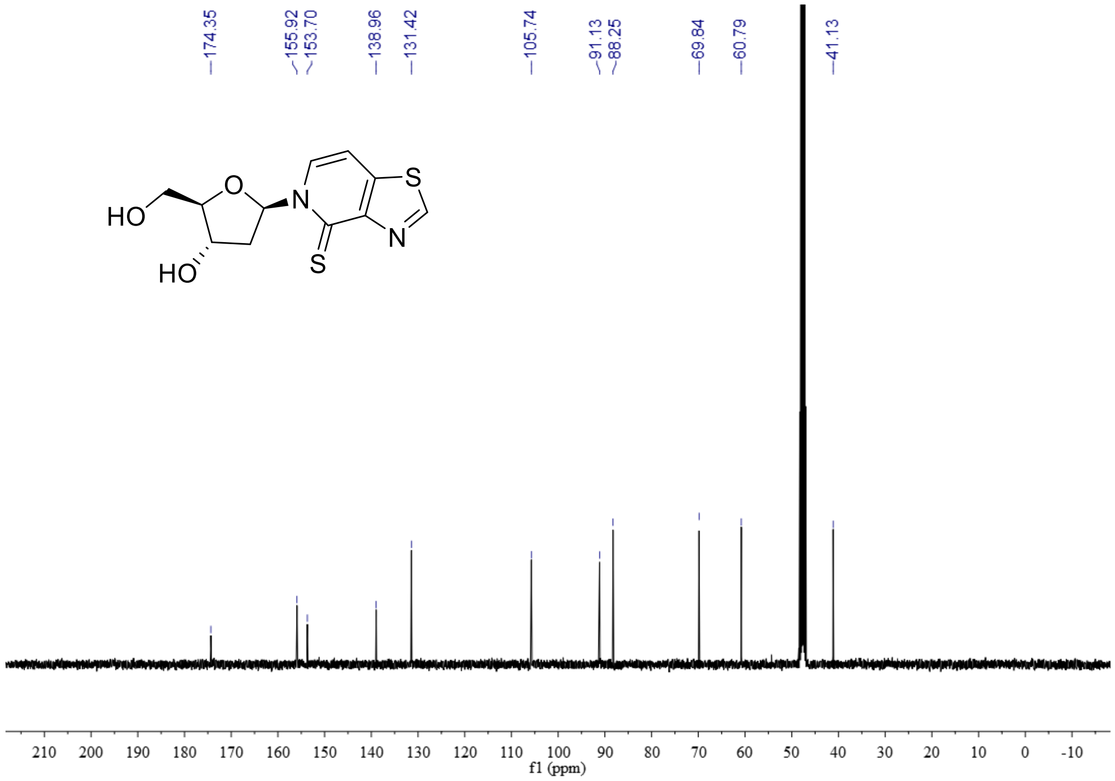

162 MHz ^{31}P NMR spectrum of **Compound 1g** (D_2O) (β)

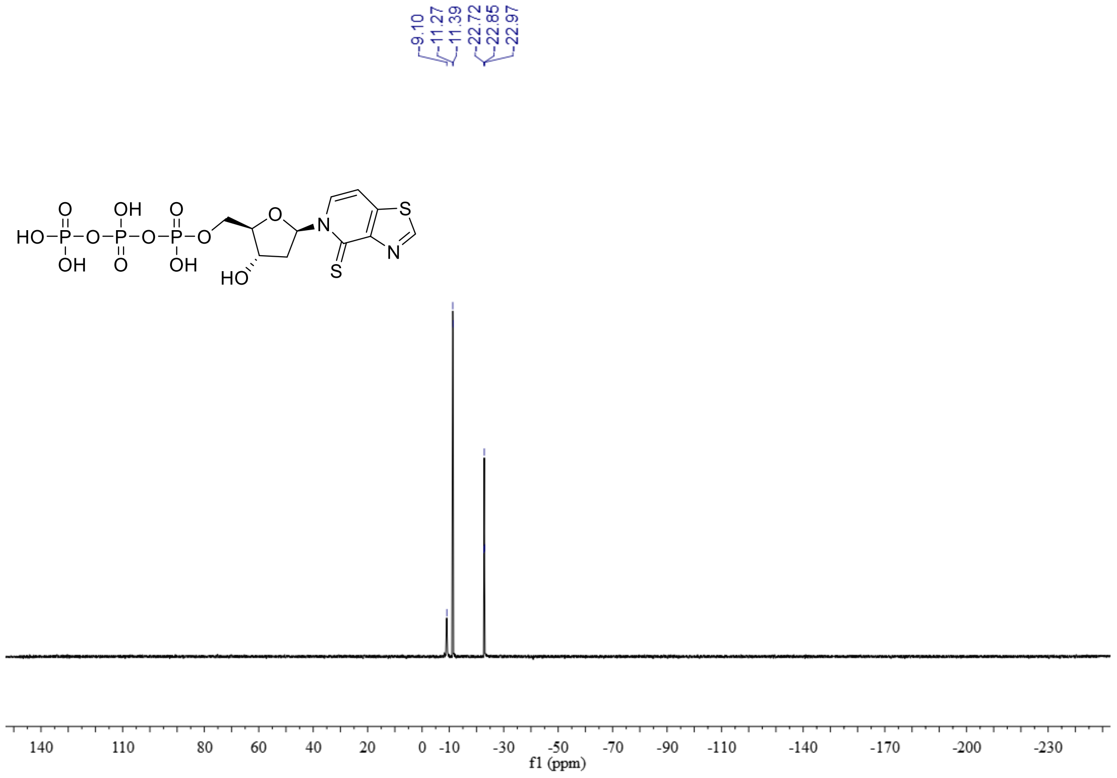

MALDI-TOF mass spectral data

MS of compound **1g**

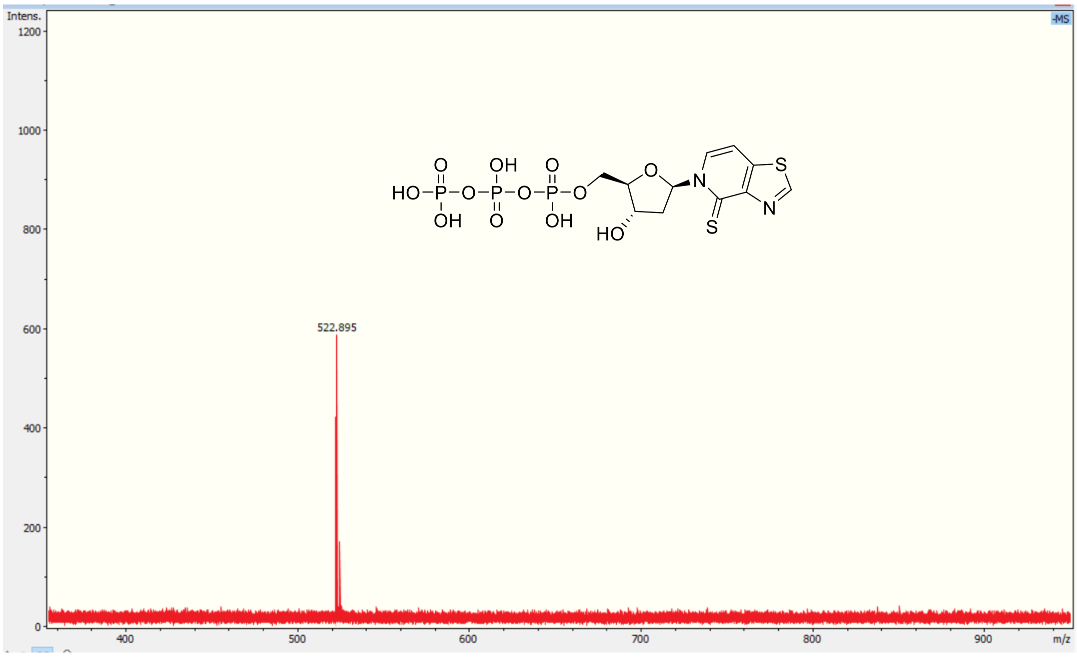

MS of compound **2c**

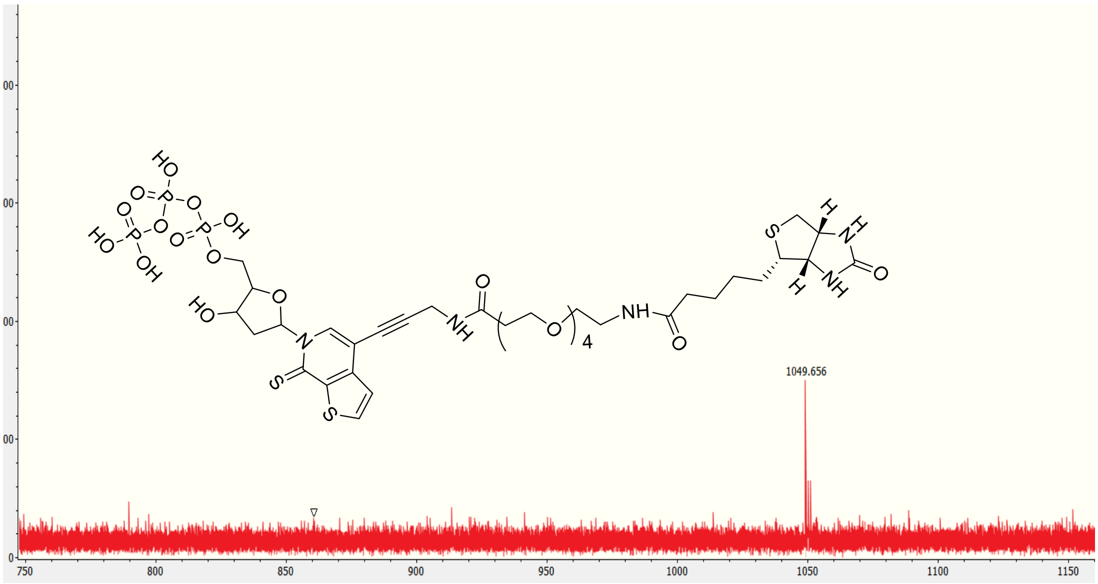

Sequences (5’→3’) used in this study.

Primer and Temples for kinetics

These sequences were according to the literature(3)

Primer: 5`HEX-TAATACGACTCACTATAGGGAGA

Temples

Template-a: CGCTAGGACGGCATTGGATCGNaMTCTCCCTATAGTGAGTCGTATTA

Template-b: CGCTAGGACGGCATTGGATCGATCTCCCTATAGTGAGTCGTATTA

Template-c: CGCTAGGACGGCATTGGATCGCTCTCCCTATAGTGAGTCGTATTA

Template-d: CGCTAGGACGGCATTGGATCGGTCTCCCTATAGTGAGTCGTATTA

Template-e: CGCTAGGACGGCATTGGATCGTTCTCCCTATAGTGAGTCGTATTA

These sequences were newly synthesized

Template-1: ATTGGA***TCG***/NaM/**GGG**C*GGGACCCATAGTAAATCT*

Primer-1: AGATTTACTATGGGTCCCGCCC

Template-2: ATTGGA***ACC***/NaM/***TAG****TAAATCTCCTTCTTAAAGTTA*

Primer-2: TAACTTTAAGAAGGAGATTTACTA

Template-3: ATTGGA***TCG***/NaM/***GGG****CCCTATAGTGAGTCGTATTA*

Primer-3: TAATACGACTCACTATAGGGCCC

Template-4: ATTGGA***GGA***/NaM/***CCA***CCCTATAGTGAGTCGTATTA

Primer-4: TAATACGACTCACTATAGGGTGG

Template-5: ATTGGA***AGA***/NaM/***AAT***CCCTATAGTGAGTCGTATTA

Primer-5: TAATACGACTCACTATAGGGATT

Template-6: ATTGGA***TAA***/NaM/***AGT***CCCTATAGTGAGTCGTATTA

Primer-6: TAATACGACTCACTATAGGGACT

Template-7: ATTGGA***TGG***/NaM/***ATT***CCCTATAGTGAGTCGTATTA

Primer-7: TAATACGACTCACTATAGGGAAT

Template-8: ATTGGA***ACT***/NaM/***TCT***CCCTATAGTGAGTCGTATTA

Primer-8: TAATACGACTCACTATAGGGAGA

Template-9: ATTGGA***ACC***/NaM/***TAG****CCCTATAGTGAGTCGTATTA*

Primer-9: TAATACGACTCACTATAGGGCTA

Template-10: ATTGGA***AAT***/TPT3/***CCA***CCCTATAGTGAGTCGTATTA

Primer-10: TAATACGACTCACTATAGGGTGG

Template-11: ATTGGA***AGA***/TPT3/***AGT***CCCTATAGTGAGTCGTATTA

Primer-11: TAATACGACTCACTATAGGGACT

Template-12: ATTGGA***CTA***/TPT3/***GGT***CCCTATAGTGAGTCGTATTA

Primer-12: TAATACGACTCACTATAGGGACC

These sequences were according to the literature(4)

Primers for PCR of 134 templates

Fend-F: CACACAGGAAACAGCTATGAC

Fend-R: GAAATTAATACGACTCACTATAGG

Primers for Sanger sequencing of 134 templates

FendT-F:

TTTTTTTTTTTTTTTTTTTTTTTTTTTTTTTTTTTTTTTTTTTTTTTTTTTTTTTTTTTCACAC

AGGAAACAGCTATGAC

FendT-R

TTTTTTTTTTTTTTTTTTTTTTTTTTTTTTTTTTTTTTTTTTTTTTTTTTTTTTTTTTTTTGAA

ATTAATACGACTCACTATAGG

134-1N

CACACAGGAAACAGCTATGACCCGGGTTATTACATGCGCTAGCACTTGGAATTCACAA

CCGGNaMATCCCGAGGAAACCATAGTAAATCTCCTTCTTAAAGTTAAGCTTAACCCTAT

AGTGAGTCGTATTAATTTC

134-2N

CACACAGGAAACAGCTATGACCCGGGTTATTACATGCGCTAGCACTTGGNaMATTCAC

AATACTNaMTCTTTAAGGAAACCATAGTAAATCTCCTTCTTAAAGTTAAGCTTAACCCTA

TAGTGAGTCGTATTAATTTC

134-3N

CACACAGGAAACAGCTATGACCCGGGTTATTACATGCGCTAGCACTTGGNaMATTCAC

AATACTNaMTCTTTAAGGAAACCNaMTAGTAAATCTCCTTCTTAAAGTTAAGCTTAACC

CTATAGTGAGTCGTATTAATTTC

134-UN

CACACAGGAAACAGCTATGACCCGGGTTATTACATGCGCTAGCACTTGGAATTCACCA

GACGNNNNaMNNNCGGGACCCATAGTAAATCTCCTTCTTAAAGTTAAGCTTAACCCTAT

AGTGAGTCGTATTAATTTC

134-nature

CACACAGGAAACAGCTATGACCCGGGTTATTACATGCGCTAGCACTTGGAATTCACAAT

ACTTTCTTTAAGGAAACCATAGTAAATCTCCTTCTTAAAGTTAAGCTTAACCCTATAGTG

AGTCGTATTAATTTC

134-2U

CACACAGGAAACAGCTATGACCCGGGTTATTACATGCGCTAGCACTTGGUATTCACAAT

ACTGTCTTTAAGGAAACCUTAGTAAATCTCCTTCTTAAAGTTAAGCTTAACCCTATAGT

GAGTCGTATTAATTTC

Complementary sequence of 134-2U

GAAATTAATACGACTCACTATAGGGTTAAGCTTAACTTTAAGAAGGAGATTTACTAGGG

TTTCCTTAAAGACAGTATTGTGAATGCCAAGTGCTAGCGCATGTAATAACCCGGGTCAT

AGCTGTTTCCTGTGTG

These sequences were according to the literature(5)

Primers for linear pBLUE-T fragment amplification

F: ATCCACTAGTTCTAGAGCGGC

R: AGCTTATCGATACCGTCGACCT

Primers for unnatural fragment cloning

F: AGGTCGACGGTATCGATAAGCTCACACAGGAAACAGCTATGAC

R: GCCGCTCTAGAACTAGTGGATGAAATTAATACGACTCACTATAGG

These sequences were according to the literature(6)

Primers for PCR

KRP-F: CAGGAAACAGCTATGAC

KRP-R: GTAAAACGACGGCCAGT

Primers for sanger sequencing

KRP-dF:

TTTTTTTTTTTTTTTTTTTTTTTTTTTTTTTTTTTTTTTTTTTTTTTTTTTTTTTTTTTTTCAG

GAAACAGCTATGAC

KRP-dR:

TTTTTTTTTTTTTTTTTTTTTTTTTTTTTTTTTTTTTTTTTTTTTTTTTTTTTTTTTTTTTGTA

AAACGACGGCCAGT

KRAS-1U

CAGGAAACAGCTATGACACTCTTGCCTACGCCAUCAGCTCCAACTACACTGGCCGTCG

TTTTAC

KRAS-2U

CAGGAAACAGCTATGACACTCTTUCCTACGCCAUCAGCTCCAACTACACTGGCCGTCG

TTTTAC

KRAS-R: Complementary sequence of KRAS-1U or 2U

TTTTTTTTTTGTAAAACGACGGCCAGTGTAGTTGGAGCTGGTGGCGTAGGCAAGAGTG

TCATAGCTGTTTCCTGTTTTTTTTTT

Sequences of the pBLUE-T plasmid

GTATTGGGAATTCCTGCAGCCCGGGGGATCCACTAGTTCTAGAGCGGCCGCCACCGCG
GTGGAGCTCCAGCTTTTGTTCCCTTTAGTGAGGGTTAATTGCGCGCTTGGCGTAATCAT
GGTCATAGCTGTTTCCTGTGTGAAATTGTTATCCGCTCACAATTCCACACAACATACGA
GCCGGAAGCATAAAGTGTAAAGCCTGGGGTGCCTAATGAGTGAGCTAACTCACATTAA
TTGCGTTGCGCTCACTGCCCGCTTTCCAGTCGGGAAACCTGTCGTGCCAGCTGCATTAA
TGAATCGGCCAACGCGCGGGGAGAGGCGGTTTGCGTATTGGGCGCTCTTCCGCTTCCT
CGCTCACTGACTCGCTGCGCTCGGTCGTTCGGCTGCGGCGAGCGGTATCAGCTCACTC
AAAGGCGGTAATACGGTTATCCACAGAATCAGGGGATAACGCAGGAAAGAACATGTGA

GCAAAAGGCCAGCAAAAGGCCAGGAACCGTAAAAAGGCCGCGTTGCTGGCGTTTTTC
CATAGGCTCCGCCCCCCTGACGAGCATCACAAAAATCGACGCTCAAGTCAGAGGTGGC
GAAACCCGACAGGACTATAAAGATACCAGGCGTTTCCCCCTGGAAGCTCCCTCGTGCG
CTCTCCTGTTCCGACCCTGCCGCTTACCGGATACCTGTCCGCCTTTCTCCCTTCGGGAA
GCGTGGCGCTTTCTCATAGCTCACGCTGTAGGTATCTCAGTTCGGTGTAGGTCGTTCGC
TCCAAGCTGGGCTGTGTGCACGAACCCCCCGTTCAGCCCGACCGCTGCGCCTTATCCG
GTAACTATCGTCTTGAGTCCAACCCGGTAAGACACGACTTATCGCCACTGGCAGCAGC
CACTGGTAACAGGATTAGCAGAGCGAGGTATGTAGGCGGTGCTACAGAGTTCTTGAAG
TGGTGGCCTAACTACGGCTACACTAGAAGGACAGTATTTGGTATCTGCGCTCTGCTGAA
GCCAGTTACCTTCGGAAAAAGAGTTGGTAGCTCTTGATCCGGCAAACAAACCACCGCT
GGTAGCGGTGGTTTTTTTGTTTGCAAGCAGCAGATTACGCGCAGAAAAAAAGGATCTC
AAGAAGATCCTTTGATCTTTTCTACGGGGTCTGACGCTCAGTGGAACGAAAACTCACG
TTAAGGGATTTTGGTCATGAGATTATCAAAAAGGATCTTCACCTAGATCCTTTTAAATTA
AAAATGAAGTTTTAAATCAATCTAAAGTATATATGAGTAAACTTGGTCTGACAGTTACC
AATGCTTAATCAGTGAGGCACCTATCTCAGCGATCTGTCTATTTCGTTCATCCATAGTTG
CCTGACTCCCCGTCGTGTAGATAACTACGATACGGGAGGGCTTACCATCTGGCCCCAGT
GCTGCAATGATACCGCGAGACCCACGCTCACCGGCTCCAGATTTATCAGCAATAAACC
AGCCAGCCGGAAGGGCCGAGCGCAGAAGTGGTCCTGCAACTTTATCCGCCTCCATCCA
GTCTATTAATTGTTGCCGGGAAGCTAGAGTAAGTAGTTCGCCAGTTAATAGTTTGCGCA
ACGTTGTTGCCATTGCTACAGGCATCGTGGTGTCACGCTCGTCGTTTGGTATGGCTTCA
TTCAGCTCCGGTTCCCAACGATCAAGGCGAGTTACATGATCCCCCATGTTGTGCAAAA
AAGCGGTTAGCTCCTTCGGTCCTCCGATCGTTGTCAGAAGTAAGTTGGCCGCAGTGTTA
TCACTCATGGTTATGGCAGCACTGCATAATTCTCTTACTGTCATGCCATCCGTAAGATGC
TTTTCTGTGACTGGTGAGTACTCAACCAAGTCATTCTGAGAATAGTGTATGCGGCGACC
GAGTTGCTCTTGCCCGGCGTCAATACGGGATAATACCGCGCCACATAGCAGAACTTTAA
AAGTGCTCATCATTGGAAAACGTTCTTCGGGGCGAAAACTCTCAAGGATCTTACCGCT
GTTGAGATCCAGTTCGATGTAACCCACTCGTGCACCCAACTGATCTTCAGCATCTTTTA
CTTTCACCAGCGTTTCTGGGTGAGCAAAAACAGGAAGGCAAAATGCCGCAAAAAAGG
GAATAAGGGCGACACGGAAATGTTGAATACTCATACTCTTCCTTTTTCAATATTATTGAA
GCATTTATCAGGGTTATTGTCTCATGAGCGGATACATATTTGAATGTATTTAGAAAAATA
AACAAATAGGGGTTCCGCGCACATTTCCCCGAAAAGTGCCACCTGACGCGCCCTGTAG
CGGCGCATTAAGCGCGGCGGGTGTGGTGGTTACGCGCAGCGTGACCGCTACACTTGCC
AGCGCCCTAGCGCCCGCTCCTTTCGCTTTCTTCCCTTCCTTTCTCGCCACGTTCGCCGG
CTTTCCCCGTCAAGCTCTAAATCGGGGGCTCCCTTTAGGGTTCCGATTTAGTGCTTTAC
GGCACCTCGACCCCAAAAAACTTGATTAGGGTGATGGTTCACGTAGTGGGCCATCGCC
CTGATAGACGGTTTTTCGCCCTTTGACGTTGGAGTCCACGTTCTTTAATAGTGGACTCT
TGTTCCAAACTGGAACAACACTCAACCCTATCTCGGTCTATTCTTTTGATTTATAAGGGA
TTTTGCCGATTTCGGCCTATTGGTTAAAAAATGAGCTGATTTAACAAAAATTTAACGCG
AATTTTAACAAAATATTAACGCTTACAATTTCCATTCGCCATTCAGGCTGCGCAACTGTT
GGGAAGGGCGATCGGTGCGGGCCTCTTCGCTATTACGCCAGCTGGCGAAAGGGGGAT
GTGCTGCAAGGCGATTAAGTTGGGTAACGCCAGGGTTTTCCCAGTCACGACGTTGTAA
AACGACGGCCAGTGAGCGCGCGTAATACGACTCACTATAGGGCGAATTGGGTACCGGG
CCCCCCCTCGAGGTCGACGGTATCGATAAGCTTGATATCGAATTCCCAATAC

The sequence of the *Pt*NTT2 plasmid according to the previous report(7)

ATCTCAATTGGTATAGTGATTAAAATCACCTAGACCAATTGAGATGTATGTCTGAATTAG
TTGTTTTCAAAGCAAATGAACTAGCGATTAGTCGCTATGACTTAACGGAGCATGAAACC
AAGCTAATTTTATGCTGTGTGGCACTACTCAACCCCACGATTGAAAACCCTACAAGGA
AAGAACGGACGGTATCGTTCACTTATAACCAATACGCTCAGATGATGAACATCAGTAGG
GAAAATGCTTATGGTGTATTAGCTAAAGCAACCAGAGAGCTGATGACGAGAACTGTGG
AAATCAGGAATCCTTTGGTTAAAGGCTTTGAGATTTTCCAGTGGACAAACTATGCCAA
GTTCTCAAGCGAAAAATTAGAATTAGTTTTTAGTGAAGAGATATTGCCTTATCTTTTCCA
GTTAAAAAAATTCATAAAATATAATCTGGAACATGTTAAGTCTTTTGAAAACAAATACT
CTATGAGGATTTATGAGTGGTTATTAAAAGAACTAACACAAAAGAAAACTCACAAGGC
AAATATAGAGATTAGCCTTGATGAATTTAAGTTCATGTTAATGCTTGAAAATAACTACCA
TGAGTTTAAAAGGCTTAACCAATGGGTTTTGAAACCAATAAGTAAAGATTTAAACACTT
ACAGCAATATGAAATTGGTGGTTGATAAGCGAGGCCGCCCGACTGATACGTTGATTTTC
CAAGTTGAACTAGATAGACAAATGGATCTCGTAACCGAACTTGAGAACAACCAGATAA
AAATGAATGGTGACAAAATACCAACAACCATTACATCAGATTCCTACCTACATAACGGA
CTAAGAAAAACACTACACGATGCTTTAACTGCAAAAATTCAGCTCACCAGTTTTGAGG
CAAAATTTTTGAGTGACATGCAAAGTAAGTATGATCTCAATGGTTCGTTCTCATGGCTC
ACGCAAAAACAACGAACCACACTAGAGAACATACTGGCTAAATACGGAAGGATCTGA
GGTTCTTATGGCTCTTGTATCTATCAGTGAAGCATCAAGACTAACAAACAAAAGTAGAA
CAACTGTTCACCGTTACATATCAAAGGGAAAACTGTCCATATGCACAGATGAAAACGG
TGTAAAAAAGATAGATACATCAGAGCTTTTACGAGTTTTTGGTGCATTCAAAGCTGTTC
ACCATGAACAGATCGACAATGTAACAGATGAACAGCATGTAACACCTAATAGAACAGG
TGAAACCAGTAAAACAAAGCAACTAGAACATGAAATTGAACACCTGAGACAACTTGT
TACAGCTCAACAGTCACACATAGACAGCCTGAAACAGGCGATGCTGCTTATCGAATCA
AAGCTGCCGACAACACGGGAGCCAGTGACGCCTCCCGTGGGGAAAAAATCATGGCAA
TTCTGGAAGAAATAGCGCCCAATACGCAAACCGCCTCTCCCCGCGCGTTGGCCGATTC
ATTAATGCAGCTGGCACGACAGGTTTCCCGACTGGAAAGCGGGCAGTGAGCGCAACG
CAATTAATGTAAATTTTTCTAAATACATTCAAATATGTATCCGCTCATGAGACAATAACCC
TGCCTGTAGAAATAATTTTGTTTAACTTTAATAAGGAGATATACCATGGGTGGTAGCACC
GTTGCACCGACCACACCGCTGGCAACCGGTGGTGCACTGCGTAAAGTTCGTCAGGCA
GTTTTTCCGATTTATGGCAATCAAGAAGTGACCAAATTTCTGCTGATTGGCAGCATCAA
ATTCTTTATTATCCTGGCACTGACCCTGACCCGTGATACCAAAGATACCCTGATTGTTAC
CCAGTGTGGTGCAGAAGCAATTGCATTTCTGAAAATCTATGGTGTTCTGCCTGCAGCAA
CCGCATTTATTGCACTGTATAGCAAAATGAGCAACGCAATGGGCAAAAAAATGCTGTTT
TATAGCACCTGTATCCCGTTCTTTACCTTTTTTGGTCTGTTCGATGTGTTCATTTATCCGA
ATGCCGAACGTCTGCATCCGAGCCTGGAAGCAGTTCAGGCAATTCTGCCTGGTGGTGC
CGCAAGCGGTGGTATGGCAGTTCTGGCAAAAATTGCAACCCATTGGACCAGCGCACTG
TTTTATGTTATGGCAGAAATCTATAGCAGCGTTAGCGTTGGTCTGCTGTTTTGGCAGTTT
GCAAATGATGTTGTTAATGTGGATCAGGCCAAACGTTTTTATCCGCTGTTTGCACAGAT
GAGCGGTCTGGCACCGGTTCTGGCAGGTCAGTATGTTGTTCGTTTTGCAAGCAAAGCC
GTTAATTTTGAAGCAAGCATGCATCGTCTGACCGCAGCAGTTACCTTTGCAGGTATTAT
GATCTGCATCTTTTATCAGCTGAGCAGCTCATATGTTGAACGTACCGAAAGCGCAAAAC
CGGCAGCAGATAATGAACAGAGCATTAAACCGAAGAAAAAAAAACCGAAAATGTCGA
TGGTGGAAAGCGGTAAATTTCTGGCAAGCAGCCAGTATCTGCGTCTGATTGCAATGCT
GGTTCTGGGTTATGGTCTGAGCATTAACTTTACCGAAATCATGTGGAAAAGCCTGGTGA

AAAAACAGTATCCGGATCCGCTGGATTATCAGCGTTTTATGGGTAATTTTAGCAGCGCA
GTTGGTCTGAGTACCTGCATTGTTATCTTTTTTGGCGTGCATGTTATTCGTCTGCTGGGT
TGGAAAGTTGGTGCCCTGGCAACACCGGGTATTATGGCCATTCTGGCACTGCCGTTTTT
TGCATGTATTCTGCTGGGCCTGGATAGTCCGGCACGTCTGGAAATTGCAGTTATTTTTG
GCACCATTCAGAGCCTGCTGAGCAAAACCAGCAAATATGCACTGTTTGATCCGACCAC
CCAGATGGCATATATCCCGCTGGATGATGAAAGCAAAGTTAAAGGCAAAGCAGCCATT
GATGTTCTGGGTAGCCGTATTGGTAAATCAGGTGGTAGCCTGATTCAGCAGGGTCTGGT
TTTTGTTTTTGGCAATATTATCAATGCCGCACCGGTTGTTGGTGTTGTGTATTATAGCGTT
CTGGTTGCATGGATGAGTGCAGCAGGTCGTCTGAGTGGTCTGTTTCAGGCACAGACCG
AAATGGATAAAGCAGATAAAATGGAAGCCAAAACCAACAAAGAAAAATGATTAACCT
AGGCTGCTGCCACCGCTGAGCAATAAGACTCCTGTTGATAGATCCAGTAATGACCTCA
GAACTCCATCTGGATTTGTTCAGAACGCTCGGTTGCCGCCGGGCGTTTTTTATTGGTGA
GAATTTTTGGCGGATGGCATTTGAGAAGCACACGGTCACACTGCTTCCGGTAGTCAAT
AAACCGGTAAACCAGCAATAGACATAAGCGGCTATTTAACGACCCTGCCCTGAACCGA
CGACCGGGTCGAATTTGCTTTCGAATTTCTGCCATTCATCCGCTTATTATCACTTATTCA
GGCGTAGCACCAGGCGTTTAAGGGCACCAATAACTGCCTTAAAAAAATTACGCCCCGC
CCTGCCACTCATCGCAGTACTGTTGTAATTCATTAAGCATTCTGCCGACATGGAAGCCA
TCACAGACGGCATGATGAACCTGAATCGCCAGCGGCATCAGCACCTTGTCGCCTTGCG
TATAATATTTGCCCATGGTGAAAACGGGGGCGAAGAAGTTGTCCATATTGGCCACGTTT
AAATCAAAACTGGTGAAACTCACCCAGGGATTGGCTGAGACGAAAAACATATTCTCAA
TAAACCCTTTAGGGAAATAGGCCAGGTTTTCACCGTAACACGCCACATCTTGCGAATAT
ATGTGTAGAAACTGCCGGAAATCGTCGTGGTATTCACTCCAGAGCGATGAAAACGTTT
CAGTTTGCTCATGGAAAACGGTGTAACAAGGGTGAACACTATCCCATATCACCAGCTC
ACCGTCTTTCATTGCCATACGAAATTCCGGATGAGCATTCATCAGGCGGGCAAGAATGT
GAATAAAGGCCGGATAAAACTTGTGCTTATTTTTCTTTACGGTCTTTAAAAAGGCCGTA
ATATCCAGCTGAACGGTCTGGTTATAGGTACATTGAGCAACTGACTGAAATGCCTCAAA
ATGTTCTTTACGATGCCATTGGGATATATCAACGGTGGTATATCCAGTGATTTTTTTCTCC
ATTTTAGCTTCCTTAGCTCCTGAAAATCTCGATAACTCAAAAAATACGCCCGGTAGTGA
TCTTATTTCATTATGGTGAAAGTTGGAACCTCTTACGTGCCGATCAACGTCTCATTTTCG
CCAAAAGTTGGCCCAGGGCTTCCCGGTATCAACAGGGACACCAGGATTTATTTATTCTG
CGAAGTGATCTTCCGTCACAGGTATTTATTCGGCGCCTGTAGTGCCATTTACCCCCATTC
ACTGCCAGAGCCGTGAGCGCAGCGAACTGAATGTCACGAAAAAGACAGCGACTCAG
GTGCCTGATGGTCGGAGACAAAAGGAATATTCAGCGATTTGCCCGAGCTTGCGAGGGT
GCTACTTAAGCCTTTAGGGTTTTAAGGTCTGTTTTGTAGAGGAGCAAACAGCGTTTGCG
ACATCCTTTTGTAATACTGCGGAACTGACTAAAGTAGTGAGTTATACACAGGGCTGGGA
TCTATTCTTTTTATCTTTTTTTATTCTTTCTTTATTCTATAAATTATAACCACTTGAATATAA
ACAAAAAAAACACACAAAGGTCTAGCGGAATTTACAGAGGGTCTAGCAGAATTTACA
AGTTTTCCAGCAAAGGTCTAGCAGAATTTACAGATACCCACAACTCAAAGGAAAAGGA
CTAGTAATTATCATTGACTAGCCC

Sequences used for the detection of Apurinic and apyrimidinic (AP) sites in pUC-19 plasmid

Primers used for PCR amplification

Primer F: GCCGGATCAAGAGCTACCAA

Primer R: GCGAAACCCGACAGGACTAT

The sequences of pUC-19 plasmid:

GCGCCCAATACGCAAACCGCCTCTCCCCGCGCGTTGGCCGATTCATTAATGCAGCTGGC
ACGACAGGTTTCCCGACTGGAAAGCGGGCAGTGAGCGCAACGCAATTAATGTGAGTT
AGCTCACTCATTAGGCACCCCAGGCTTTACACTTTATGCTTCCGGCTCGTATGTTGTGTG
GAATTGTGAGCGGATAACAATTTCACACAGGAAACAGCTATGACCATGATTACGCCAA
GCTTGCATGCCTGCAGGTCGACTCTAGAGGATCCCCGGGTACCGAGCTCGAATTCACT
GGCCGTCGTTTTACAACGTCGTGACTGGGAAAACCCTGGCGTTACCCAACTTAATCGC
CTTGCAGCACATCCCCCTTTCGCCAGCTGGCGTAATAGCGAAGAGGCCCGCACCGATC
GCCCTTCCCAACAGTTGCGCAGCCTGAATGGCGAATGGCGCCTGATGCGGTATTTTCTC
CTTACGCATCTGTGCGGTATTTCACACCGCATATGGTGCACTCTCAGTACAATCTGCTCT
GATGCCGCATAGTTAAGCCAGCCCCGACACCCGCCAACACCCGCTGACGCGCCCTGAC
GGGCTTGTCTGCTCCCGGCATCCGCTTACAGACAAGCTGTGACCGTCTCCGGGAGCTG
CATGTGTCAGAGGTTTTCACCGTCATCACCGAAACGCGCGAGACGAAAGGGCCTCGT
GATACGCCTATTTTTATAGGTTAATGTCATGATAATAATGGTTTCTTAGACGTCAGGTGGC
ACTTTTCGGGGAAATGTGCGCGGAACCCCTATTTGTTTATTTTTCTAAATACATTCAAAT
ATGTATCCGCTCATGAGACAATAACCCTGATAAATGCTTCAATAATATTGAAAAAGGAA
GAGTATGAGTATTCAACATTTCCGTGTCGCCCTTATTCCCTTTTTTGCGGCATTTTGCCTT
CCTGTTTTTGCTCACCCAGAAACGCTGGTGAAAGTAAAAGATGCTGAAGATCAGTTGG
GTGCACGAGTGGGTTACATCGAACTGGATCTCAACAGCGGTAAGATCCTTGAGAGTTT
TCGCCCCGAAGAACGTTTTCCAATGATGAGCACTTTTAAAGTTCTGCTATGTGGCGCGG
TATTATCCCGTATTGACGCCGGGCAAGAGCAACTCGGTCGCCGCATACACTATTCTCAG
AATGACTTGGTTGAGTACTCACCAGTCACAGAAAAGCATCTTACGGATGGCATGACAG
TAAGAGAATTATGCAGTGCTGCCATAACCATGAGTGATAACACTGCGGCCAACTTACTT
CTGACAACGATCGGAGGACCGAAGGAGCTAACCGCTTTTTTGCACAACATGGGGGATC
ATGTAACTCGCCTTGATCGTTGGGAACCGGAGCTGAATGAAGCCATACCAAACGACGA
GCGTGACACCACGATGCCTGTAGCAATGGCAACAACGTTGCGCAAACTATTAACTGGC
GAACTACTTACTCTAGCTTCCCGGCAACAATTAATAGACTGGATGGAGGCGGATAAAGT
TGCAGGACCACTTCTGCGCTCGGCCCTTCCGGCTGGCTGGTTTATTGCTGATAAATCTG
GAGCCGGTGAGCGTGGGTCTCGCGGTATCATTGCAGCACTGGGGCCAGATGGTAAGCC
CTCCCGTATCGTAGTTATCTACACGACGGGGAGTCAGGCAACTATGGATGAACGAAATA
GACAGATCGCTGAGATAGGTGCCTCACTGATTAAGCATTGGTAACTGTCAGACCAAGT
TTACTCATATATACTTTAGATTGATTTAAAACTTCATTTTTAATTTAAAAGGATCTAGGTG
AAGATCCTTTTTGATAATCTCATGACCAAAATCCCTTAACGTGAGTTTTCGTTCCACTGA
GCGTCAGACCCCGTAGAAAAGATCAAAGGATCTTCTTGAGATCCTTTTTTTCTGCGCGT
AATCTGCTGCTTGCAAACAAAAAAACCACCGCTACCAGCGGTGGTTTGTTTGCCGGAT
CAAGAGCTACCAACTCTTTTTCCGAAGGTAACTGGCTTCAGCAGAGCGCAGATACCAA
ATACTGTTCTTCTAGTGTAGCCGTAGTTAGGCCACCACTTCAAGAACTCTGTAGCACCG
CCTACATACCTCGCTCTGCTAATCCTGTTACCAGTGGCTGCTGCCAGTGGCGATAAGTC
GTGTCTTACCGGGTTGGACTCAAGACGATAGTTACCGGATAAGGCGCAGCGGTCGGGC
TGAACGGGGGGTTCGTGCACACAGCCCAGCTTGGAGCGAACGACCTACACCGAACTG
AGATACCTACAGCGTGAGCTATGAGAAAGCGCCACGCTTCCCGAAGGGAGAAAGGCG
GACAGGTATCCGGTAAGCGGCAGGGTCGGAACAGGAGAGCGCACGAGGGAGCTTCCA
GGGGGAAACGCCTGGTATCTTTATAGTCCTGTCGGGTTTCGCCACCTCTGACTTGAGCG

TCGATTTTTGTGATGCTCGTCAGGGGGGCGGAGCCTATGGAAAAACGCCAGCAACGCG
GCCTTTTTACGGTTCCTGGCCTTTTGCTGGCCTTTTGCTCACATGTTCTTTCCTGCGTTA
TCCCCTGATTCTGTGGATAACCGTATTACCGCCTTTGAGTGAGCTGATACCGCTCGCCG
CAGCCGAACGACCGAGCGCAGCGAGTCAGTGAGCGAGGAAGCGGAAGA

Sequences used for the affinity analysis of aptamers and transformed PCR(8)

Aptamer-1:

5’-biotin-TCCATCGGGTCATTTA/TPT3/TAATGTAGGT/TPT3/TGGGCTAGGC/TPT3/GCTAGTGGAT

Aptamer-2:

5’-biotin-TCCATCGGGTCATTTAATAATGTAGGT/TPT3/TGGGCTAGGC/TPT3/GCTAGTGGAT

Aptamer-3:

5’-biotin-TCCATCGGGTCATTTAATAATGTAGGTATGGGCTAGGCAGCTAGTGGAT

Sequences used for the transformed PCR(8)

Aptamer-4:

TGTCAATCGATCGTATCAGTCCACTCCATCGGGTCATTTATPT3TAATGTAGGTTPT3TGGGCTAGGCTPT3GCTAGTGGATGCATGACTCGAACGGATTAGTGACT

Primer-F:

TTTTTTTTTTTTTTTTTTTTTTTTTTTTTTTTTTTTTTTTTTTTTTTTTTTTTTTTTTTTTTTCTGTCAATCGATCGTATCAGTCCAC

Primer-R:

TTTTTTTTTTTTTTTTTTTTTTTTTTTTTTTTTTTTTTTTTTTTTTTTTTTTTTTTTTTTAAGTAGTCACTAATCCGTTCGAGTCATGC

Supplementary Figures

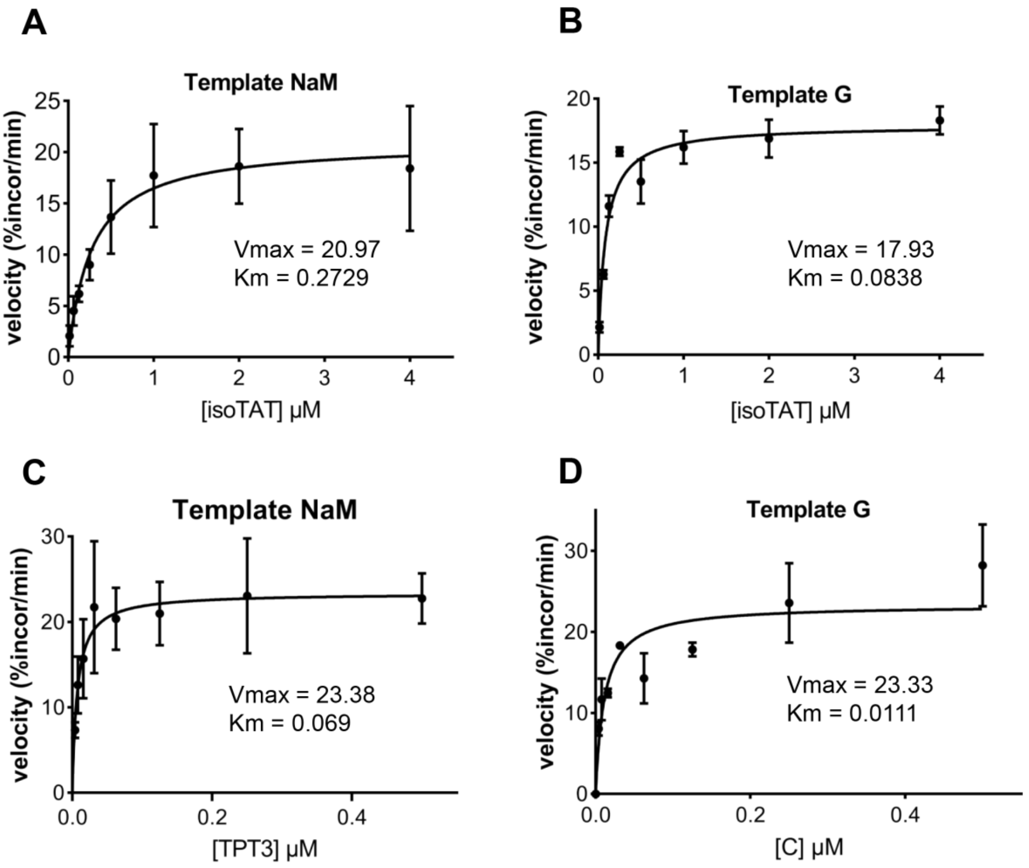

Figure S1. Michaelis-Menten plot. Michaelis-Menten plot for inserting isoTAT (A) or TPT3 (C) opposite NaM in templates; Michaelis-Menten plot for inserting isoTAT (B) or C (D) opposite G in templates.

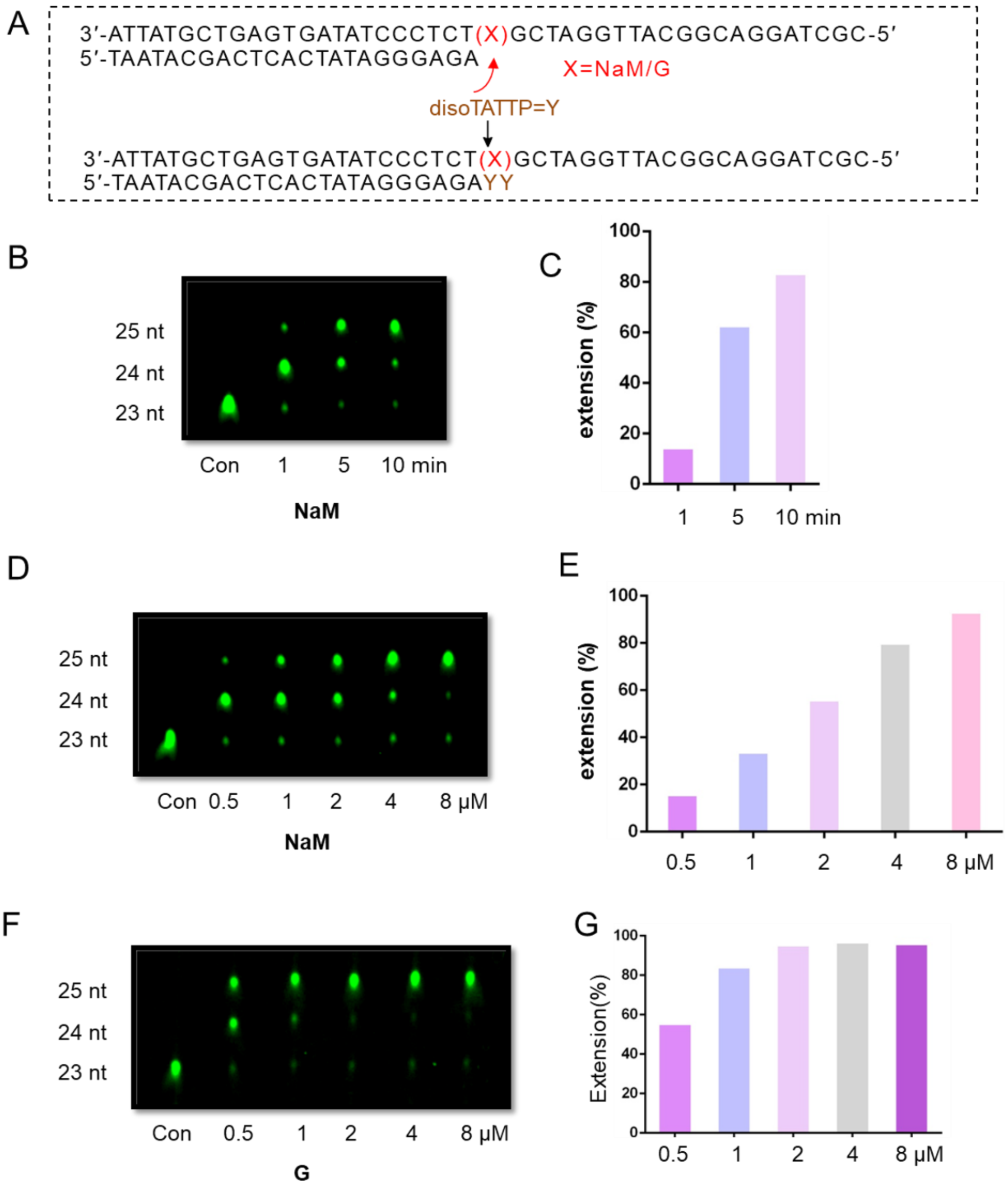

Figure S2. Pre-steady-state kinetic assay of multiple isoTAT insertions opposite NaMG or GG in templates. A, Pre-steady state kinetic scheme for multiple isoTAT insertions. B and D, representative gel for the assays of multiple isoTAT insertions opposite NaMG in the template with concentration-response and time-coursing. C and E, extension level of multiple isoTAT insertions opposite NaMG in the template with concentration-response and time-coursing. F, representative gel for the assays of multiple isoTAT insertions opposite GG in the template with concentration-response. G, extension level of multiple isoTAT insertions opposite GG in the template with concentration-response.

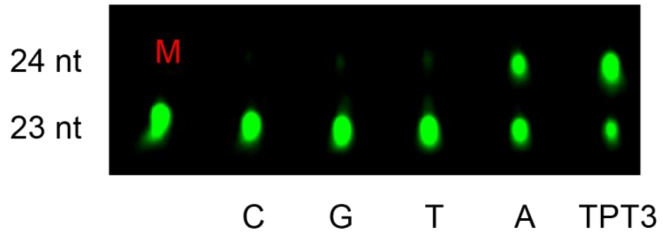

Figure S3. Pre-steady-state assays of insertion of natural nucleotides opposite NaM in the template. TPT3 was used as a control. M: 23 mer primer.

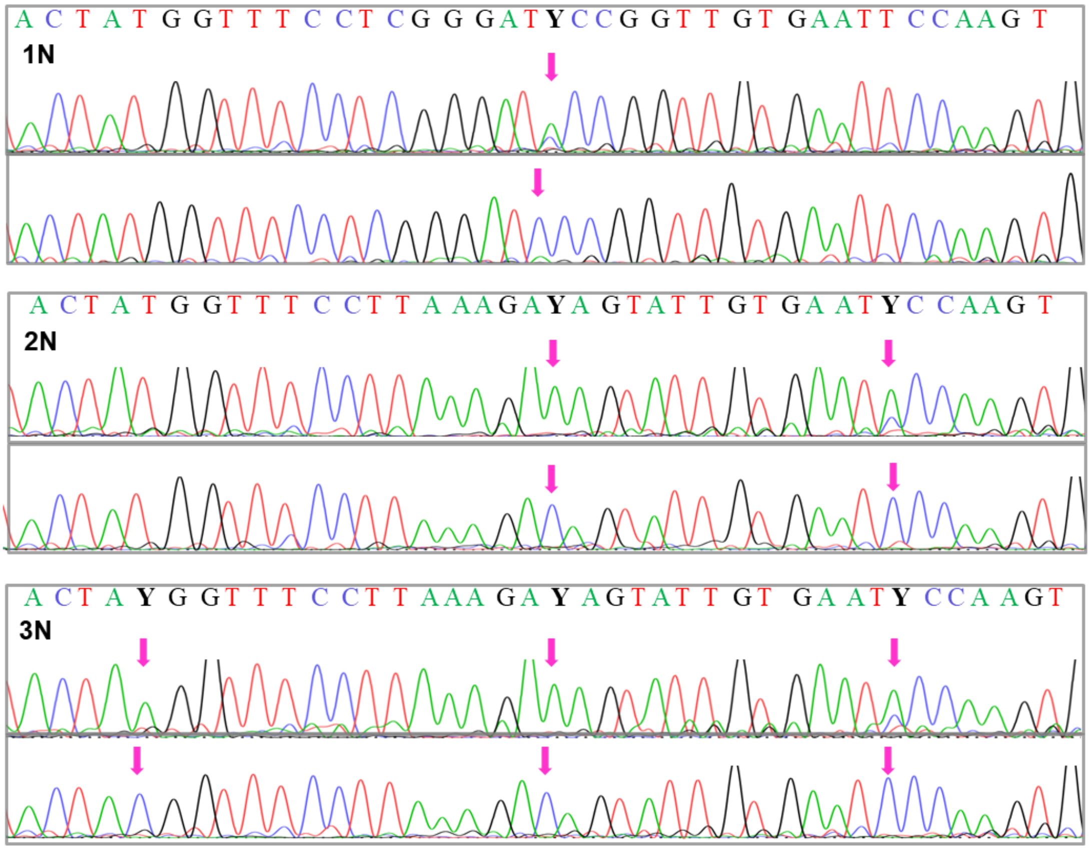

Figure S4. Sequencing assays of transferred PCR with 1N, 2N, and 3N templates. PCR results with isoTAT and NaM showed the transformations of NaM-TPT3 to G-C, and PCR results with only NaM showed the transformations of NaM-TPT3 to T-A. The antisense strands with TPT3 are shown. The red arrowheads indicate the corresponding positions of TPT3 in the templates.

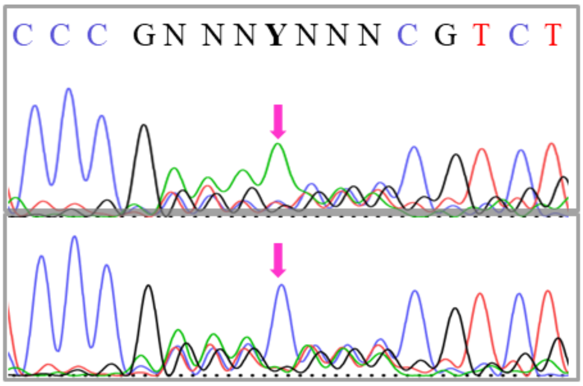

Figure S5. Sequencing assays of transferred PCR with UN template. PCR results with isoTAT and NaM showed the major transformations of NaM-TPT3 to G-C, and PCR results with only NaM showed the major transformations of NaM-TPT3 to T-A. The antisense strands with TPT3 are shown. The red arrowheads indicate the corresponding positions of TPT3 in the templates.

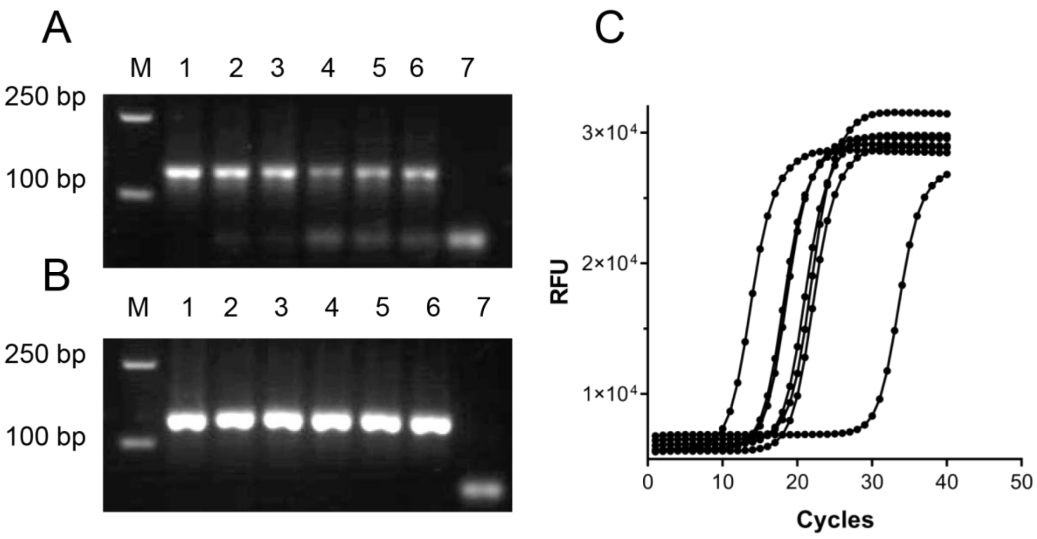

Figure S6. Quantitative real time PCR (qPCR) analysis for the transferred PCR. (A) Representative agarose gel for qPCR with isoTAT and NaM. (B) Representative agarose gel for qPCR with only NaM. M: marker; 1-7 indicated qPCR with 400 pg, 40 pg, 4 pg, 0.4 pg, 0.04 pg, 0.004 pg, and 0 pg of template 1N. (C) 40 cycle qPCR amplification of (from left to right) 400 pg, 40 pg, 4 pg, 0.4 pg, 0.04 pg, and 0.004 pg of template 1N with only NaM. Amplification without template as a negative control is the rightmost curve signal resulting in primer-dimers.

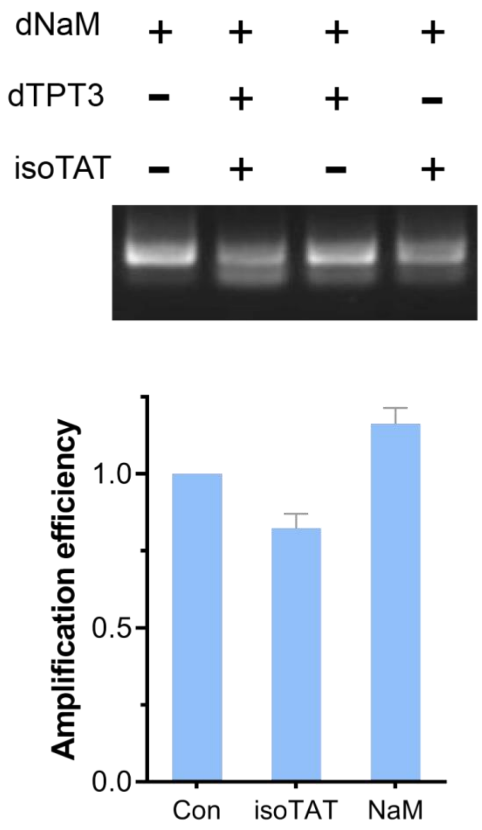

Figure S7. Analysis of the amplified efficiency of PCR with isoTAT or only NaM after 36 cycles. The amplified efficiency of dTPT3 and dNaM was used as control. The data are averages and standard deviations of three independent determinations.

A

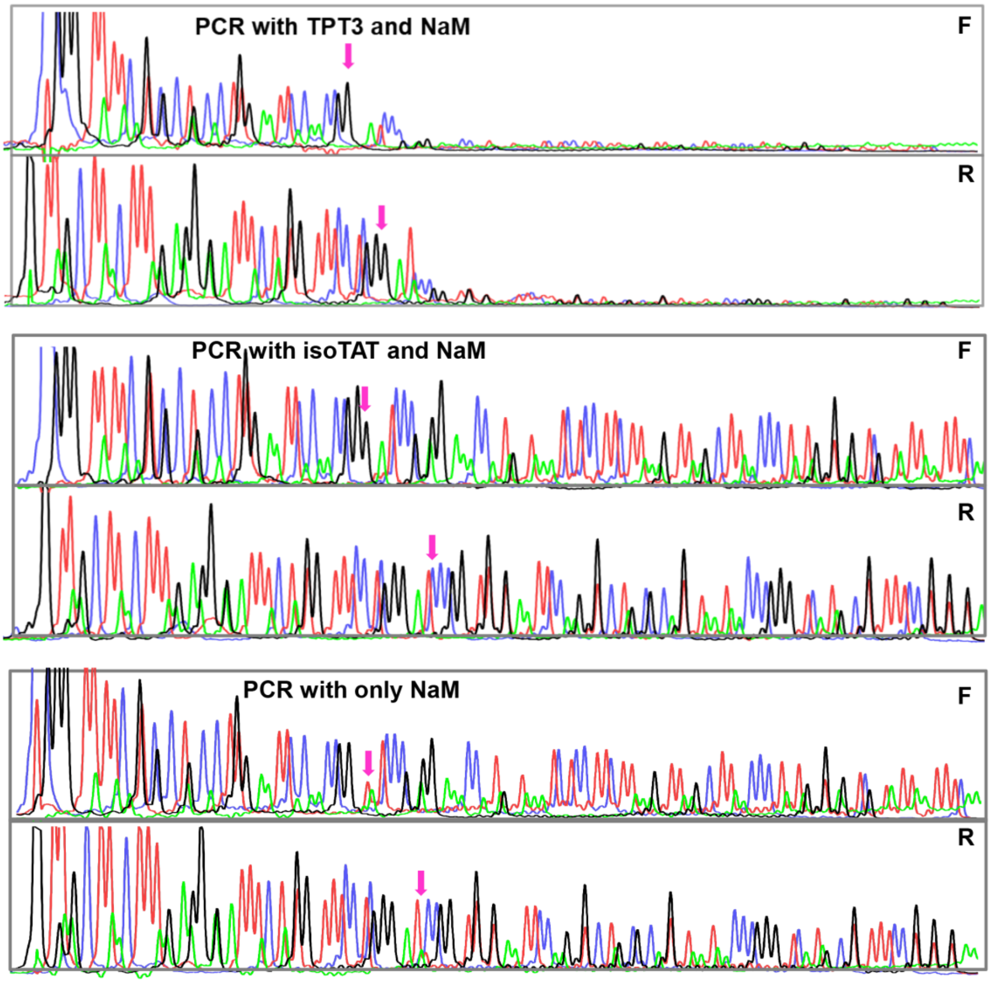

B

1N-F

```
0:  CCCGGGTTATTACATGCGCTAGCACTTGGAATTCACAACCGGGATCCCGAGGAAACCATA  :  60
1:  CCCGGGTTATTACATGCGCTAGCACTTGGAATTCACAACCGGXATCCCGAGGAAACCATA  :  60

0:  GTAAATCTCCTTCTTAAAGTTAAGCTTAACCCTATAGTGAGTCGTATTAATTTC  :  114
1:  GTAAATCTCCTTCTTAAAGTTAAGCTTAACCCTATAGTGAGTCGTATTAATTTC  :  114
```

1N-R

```
0:  GGGTTAAGCTTAACTTTAAGAAGGAGATTTACTATGGTTTCCTCGGGATCCCGGTTGTGA  :  60
1:  GGGTTAAGCTTAACTTTAAGAAGGAGATTTACTATGGTTTCCTCGGGATYCCGGTTGTGA  :  60

0:  ATTCCAAGTGCTAGCGCATGTAATAACCCGGGTCATAGCTGTTTCCTGTGTG  :  112
1:  ATTCCAAGTGCTAGCGCATGTAATAACCCGGGTCATAGCTGTTTCCTGTGTG  :  112
```

Figure S8. Sequencing assays of transferred PCR with 1N template. (A) Raw sequencing data of PCR with TPT3 and NaM, isoTAT and NaM, as well as only NaM. PCR results with isoTAT and NaM did not show signal attenuation. The red arrowheads indicate the corresponding positions of NaM or TPT3 in the templates. (B) Sequence alignment between PCR results with isoTAT and NaM and the synthetic template. 0: PCR with isoTAT and NaM; 1: the synthetic template; X: NaM; Y: TPT3.

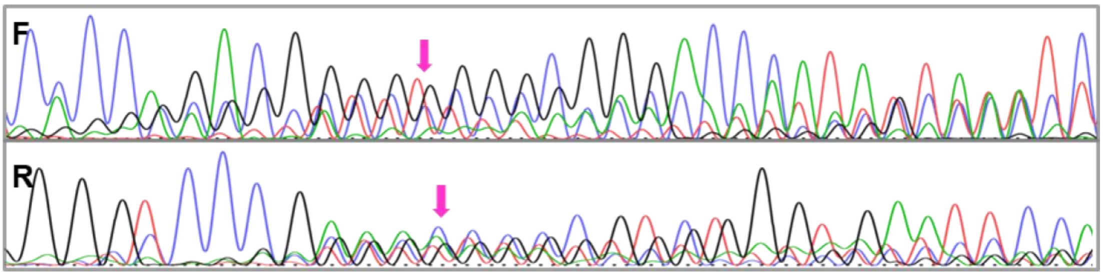

Figure S9. Sequencing assays of PCR with TPT3 and four dNTPs using UN templates. The red arrowheads indicate the corresponding positions of NaM or TPT3 in the templates.

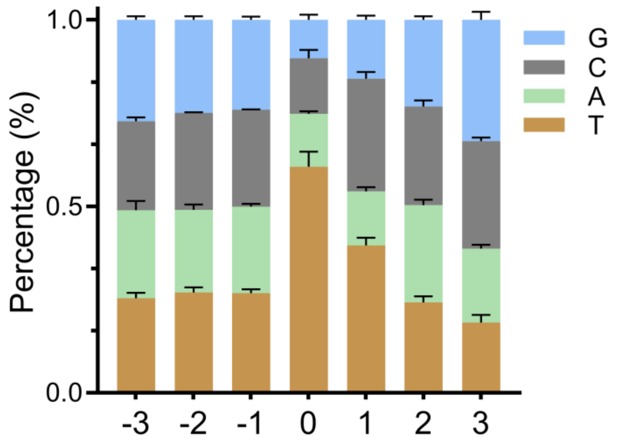

Figure S10. The distribution frequency of natural bases in each random locus and NaM locus medicated by only NaM.

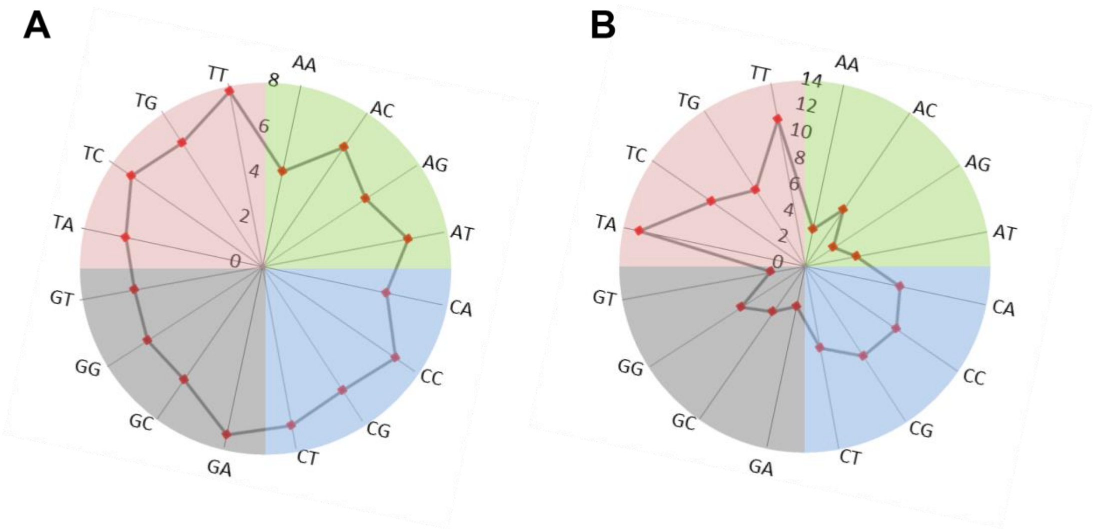

Figure S11. The distribution frequency of dinucleotides in the bridge-base transformation. (A) Dinucleotides upstream. (B) Dinucleotides downstream. The

coordinate unit is 10000.

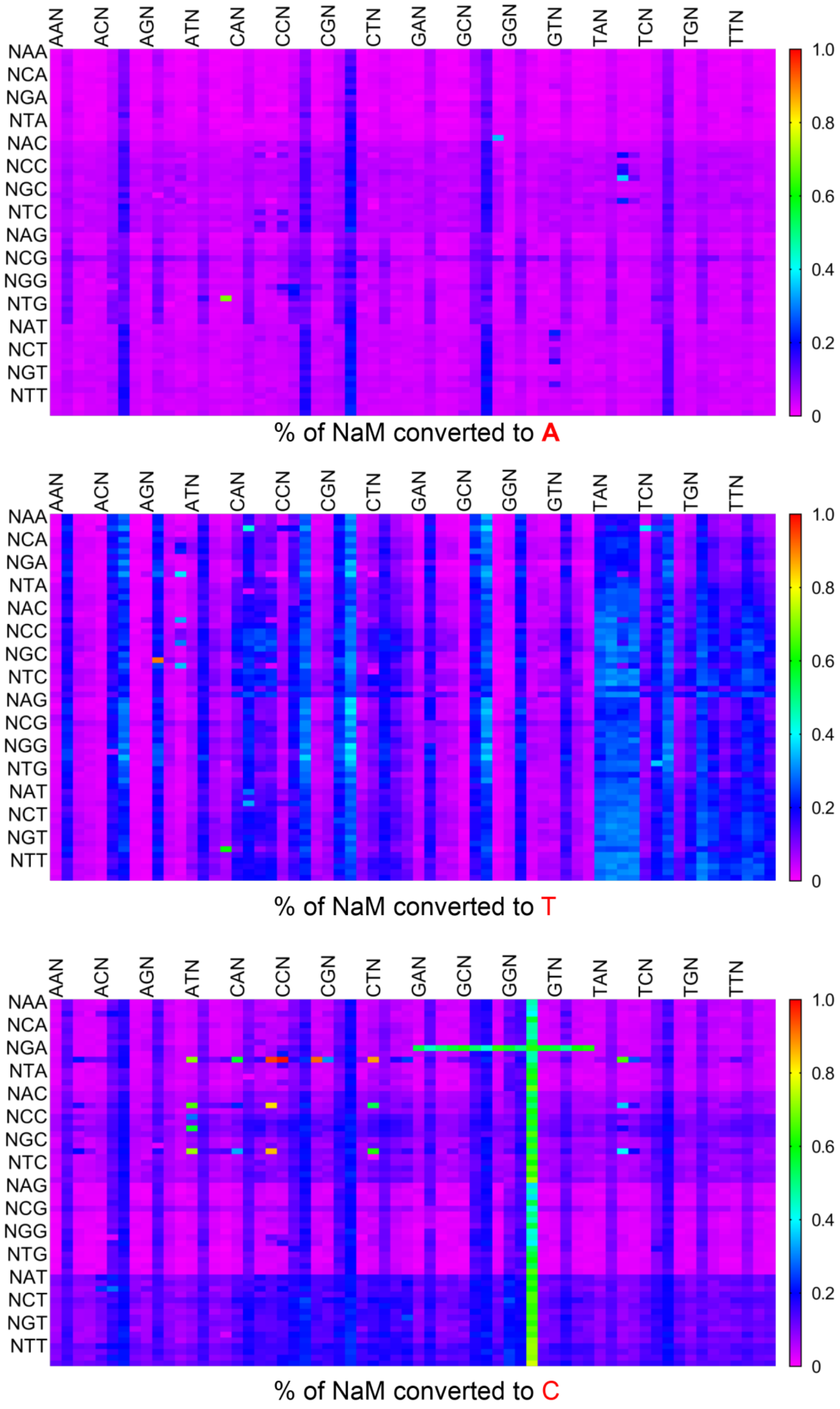

Figure S12. % of NaM to A, T, or C transformation medicated by isoTAT and NaM in each sequence context. The third bases upstream (left) and downstream (up) of unnatural base are shown as "N" and arranged in the order of A, C, G, and T in each

dinucleotide combination.

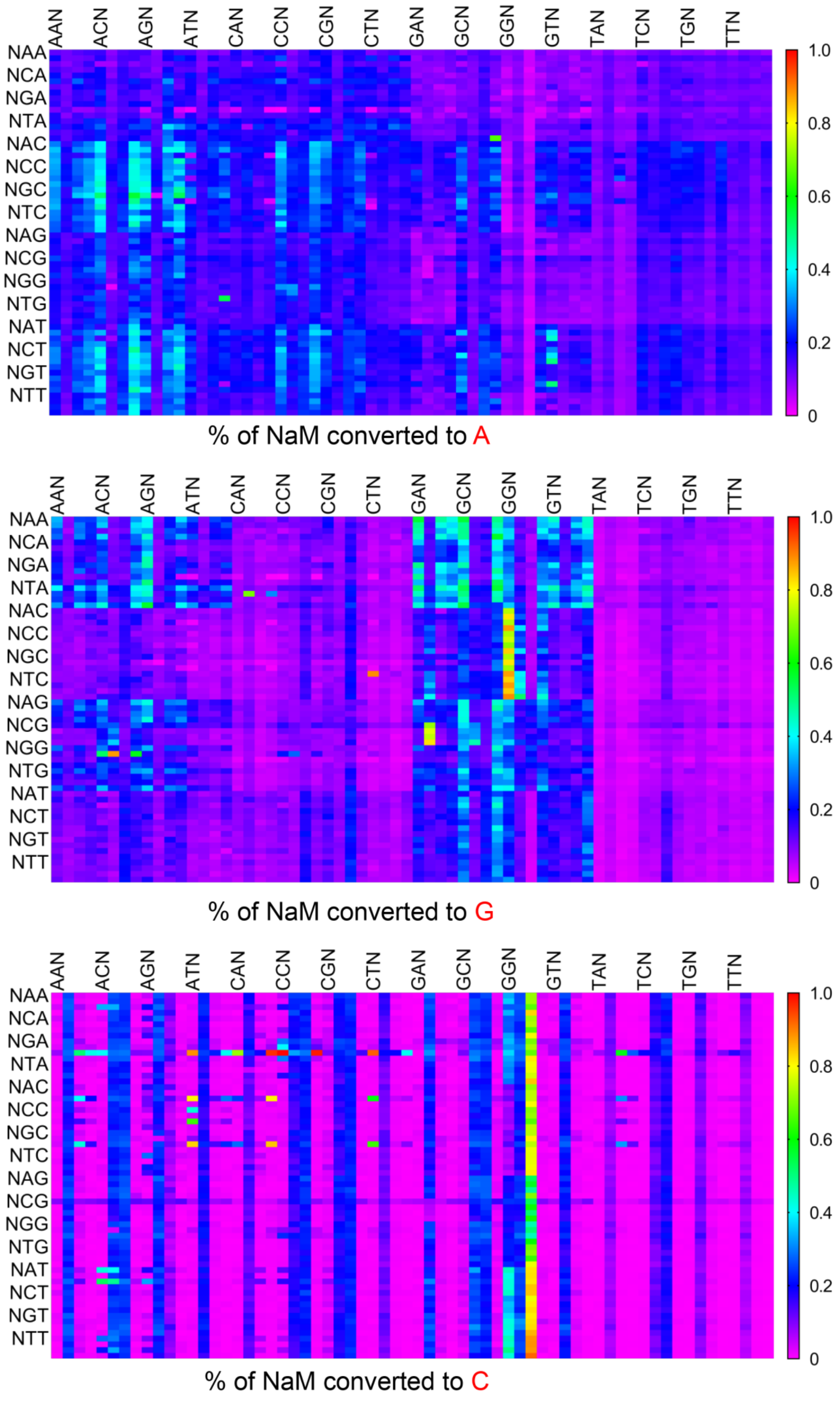

Figure S13. % of NaM to A, G, or C transformation medicated by only NaM in each sequence context. The third bases upstream (left) and downstream (up) of unnatural base are shown as “N” and arranged in the order of A, C, G, and T in each

dinucleotide combination.

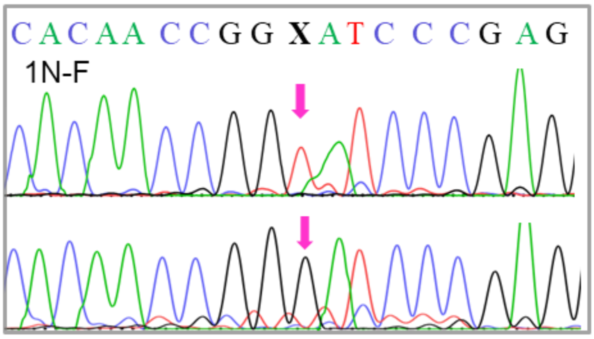

Figure S14. Sequencing assays of the plasmid that contained one UBP medicated by transferred PCR. PCR with isoTAT and NaM showed the transformations of NaM-TPT3 to G-C(below), and PCR with only NaM showed the transformations of NaM-TPT3 to T-A(above). The sense strands are shown. The red arrowheads indicate the corresponding positions of NaM in the templates.

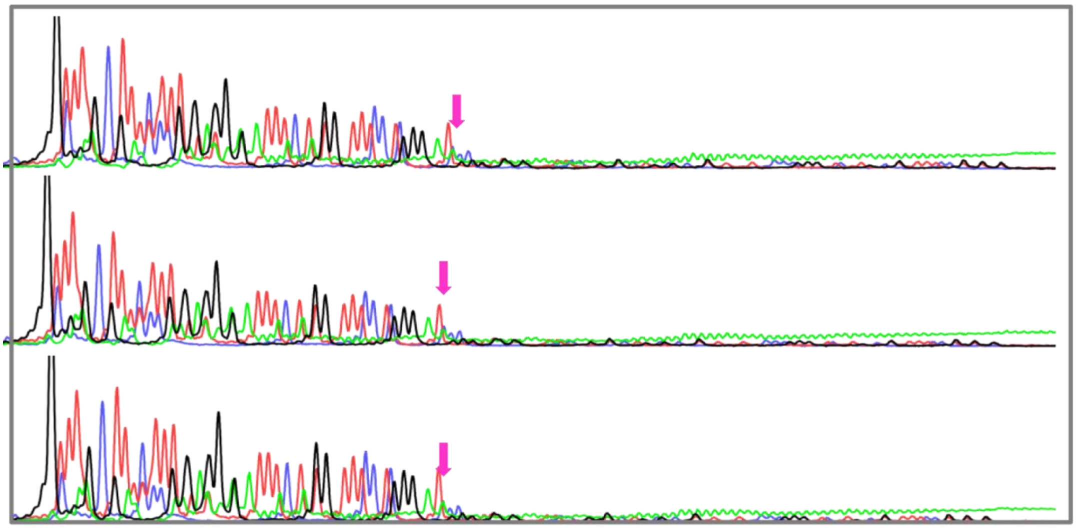

Figure S15. Raw sequencing data of PCR with NaM and TPT3 using the plasmid that contained one UBP as the template. The antisense strands with three replicates are shown and are used for calculating retention. The red arrowheads indicate the corresponding positions of TPT3 in the templates.

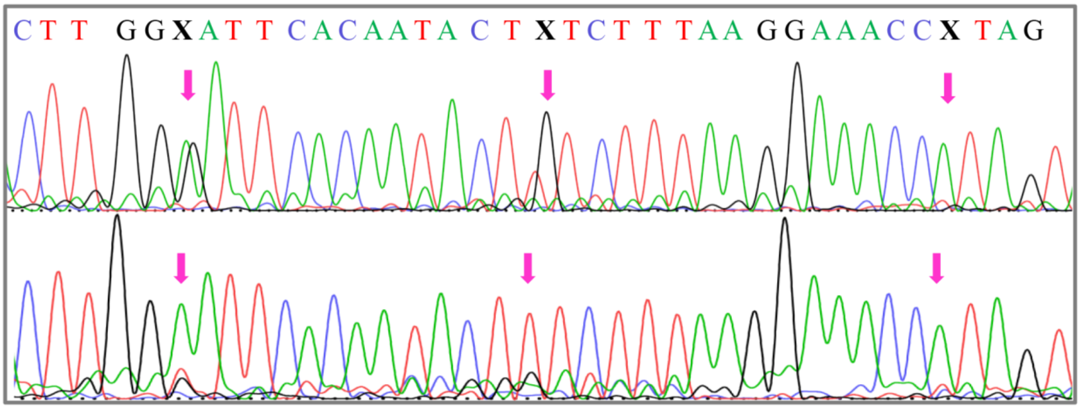

Figure S16. Sequencing assays of the plasmid that contained three UBPs medicated by transferred PCR. Up: PCR with NaM and isoTAT; down: PCR with only NaM. The sense strands are shown. The red arrowheads indicate the corresponding positions of NaM in the templates.

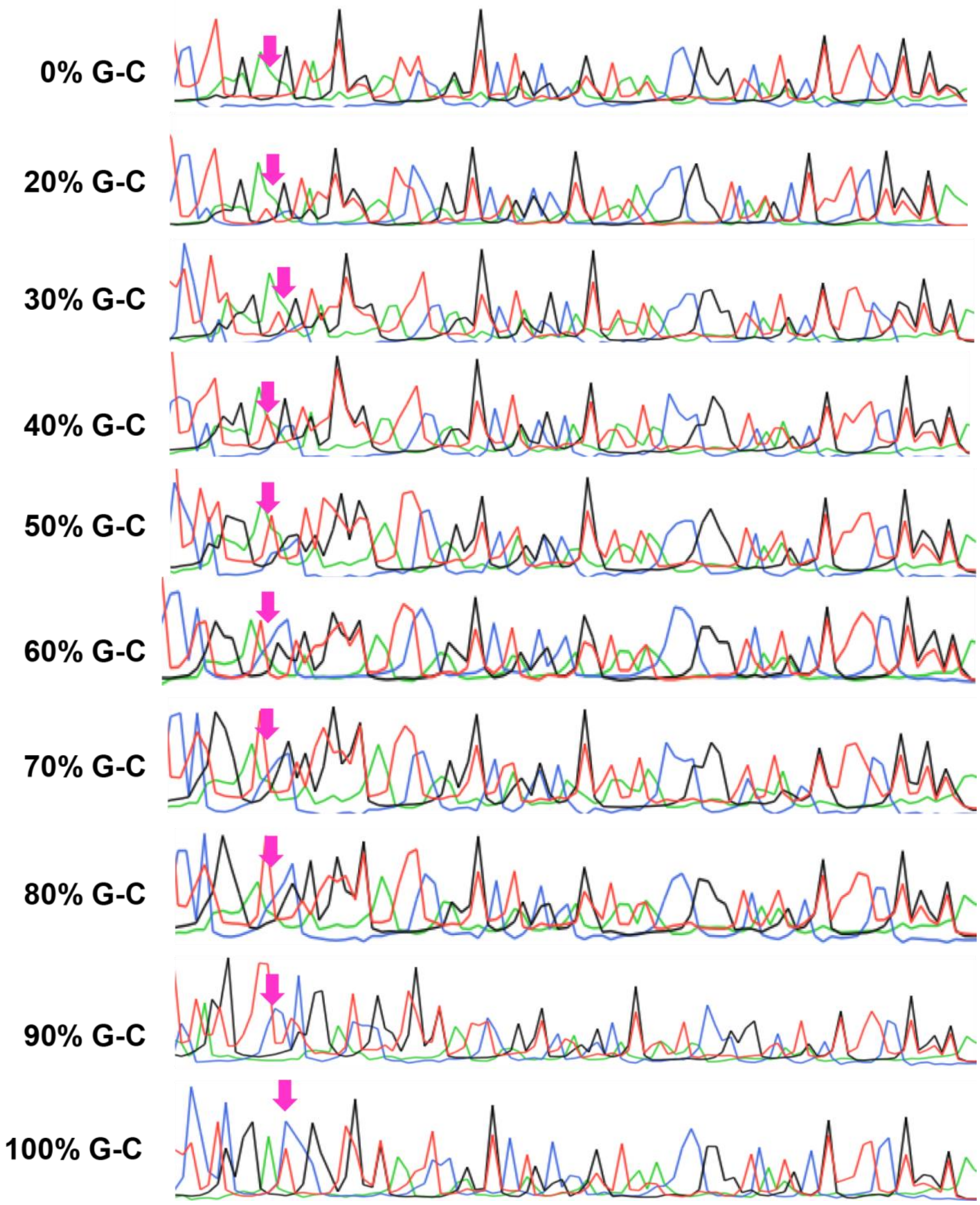

Figure S17. Representative raw sequencing data of mixtures of bridge PCR products (1N) and natural sequences in different proportions. The proportions of bridge PCR products are 0, 20, 30, 40, 50, 60 70, 80, 90, and 100%. The antisense strands are shown. The red arrowheads indicate the corresponding positions being transferred to C (blue). C signals downstream (right) used for calculation are also shown.

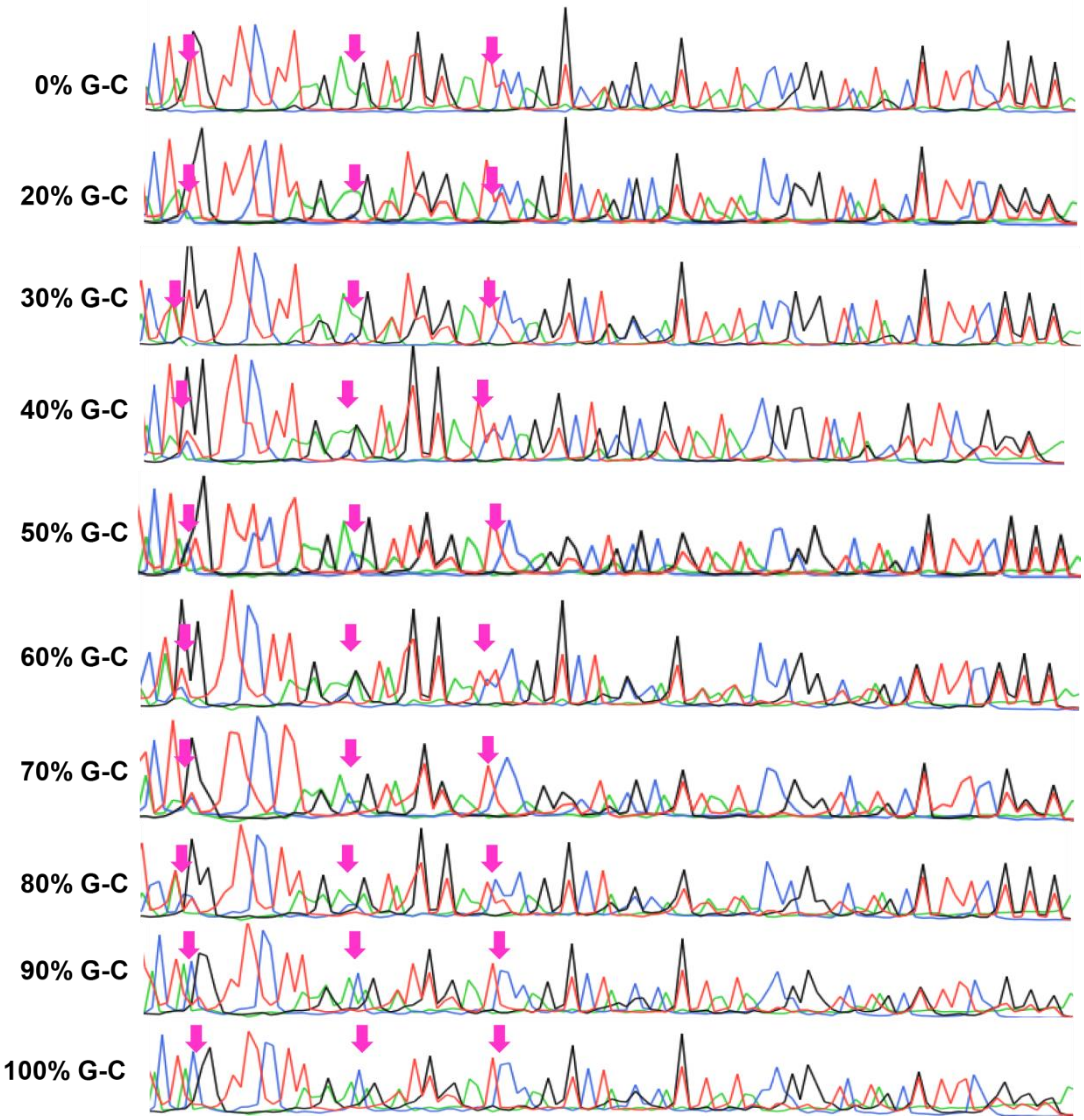

Figure S18. Representative raw sequencing data of mixtures of bridge PCR products (3N) and natural sequences in different proportions. The proportions of bridge PCR products are 0, 20, 30, 40, 50, 60 70, 80, 90, and 100%. The antisense strands are shown. The red arrowheads indicate the corresponding positions being transferred to C (blue). C signals downstream (right) of the third C are used for calculation.

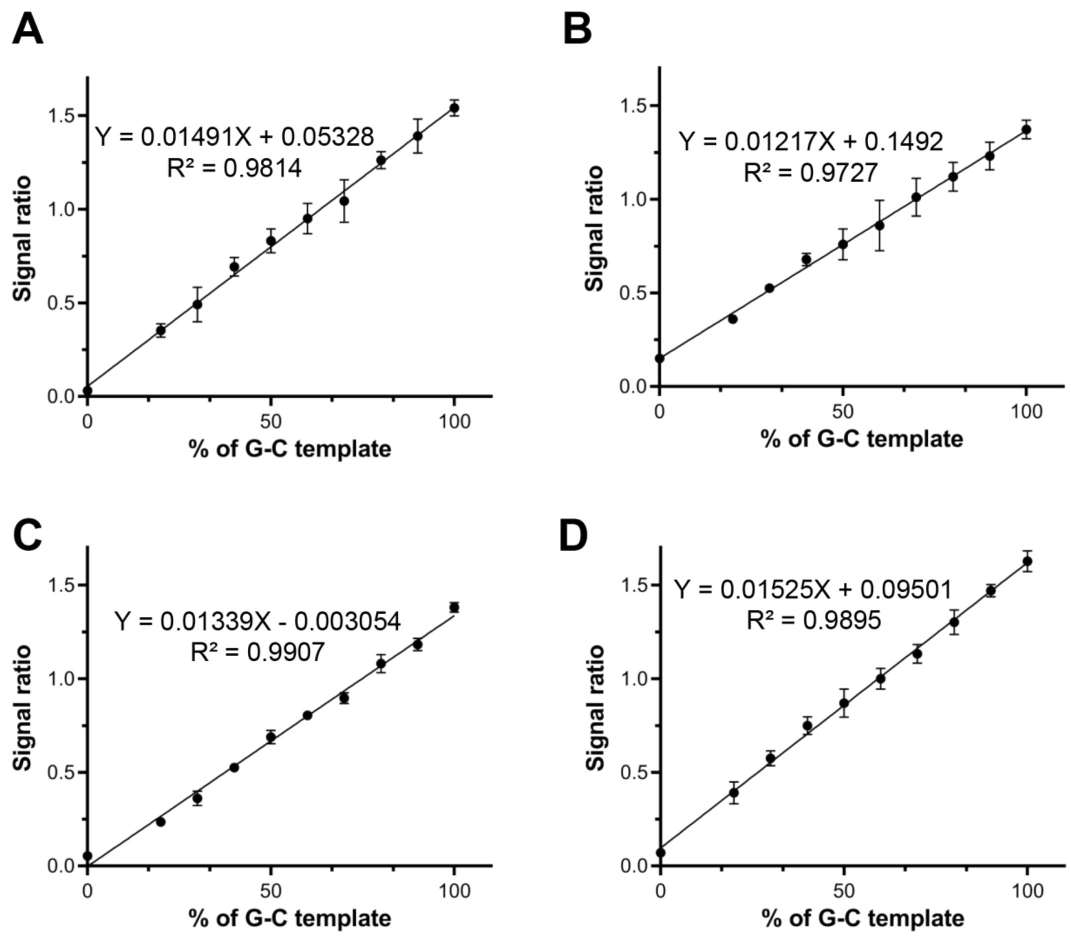

Figure S19. Standard curve of NaM-TPT3 to G-C transformation in 1N template (A), and 3N template with the sequence context GXA (B), TXT (C), and CXT (D). The formula of standard curve is also shown.

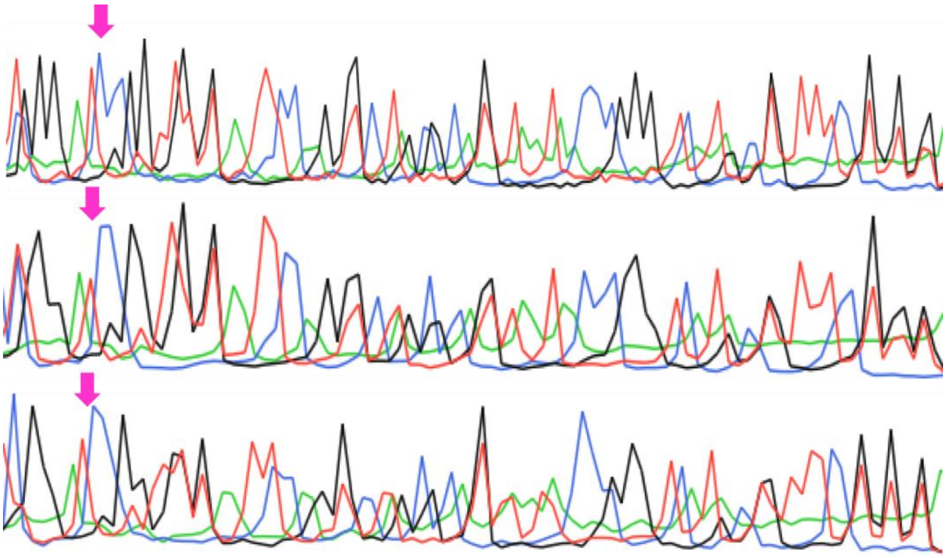

Figure S20. Raw sequencing data for bridge PCR with the plasmid that contained one UBP. The antisense strands with three replicates are shown and are used for calculating retention. The red arrowheads indicate the corresponding positions being transferred to C (blue).

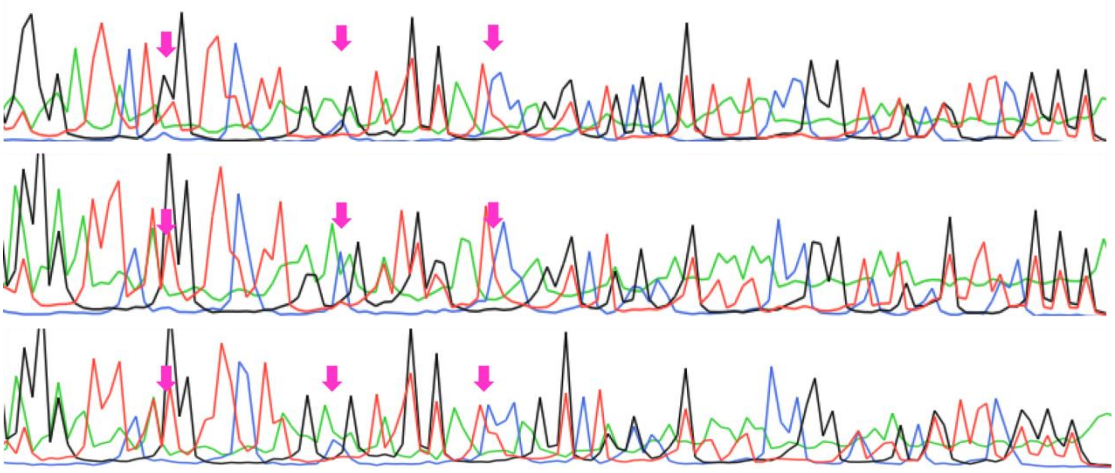

Figure S21. Raw sequencing data for bridge PCR with the plasmid that contained three UBPs. The antisense strands with three replicates are shown and are used for calculating retention. The red arrowheads indicate the corresponding positions being transferred to C (blue).

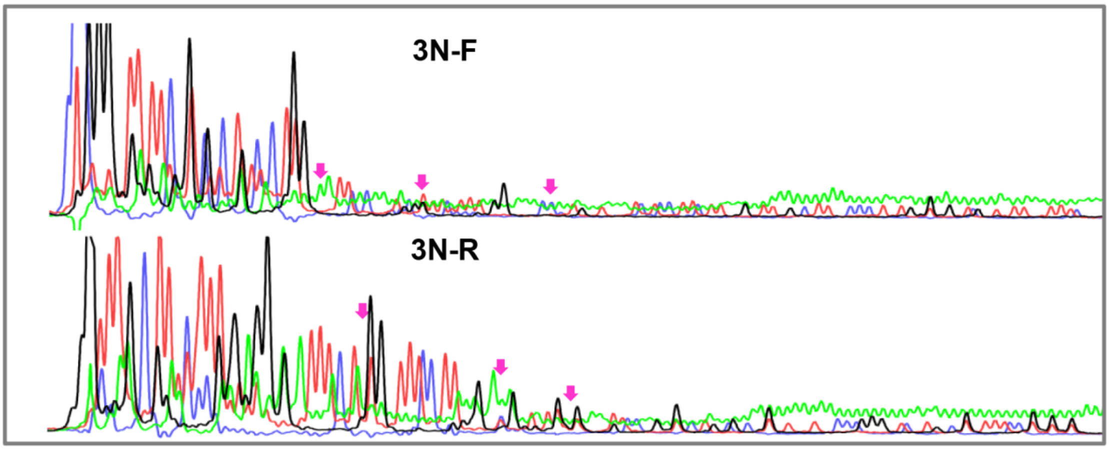

Figure S22. Raw sequencing data for PCR with NaM and TPT3 with the plasmid that contained three UBPs. The sense (F) and antisense stands (R) are shown. The red arrowheads indicate the corresponding positions of NaM or TPT3 in the templates.

3N-F

```
0: CGGGTTATTACATGCGCTAGCACTTGGGATTCACAATACTGTCTTTAAGGAAACCATAGT : 60
1: CGGGTTATTACATGCGCTAGCACTTGGXATTCACAATACTXTCTTTAAGGAAACCXTAGT : 60

0: AAATCTCCTTCTTAAAGTTAAGCTTAACCCTATAGTGAGTCGTATTAATTTC : 112
1: AAATCTCCTTCTTAAAGTTAAGCTTAACCCTATAGTGAGTCGTATTAATTTC : 112
```

3N-R

```
0: GTTAAGCTTAACTTTAAGAAGGAGATTTACTATGGTTTCCTTAAAGACAGTATTGTGAAT : 60
1: GTTAAGCTTAACTTTAAGAAGGAGATTTACTAYGGTTTCCTTAAAGAYAGTATTGTGAAT : 60

0: CCCAAGTGCTAGCGCATGTAATAACCCGGGTCATAGCTGTTTCCTGTGTG : 110
1: YCCAAGTGCTAGCGCATGTAATAACCCGGGTCATAGCTGTTTCCTGTGTG : 110
```

Figure S23. Sequence alignment between PCR results with isoTAT and NaM after in vivo replication and the synthetic template. 0: PCR results with isoTAT and NaM after in vivo replication; 1: the synthetic template; X: NaM; Y: TPT3.

A

Templates
1: ATTGGATCG/NaM/GGGC*GGGACCCATAGTAAATCT*
3: ATTGGATCG/NaM/GGGC*CCTATAGTGAGTCGTATTA*

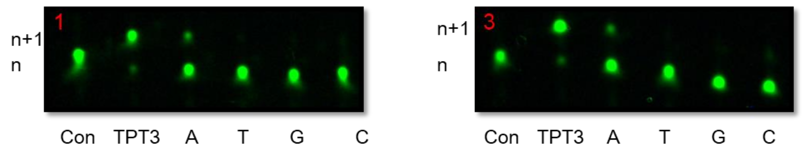

B

Serial NO	Mispairing rate(%)
1	22.72±3.43
3	20.653±2.24

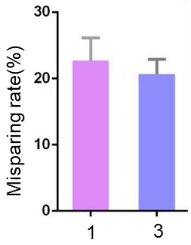

C

Templates
2: ATTGGAACC/NaM/TAG*TAAATCTCCTTCTTAAAGTTA*
9: ATTGGAACC/NaM/TAG*CCCTATAGTGAGTCGTATTA*

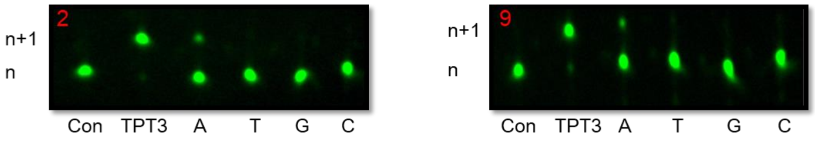

D

Serial NO	Mispairing rate(%)
2	19.26±2.03
9	14.37±2.97

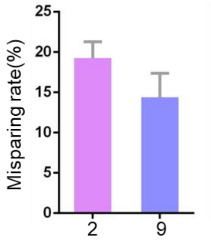

Figure S24. Mispairing analysis of templates 1 and 3 as well as 2 and 9 with farther flanking sequence variation. A and C, representative gels for mispairing. B and D, mispairing levels with illustrations.

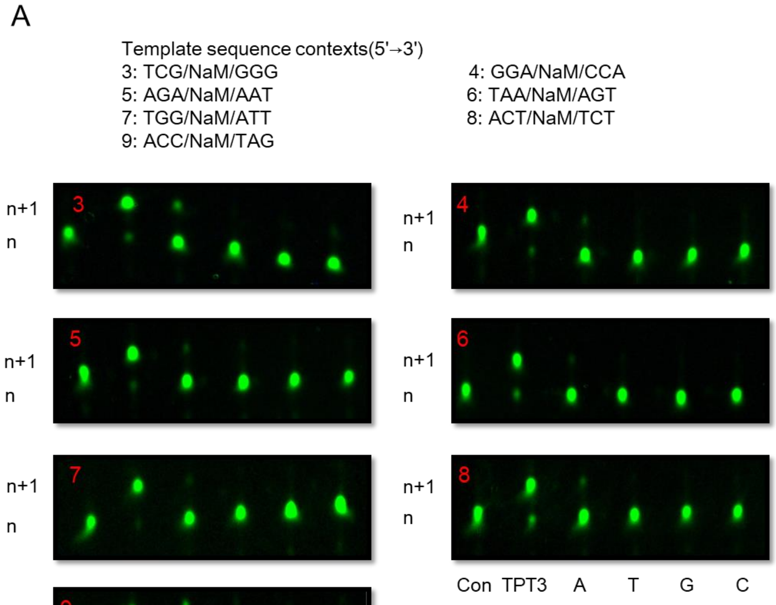

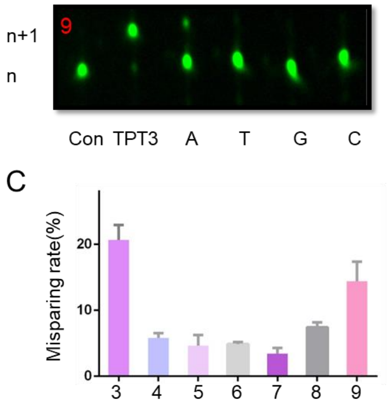

B

Serial NO	Mispairing rate (%)
3	20.65±2.24
4	5.83±0.72
5	4.66±1.57
6	4.88±0.31
7	3.45±0.83
8	7.41±0.73
9	14.37±2.97

Figure S25. Mispairing analysis of templates 3-9 with three flanking sequence variations upstream and downstream of the NaM location. A, representative gel for mispairing. B and C, mispairing level with illustrations.

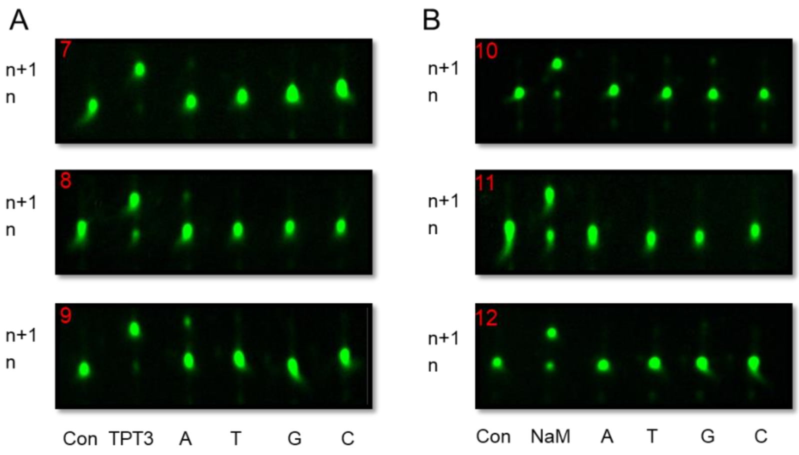

C

Serial NO	Mispairing rate (%)
7+10	10.64 ± 0.72
8+11	7.41± 0.73
9+12	16.56 ± 3.17

D

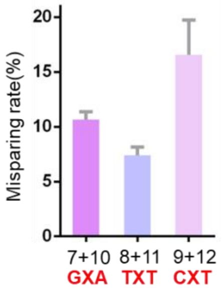

Figure S26. Mispairing analysis of templates with three flanking sequence variations upstream and downstream of the NaM-TPT3 loci. A and B, representative gels for pairing and mispairing with NaM and TPT3 in templates, respectively. C and D, mispairing level with illustrations. Three flanking sequence variations upstream and downstream of the NaM or TPT3 loci were the same as the sequence contents used for in vivo replication. Mispairing levels of NaM and TPT3 with natural bases in one site were added together as total mispairing rates. 7+10 (GXA), 8+11 (TXT), and 9+12 (CXT) were the corresponding loci.

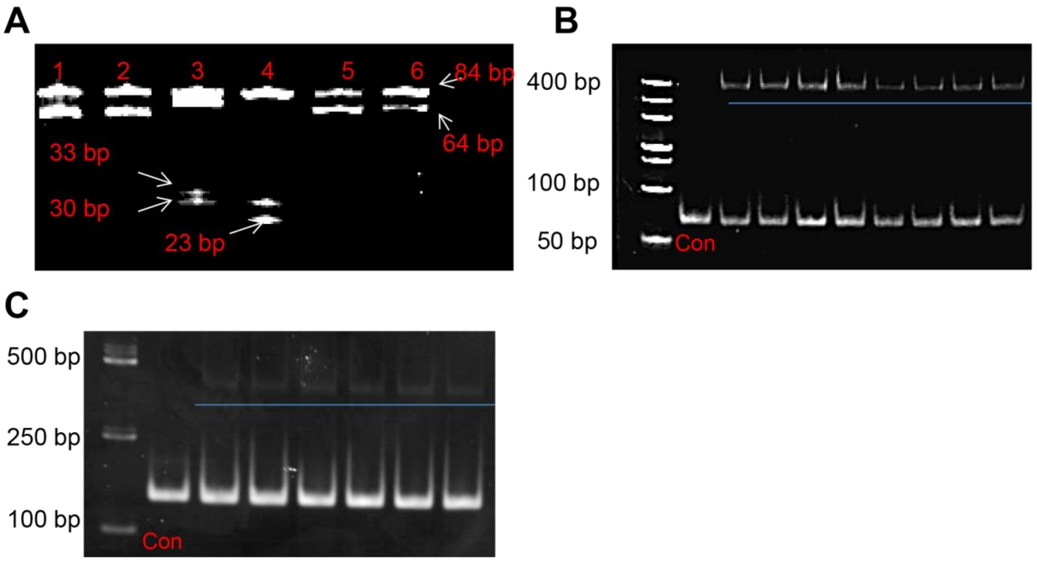

Figure S27. Representative gels for monitoring the labeling of lesions with TPT3 and the enrichment of marked lesions. (A) Representative PAGE gels for monitoring the labeling of lesions with TPT3 on the KRAS-1U and KRAS-2U duplex. Lane-1 and lane-2 represented the initial size of KRAS-1U and KRAS-2U, respectively. Lane-3 and lane-4 indicated that KRAS-1U and KRAS-2U were broken at the sites of dU. Lane-5 and lane-6 confirmed that the broken DNA strand could be repaired by dTPT3TP and fused into a completed DNA strand. (B, C) Representative PAGE gels for biotin-shift enrichment of KRAS-1U and 134-2U after PCR with $TPT3^{biotin}$ and NaM. Con represents that products are without the addition of streptavidin and others are incubated with streptavidin. The slower migrating bands were marked with blue lines and were enriched.

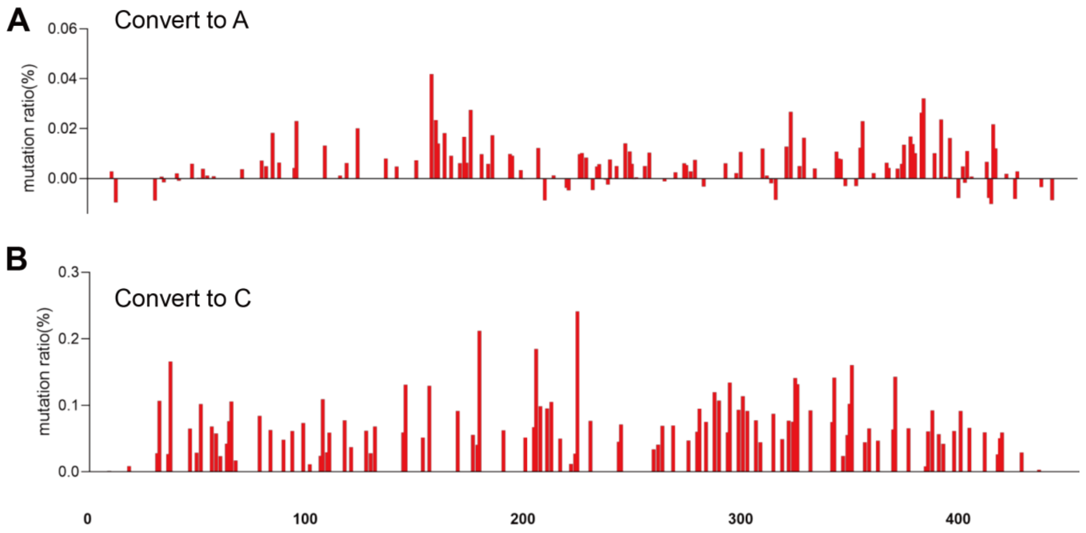

Figure S28. Mutation ratios of C on the antisense strand and A on the sense strand from Deep sequencing data of DNA fragments in the plasmid after transformed PCR. The distribution is also shown.

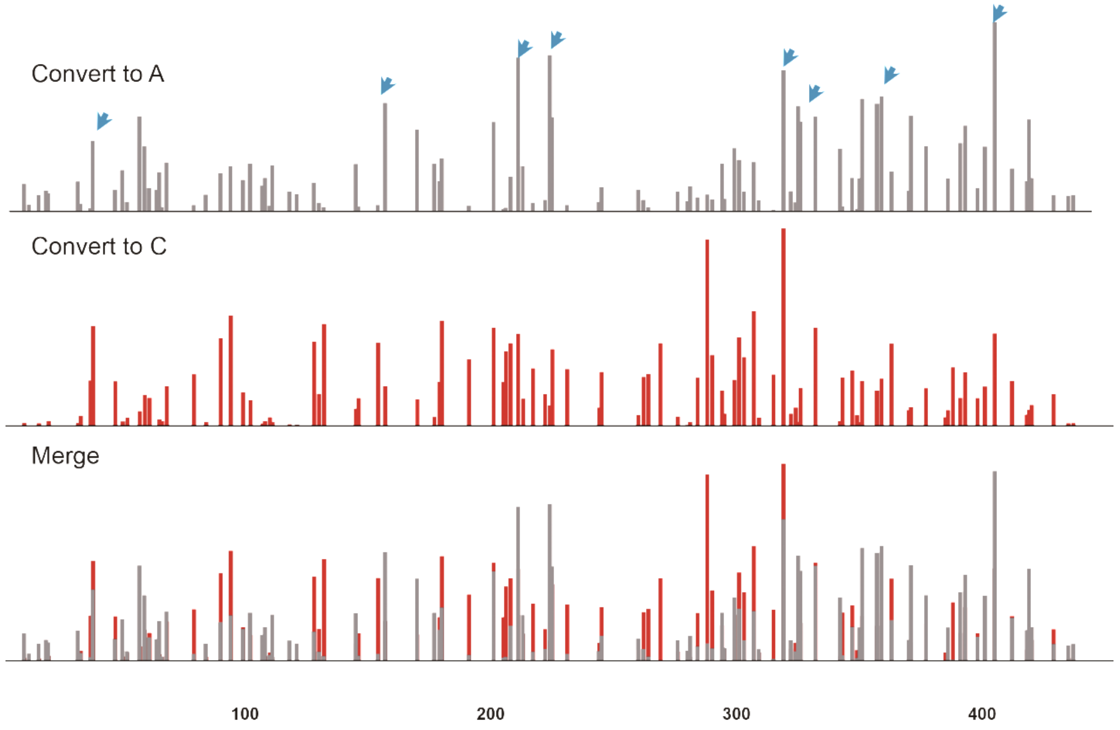

Figure S29. Mutation ratios of T on antisense strand from Deep sequencing data of DNA fragments in the plasmid after transformed PCR. Blue arrowheads indicate sites increased most with overlap. The distribution is also shown.

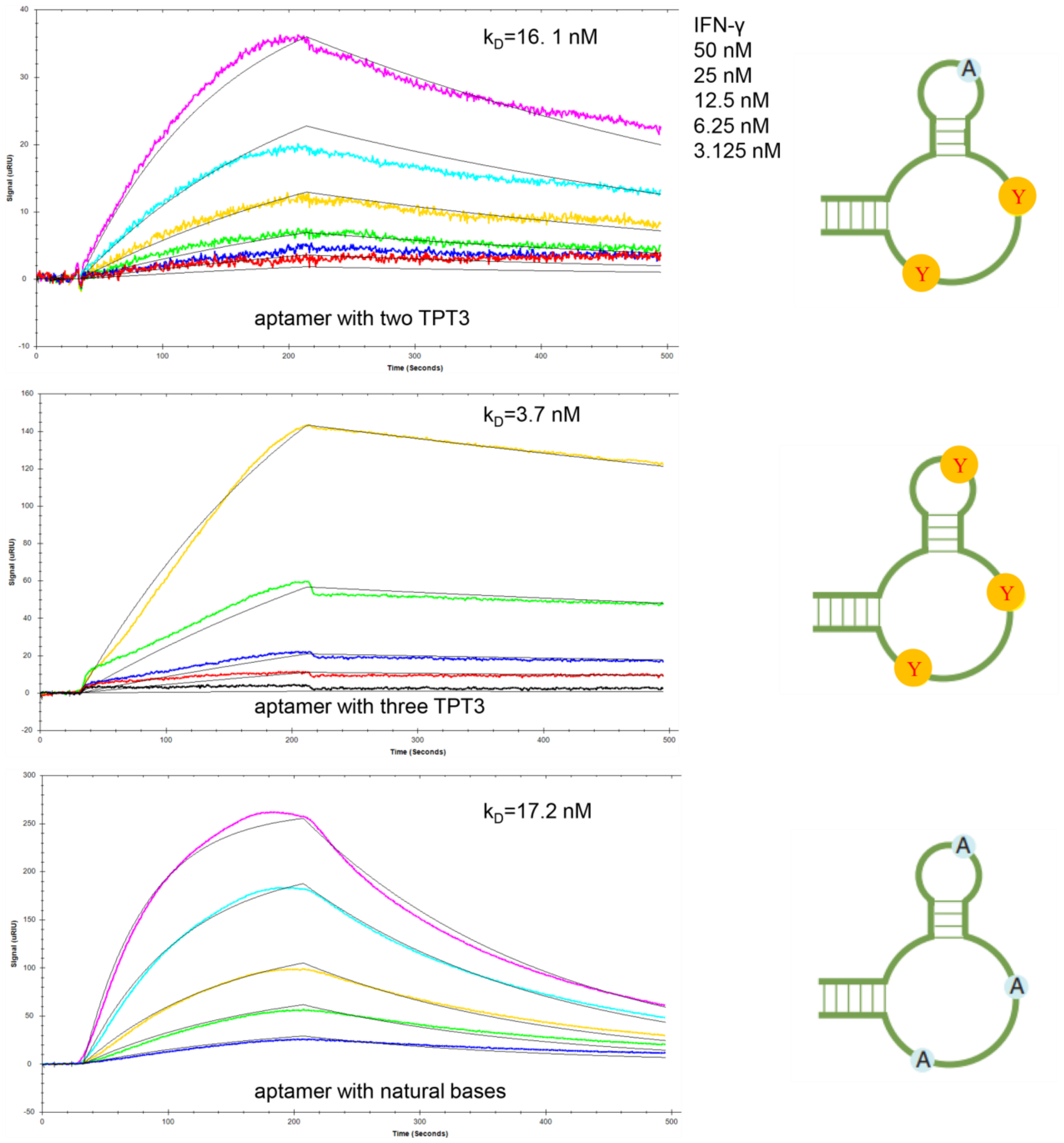

Figure S30. The affinity of the aptamers with TPT3 or natural bases to IFN-γ measured by SPR. Potential structures of these aptamers according to report(8). Y: TPT3.

References

1. Asagarasu, A., Matsui, T., Hayashi, H., Tamaoki, S., Yamauchi, Y. and Sato, M. (2009) Design and synthesis of piperazinylpyridine derivatives as novel 5-HT1A agonists/5-HT3 antagonists for the treatment of irritable bowel syndrome (IBS). *Chem Pharm Bull (Tokyo)*, **57**, 34-42.
2. New, J.S., Christopher, W.L., Yevich, J.P., Butler, R., Schlemmer, R.F., Jr., VanderMaelen, C.P. and Cipollina, J.A. (1989) The thieno[3,2-c]pyridine and furo[3,2-c]pyridine rings: new pharmacophores with potential antipsychotic activity. *J Med Chem*, **32**, 1147-1156.
3. Seo, Y.J., Hwang, G.T., Ordoukhanian, P. and Romesberg, F.E. (2009) Optimization of an unnatural base pair toward natural-like replication. *Journal of the American Chemical Society*, **131**, 3246-3252.
4. Malyshev, D.A., Seo, Y.J., Ordoukhanian, P. and Romesberg, F.E. (2009) PCR with an

expanded genetic alphabet. *Journal of the American Chemical Society*, **131**, 14620-14621.

5. Wang, H., Wang, L., Ma, N., Zhu, W., Huo, B., Zhu, A. and Li, L. (2022) Access to Photostability-Enhanced Unnatural Base Pairs via Local Structural Modifications. *ACS synthetic biology*, **11**, 334-342.
6. Riedl, J., Ding, Y., Fleming, A.M. and Burrows, C.J. (2015) Identification of DNA lesions using a third base pair for amplification and nanopore sequencing. *Nat Commun*, **6**.
7. Zhang, Y., Lamb, B.M., Feldman, A.W., Zhou, A.X., Lavergne, T., Li, L. and Romesberg, F.E. (2017) A semisynthetic organism engineered for the stable expansion of the genetic alphabet. *Proceedings of the National Academy of Sciences of the United States of America*, **114**, 1317-1322.
8. Kimoto, M., Yamashige, R., Matsunaga, K., Yokoyama, S. and Hirao, I. (2013) Generation of high-affinity DNA aptamers using an expanded genetic alphabet. *Nature biotechnology*, **31**, 453-457.
